# Outcomes of gastrostomy in children with neurological impairment: a systematic review

**DOI:** 10.3389/fped.2026.1963093

**Published:** 2026-10-12

**Authors:** Mariam Kamel Ibrahim, Hafeeza Jummakhan, Malak Dawood, Mariam Siam, Minat Allah Alhusami, Sara Khan, Jonathan Mokhtar, Hani T. S. Benamer, Mohamed O. E. Babiker

**Affiliations:** 1 College of Medicine, Mohammed Bin Rashid University of Medicine and Health Sciences, Dubai Health, Dubai, United Arab Emirates; 2 Research and Graduate Studies, Mohammed Bin Rashid University of Medicine and Health Sciences (MBRU), Dubai Health, Dubai, United Arab Emirates; 3 Pediatrics, Dubai Health, Dubai, United Arab Emirates

**Keywords:** aspiration pneumonia, cerebral palsy, children, dysphagia, enteral nutrition, gastrostomy, neurological impairment, systematic review

## Abstract

**Introduction:**

Gastrostomy feeding is widely used for children with neurological impairment and inadequate oral intake, but evidence across family-important outcomes has not been synthesised. Previous systematic reviews were restricted to cerebral palsy and found no controlled studies. The objective of this study is to assess gastrostomy vs. continued oral/nasogastric feeding on nutritional, respiratory, neurodevelopmental, and caregiver-reported outcomes, and to quantify complications.

**Methods:**

A PROSPERO-registered systematic review (CRD420261483110) was conducted following PRISMA 2020 guidelines. Five databases were searched to 15 July 2026 for children aged 0-18 with neurological impairment undergoing gastrostomy. Duplicate screening, data extraction, and risk-of-bias appraisal (RoB 2, ROBINS-I, JBI, CASP) were performed. Heterogeneity precluded meta-analysis; narrative synthesis prioritized study design and methodological quality over direction-of-effect vote counting.

**Results:**

From 5,503 records, 120 studies (≈117 cohorts, 30 countries) met the criteria. The evidence was predominantly observational, and most comparative studies carried a serious risk of bias. Nutritional status improved in the majority of 56 studies, though earlier placement's association with more robust catch-up growth does not establish causality. Among 56 respiratory studies, smaller cohorts reported fewer infections, but the largest propensity-matched and population-linked datasets showed no reduction, and in some analyses an increase, in pneumonia hospitalisations. Minor complications were common (20%–95%). Major complications clustered at 0%–3% without concurrent antireflux surgery, but rose several-fold when fundoplication or jejunal extensions were added. Reflux outcomes (59 studies) depended on preoperative status; it is hypothesized from limited physiological data that gastrostomy may increase de novo reflux risk in previously reflux-free children, though <10% required subsequent antireflux surgery. All-cause mortality (67 studies) was high but disease-related; procedure-attributable death was rare, and 30-day mortality was 0%–3% outside the most fragile groups. Quality-of-life data (21 studies) showed favourable caregiver outcomes in 14/17 studies, but the child's quality of life has been measured properly only once and was unchanged. Only 11 studies addressed neurodevelopment; the single study using a standardised instrument found cognitive recovery unrelated to the feeding route.

**Discussion:**

Gastrostomy is consistently associated with improved weight-based nutritional status and carries low serious surgical harm when performed without concurrent antireflux surgery. It does not eliminate aspiration risk and is hypothesized to increase de novo reflux risk in previously reflux-free children. Caregiver quality of life improves on validated measurement; however, the available evidence is insufficient to determine whether gastrostomy has a favourable, unfavourable, or neutral effect on a child's neurodevelopment.

**Systematic Review Registration:**

PROSPERO: https://www.crd.york.ac.uk/PROSPERO/view/CRD420261483110.

## 1 Introduction

Children with severe neurological impairment carry a high burden of oropharyngeal dysphagia, and that feeding difficulty has consequences on their growth, organ development, and family life (1). Conditions such as cerebral palsy and hypoxic-ischemic encephalopathy (HIE) disrupt the coordinated swallow, so oral intake becomes slow, unsafe or insufficient. A frequent consequence is malnutrition, where roughly one-third of neurologically impaired children referred for enteral support are already malnourished at presentation (2). Disruption of coordinated swallow can also lead to aspiration, a leading cause of death in children with co-existing gastro-esophageal reflux disease (GERD) (3). The nutritional deficits extend beyond physiological development, as childhood malnutrition impairs neurodevelopment, cognition and academic achievement as shown in cohorts followed into adulthood (4, 5). For a child whose brain is already injured, undernutrition therefore threatens whatever developmental reserve remains.

Gastrostomy feeding answers the nutritional problem directly, and the evidence for that narrow endpoint is consistent. Weight-for-age and BMI *z*-scores rise in the months after tube placement across single-centre and multicentre cohorts, regardless of the underlying diagnosis (2, 6, 7). Timing of gastrostomy placement impacts this growth response. Children who receive a gastrostomy earlier, before prolonged undernutrition, show faster catch-up and are more likely to normalise their nutritional status (6, 8). Additional benefits reported by caregivers describe easier and shorter feeds, better general health, and improved family life after placement, with satisfaction near 90% in population-based follow-up (9, 10).

Respiratory outcomes remain less settled. One prospective cohort found that chest infections requiring antibiotics and hospital admissions for chest infection both fell after gastrostomy, arguing against the fear that tube feeding worsens aspiration (11). Conversely, a large propensity-matched analysis linked gastrostomy placement to higher subsequent pneumonia hospitalisation, a signal the authors attributed to confounding by indication rather than to harm (12). Although serious complications are uncommon, placement itself carries a risk of granulation tissue, stomal infection, tube displacement, and occasional revision (10, 13). Health-related quality of life for the child is harder to interpret, because neurological impairment predicts lower scores whatever the feeding route (14).

Despite this body of observational work, the evidence base lacks methodological quality and breadth. Sleigh and Brocklehurst's systematic review found no randomized controlled trials and no controlled cohorts beyond a single comparison, relying instead on case series with risk of bias, and concluded that the true magnitude of both benefit and surgical risk could not be quantified (15). The subsequent Cochrane update identified no studies meeting inclusion criteria at all, concluding that considerable uncertainty about the effects of gastrostomy in children with cerebral palsy remains and that no reliable evidence base exists to guide families through this decision (16). Both reviews closed more than a decade ago, and both were restricted to cerebral palsy. Controlled comparisons have appeared since, but they contrast feeding routes over short follow-up windows, and the most recent Cochrane review examined how reflux should be managed alongside gastrostomy rather than what the operation does to a child's development. Nutritional and caregiver-reported outcomes have been examined repeatedly, but neurodevelopmental outcomes such as cognition, gross motor function, communication, and school participation have not been synthesised (4, 5, 17). This systematic review explores whether gastrostomy tube placement, compared with continued oral or nasogastric feeding, improves neurodevelopmental outcomes, nutritional status, respiratory health, and caregiver quality of life in children with neurological impairment and feeding difficulties.

## 2 Methods

This systematic review was conducted in accordance with the Preferred Reporting Items for Systematic Reviews and Meta-Analyses (PRISMA) 2020 guidelines. The review protocol was registered with PROSPERO (registration number: CRD420261483110) (18, 19).

### 2.1 Study design

This is a systematic review of quantitative and mixed-methods primary studies, without meta-analysis. It is reported in accordance with the Preferred Reporting Items for Systematic Reviews and Meta-Analyses (PRISMA) 2020 statement (18). The review question was framed using the PICOS structure set out in sections 2.2 to 2.5. No meta-analysis was undertaken, for the reasons given in section 2.10.

### 2.2 Participants

Eligible participants were children and adolescents aged 0 to 18 years with neurological impairment, defined as a static or progressive disorder of the central or peripheral nervous system associated with feeding difficulty. Qualifying diagnoses included all conditions of neurological impairment.

### 2.3 Interventions

The intervention of interest was placement of a gastrostomy for enteral feeding, by any technique and using any device. This included percutaneous endoscopic gastrostomy, laparoscopic and open surgical gastrostomy, radiologically inserted gastrostomy, low-profile balloon and non-balloon button devices, and gastrojejunal tubes placed through an existing or new gastrostomy tract. Studies in which a fundoplication or other antireflux procedure was performed concurrently with, or subsequent to, gastrostomy were eligible, and the presence, timing and type of any antireflux procedure was extracted for every study so that its contribution to the outcome could be examined separately.

### 2.4 Comparators

Three comparator structures were eligible: parallel comparison against children continuing oral or nasogastric feeding; within-subject comparison against the child's own pre-gastrostomy status, whether analysed as a simple before-and-after contrast or through a self-controlled or exposure-crossover design; and comparison between gastrostomy techniques, devices, or the presence or absence of a concurrent antireflux procedure. Single-arm descriptive studies without any comparator were eligible only for the complication, mortality and cross-sectional status outcomes, where they contribute rate data, and their contribution to before-and-after outcome domains was classified as not assessable.

### 2.5 Outcomes

Outcomes were specified across seven domains before data extraction began. The primary outcome domain was neurodevelopmental function, comprising cognition, gross and fine motor function, communication, adaptive behaviour, school participation and seizure control, measured by any standardised or non-standardised instrument. Secondary outcome domains were: nutritional status, including weight-, height-, BMI- and weight-for-height *z*-scores, anthropometry, catch-up growth, micronutrient status and excess adiposity; respiratory outcomes, including chest infection frequency before and after gastrostomy, respiratory and aspiration-related hospitalisation, and discrete aspiration events; minor complications, comprising peristomal infection, granulation tissue, leakage, dislodgement, obstruction and site pain; major complications, comprising buried bumper syndrome, colonic or other visceral injury, gastrocolic fistula, peritonitis, major haemorrhage, unplanned reoperation and procedure-related death; gastro-oesophageal reflux outcomes, including new or worsened reflux and subsequent fundoplication; and quality of life and caregiver-reported outcomes, including validated child and caregiver instruments, feeding time, medication delivery reliability and caregiver burden. All-cause and cause-specific mortality was extracted for every study.

### 2.6 Search strategy

The following search strategy was applied across all databases, combining four concept blocks with the Boolean operator AND and terms within each block with OR:

(“cerebral palsy” OR “neurological impairment” OR “neurologic impairment” OR “neurologically impaired” OR “neurodevelopmental disorder” OR “developmental disability” OR “intellectual disability” OR “neurological disability” OR “neurodegenerative” OR “Batten disease” OR “epilepsy” OR “epileptic encephalopathy” OR “developmental encephalopathy” OR “seizure disorder” OR “hypoxic ischemic encephalopathy” OR “HIE” OR “traumatic brain injury” OR “TBI” OR “spinal muscular atrophy” OR “SMA”) AND (“gastrostomy” OR “gastrostomy tube” OR “G-tube” OR “PEG” OR “percutaneous endoscopic gastrostomy” OR “gastrojejunostomy” OR “G-J tube” OR “enteral feeding” OR “tube feeding” OR “gastrostomy placement” OR “gastrostomy insertion” OR “button gastrostomy” OR “low-profile gastrostomy” OR “radiologically inserted gastrostomy” OR “RIG” OR “laparoscopic gastrostomy” OR “surgical gastrostomy” OR “open gastrostomy”) AND (“child” OR “infant” OR “adolescent” OR “pediatric” OR “paediatric” OR “children”) AND (“developmental outcome” OR “cognitive development” OR “motor function” OR “GMFCS” OR “gross motor function” OR “nutritional status” OR “weight gain” OR “growth” OR “malnutrition” OR “anthropometric” OR “BMI” OR “body mass index” OR “height velocity” OR “weight velocity” OR “respiratory” OR “pneumonia” OR “aspiration” OR “pulmonary” OR “hospitalization” OR “quality of life” OR “QOL” OR “HRQoL” OR “health-related quality of life” OR “caregiver burden” OR “family experience” OR “parent satisfaction” OR “gastroesophageal reflux” OR “GERD” OR “vomiting” OR “fundoplication” OR “complication” OR “adverse event” OR “infection” OR “granulation tissue” OR “survival” OR “mortality” OR “nasogastric” OR “NG tube” OR “oral feeding” OR “feeding time” OR “feeding duration” OR “sleep quality” OR “school attendance”).

No language restriction was applied at the search stage; non-English publications were excluded at screening.

### 2.7 Data sources

The following sources were searched from inception to 15 July 2026: PubMed, Embase, Scopus, CENTRAL and ClinicalTrials.gov. The reference lists of all included studies and of the two previous systematic reviews in this field were hand-searched for additional eligible records. Conference abstracts were eligible where sufficient methodological and outcome detail could be extracted.

### 2.8 Study selection & data extraction

Records retrieved from all sources were imported into Covidence (Veritas Health Innovation, Melbourne, Australia), which removed duplicates automatically. Screening was carried out by two independent reviewer teams working in parallel, each team comprising two reviewers (MD and HJ; MS and MA). Each team screened every record independently at title and abstract, and the same two-team structure was then repeated in full for full-text screening, so that every record and every full text was assessed twice. Conflicts arising at either stage, whether within a team or between the two teams, were resolved by the lead reviewer (MKI). No automation tool was used to make inclusion decisions.

Data were extracted onto a standardised extraction form developed for this review and piloted on a sample of studies before full extraction. The form captured bibliographic and design characteristics (author, year, country, design, single- or multi-centre, study period, follow-up duration, comparator); population characteristics (total enrolled, number with neurological impairment, primary neurological diagnosis and its distribution, proportion with cerebral palsy, age at gastrostomy, GMFCS level, baseline feeding method, aspiration history); intervention characteristics (gastrostomy type, presence and timing of any concurrent fundoplication); and every outcome listed under Outcomes, together with a free-text field for extractor notes and ambiguities and a field flagging suspected cohort overlap. Extraction was distributed across five reviewers, and the completed forms were checked centrally by the lead reviewer for internal consistency, unit errors and misassigned outcomes before synthesis.

Where the same cohort appeared in more than one publication, the reports were linked and counted once for any given outcome, with the most complete or most recent report used as the primary source and the companion publications identified in the footnotes of the relevant outcome table. Cohort overlap was identified from institution, recruitment period, sample size and author overlap.

Studies of mixed populations were eligible where children with neurological impairment formed the majority of the cohort, or where outcomes for the neurologically impaired subgroup were separable from those of other participants. Where a study reported only whole-cohort results for a mixed population, this is stated explicitly alongside the finding and the result was not treated as neurology-specific.

### 2.9 Risk of bias assessment

Risk of bias was assessed with the tool matched to each study design. The Cochrane Risk of Bias 2 (RoB 2) tool was applied to the randomised trial (20). The Risk Of Bias In Non-randomised Studies of Interventions (ROBINS-I) tool was applied to non-randomised comparative designs, including retrospective and prospective cohorts and chart reviews (21). The Joanna Briggs Institute (JBI) critical appraisal checklists were applied to cross-sectional studies, surveys and questionnaire-based research (22). The Critical Appraisal Skills Programme (CASP) qualitative checklist was applied to the qualitative interview studies (23). Assessments were performed independently in duplicate, with disagreements resolved by the external reviewer. Reporting bias across studies was not formally assessed with funnel plots or statistical tests, since these require pooled effect estimates; it is addressed narratively.

### 2.10 Data analysis

Clinical and methodological heterogeneity across the included studies was substantial. The studies differed in neurological diagnosis, impairment severity, age at placement, device and surgical technique, presence of a concurrent antireflux procedure, follow-up duration, and, most consequentially, in outcome definition: chest infection, aspiration, and minor complication were each operationalised differently across studies, and the same nutritional outcome was reported variously as an absolute weight, a percentage change, a *z*-score at a single time point, or a change in *z*-score. Meta-analysis was therefore not appropriate, and no pooled effect estimate is presented. This decision was taken after data extraction, once the extent of outcome-definition heterogeneity became apparent.

Findings were synthesised narratively within each outcome domain, using structured direction-of-effect vote counting. Each study contributing to a domain was assigned to one of five categories on the basis of the direction of the reported effect, irrespective of its magnitude or statistical significance: *favourable*, where the outcome improved after gastrostomy or was better than in the comparator group; *unfavourable*, where it deteriorated or was worse than in the comparator; *no difference*, where the study reported no significant change or between-group difference; *mixed*, where the study reported effects in opposing directions across measures or subgroups; and *not assessable*, where the outcome was mentioned but no baseline, denominator or numerator sufficient to judge direction was reported. Vote counts are presented as frequencies and are not interpreted as effect estimates, since a count of studies does not weight for sample size, study quality or precision. This approach follows Cochrane guidance on vote counting by direction of effect and is reported in line with the Synthesis Without Meta-analysis (SWiM) guideline. Studies were additionally classified according to whether their authors were, on the evidence and conclusions they presented, in favour of gastrostomy, against it, or mixed or neutral.

Within each domain, findings were then interpreted with explicit attention to whether the direction of effect tracked study design, on the reasoning that agreement between small uncontrolled cohorts and large comparator-controlled datasets is informative and disagreement between them is more informative still. Results are reported for each domain in the same order: the study-level table, the vote count, the highest-quality and largest contributing studies, the discordant studies, and a one-sentence synthesis. Subgroup observations were made where the included studies themselves reported them, specifically for age at placement, presence of a concurrent antireflux procedure, and device type; these were not pre-specified as formal subgroup analyses. No sensitivity analysis was possible in the absence of pooling.

Because of the extreme clinical and methodological heterogeneity of the observational data, including inconsistent outcome definitions, reliance on retrospective caregiver recall, and pervasive confounding by indication, a formal GRADE assessment of the certainty of evidence was deemed infeasible. Consequently, all clinical conclusions are presented as observational associations rather than established causal effects.

## 3 Results

### 3.1 PRISMA flow diagram

#### 3.1.1 Study selection & characteristics

The characteristics of all included studies are presented in Table 1. Data were extracted from 120 records.

**Table 1:** Characteristics of included studies.

Study	Country	Design	Sample size, N	Population (primary neurological diagnosis)	Age at gastrostomy	Follow-up duration	Gastrostomy type	Comparator
Zhang 2025 (27)	China	Randomised controlled trial (random number table, 42 vs. 42)—BUT journal labels the article “Observational Study”, Single-centre (Hunan Children's Hospital, Changsha)	*N* = 84; NI: 84 (100% NI—all traumatic brain injury with dysphagia)	Traumatic brain injury with persistent dysphagia (>4 weeks) 100% (84/84); TBI graded by Glasgow Coma Scale (control 8.96 ± 1.12, PEG 9.01 ± 1.15, *p* = .830). Cause of injury (control vs. PEG): falling collision 14 vs. 15, fall/self-harm 20 vs. 21, fall from height 8 vs. 6	PEG group mean 8.95 ± 1.88 years; NG control mean 8.68 ± 1.75 years (*p* = .164); eligibility restricted to ages 6–12 years	28 days post-treatment (no longer-term follow-up)	PEG (percutaneous endoscopic gastrostomy, traction/pull method, under general anaesthesia)	Indwelling nasogastric tube feeding (parallel, randomised; *n* = 42 PEG vs. *n* = 42 NG). Both groups received identical tube-feed formula and the same comprehensive swallowing rehabilitation programme
Grayeb et al., 2025 (39)	Israel	Retrospective cohort, Multi-center	*N* = 372; NI: 327 (88%)	NDD 88%, seizures 22% (overlapping)	Median 22.8mo (IQR: 11.5–47.2)	Median 64mo (IQR: 28–106)	PEG (excluded: primary surgical GT, primary GJ, jejunostomy)	None—single arm
Clayton 2024 (88)	United Kingdom	Retrospective observational medical-record review (2 centres) PLUS a separate cross-sectional UK-wide caregiver survey; the two datasets were analysed separately, Multi-centre—National Hospital for Neurology and Neurosurgery (adult cohort, *n* = 52) and Great Ormond Street Hospital (paediatric cohort, *n* = 72), London; survey circulated UK-wide by Dravet Syndrome UK	*N* = 124 in record cohort (of 140 identified; 1 adult and 15 children excluded) +65 survey respondents (of 73). GASTROSTOMY SUBGROUP: 21/124 (17%) record cohort, of which 10 are in the paediatric cohort; 26/65 (40%) survey cohort; NI: 124 (100% NI—all SCN1A-confirmed Dravet syndrome, a developmental and epileptic encephalopathy). Gastrostomy subgroup analysed here: *n* = 21 record cohort (*n* = 10 paediatric) and *n* = 26 survey	Dravet syndrome due to causal SCN1A variant 100% (124/124; alternative-gene or gene-negative cases excluded). Variant type in combined cohort: missense 50 (40%), stopgain 25 (20%), splice site 17 (14%), frameshift deletion 11 (9%), frameshift insertion 3 (2%), inframe indel 5 (4%), whole-gene deletion 1 (1%), unknown 12 (10%); null variants 40 (32%). Comorbid ASD 32/124 (26%) combined, 12/72 (17%) paediatric	PAEDIATRIC gastrostomy subgroup (*n* = 10): median 3.5 years (range 2.5–11, IQR: 2.9–5.3); all 10 (100%) inserted in childhood. Combined child + adult subgroup (*n* = 21): median 17 years (range 2.5–59, IQR: 3.5–27); only 11/21 (52%) inserted in childhood. Survey subgroup (*n* = 26): median 7 years (range 2–33); 22/26 (85%) inserted in childhood	Mean time from gastrostomy insertion to last follow-up/survey: paediatric cohort 3.8 years (SD 3.1, range 0–9); adult cohort 3.4 years (SD 3.4, range 0–11); survey cohort 6.1 years (SD 3.6, range 0.5–15). No combined-cohort figure given	NR—gastrostomy type and insertion technique (endoscopic vs. surgical vs. radiological, PEG vs. button) not reported for any cohort	Non-gastrostomy individuals within the same cohorts [record cohort: 97/124 (78%) no gastrostomy, 6/124 (5%) being considered; survey: 39/65 (60%) no gastrostomy]—used only for risk-factor comparisons. Post-gastrostomy outcomes themselves are single-arm/before-after with no comparator
Dipasquale 2023 (8)	Italy	Retrospective cohort, Single-centre	*N* = 50; NI: 50 (100)	genetic syndrome (*n* = 29, 58%), hypoxic-ischemic encephalopathy (*n* = 17, 34%), metabolic syndrome (*n* = 4, 8%)	mean: 4.65 years; range: 3 months–16 years	N/A	PEG 47 (94) PEG-J 3 (6)	None—single arm
Williams 2023 (102)	USA	Retrospective cohort, Multi-center	*N* = 6,118 total children underwent GT placement. Of those, 14,965 underwent isolated GT placement (no concurrent fundoplication) and formed the main analysis cohort; NI: 4,092 total NI children. (3,712 had isolated GT, and 380 had concurrent GT + fundoplication)	Not broken down by specific diagnosis. Authors used ICD codes combining cerebral palsy and degenerative neurologic diseases	For NI children with isolated GT: Median age 3 years (Interquartile Range: 1–8 years). For NI children who had a concurrent fundoplication: Median age 1 year	Median 3.78 years (evaluated up to 5 years)	NR	Children without neurologic impairment
Marpole, 2022 (118)	Australia	Retrospective cohort study, Single-centre	*N* = 221 (100%)	Cerebral Palsy	Mean 2.8, range 0–9 years	—	Gastrostomy (68% inserted by gastroenterology, 22% by paediatric surgery); 24% of gastrostomy patients also had a gastrojejunal (GJ) tube	None
Masui 2022 (119)	Japan	Retrospective cohort, Single-center	*N* = 35; NI: 35	—	Median 5.2 years	1 year	Laparascopic-assisted gastrostomy (LAG)	Pre-gastrostomy baseline (before/after)
Dreznik, 2022 (80)	Israel	Retrospective cohort study, Single-centre (Schneider Children's Medical Center of Israel, Petah Tiqva, Israel)	*N* = 345 patients; NI: 158 patients (45.7%)	Complications of prematurity (20%), asphyxia (18%), genetic neurological diseases resulting in refractory seizures (16.5%), global developmental delay with unknown aetiology (10%), irreversible brain damage due to other reasons (35.5%)	Mean age at gastrostomy was 3.77 years (±4.75 SD overall; NI mean 3.96 ± 4.65 years)	Median follow-up of 5 years (range 1–10 years)	Surgical gastrostomy (SG) according to Stamm technique or percutaneous endoscopic gastrostomy (PEG)	NI patients with vs. without fundoplication (subgroup analyses by refractory seizures)
Jadi, 2023 (71)	USA	Retrospective chart review, Single-center	*N* = 63; NI: 63	Cerebral Palsy 100%	Median 2 years (Interquartile range 0–8 years), Range 0–17 years	At least 1-year post-placement (outcomes measured at 3, 6, and 12 months)	Laparoscopic in 59 (95.2%), Open in 3 (4.8%), Missing data for 1. (Percutaneous was 0%)	Baseline compared to 3, 6, and 12 months post-gastrostomy placement
Civan, 2021 (120)	Turkey	Retrospective observational, Single-center	*N* = 41; NI: 41	Cerebral Palsy 100%	—	—	Technique: PEG	—
Guillén Redondo et al., 2021 (82)	Spain	Retrospective cohort, Single	*N* = 207; NI: 73.9% (153/207)	Neurological 73.9%—not specified	Age at gastrostomy Median 2 years (IQR: 1–8)	Mean 40.6 months; median 27 months (range 0.25–146)	Endoscopic PEG: 76% (158/207); Stamm (surgical): 24% (49/207)	Group 1A: gastrostomy alone (pre-existing GER) vs. Group 1B: gastrostomy + concurrent anti-reflux technique (pre-existing GER); Group 2: no pre-existing GER (gastrostomy alone)
Ulman 2021 (101)	Turkey	Retrospective cohort, Single-center	*N* = 109; NI: 100% (109)	Diagnoses included cerebral palsy, hypoxic ischemic encephalopathy, metabolic disorders, spinal muscular atrophy, mucopolysaccharidosis, hydrocephaly, West syndrome, Chediak Higashi syndrome, cardiofaciocutaneous syndrome, viral encephalopathy, and muscular dystrophy. Specific percentages for each were not reported.	Routine Screening group: median 61 months (range 6–204 months); Selective Screening group: median 60 months (range 6–212 months)	Media*n* > 2 years (Routine Screening period median: 26 months; Selective Screening period median: 28 months)	Laparoscopic	Routine preoperative GER screening vs. Symptom-selective preoperative GER screening
Lin, 2021 (121)	USA	Retrospective cohort study, Multi-centre/population-based (California Children's Services database)	*N* = 2,490 children (matched cohort); NI: 2,490 (100% have neurologic impairment and ≥1 pneumonia hospitalization)	Static neurologic impairment (35% total cohort), Progressive neurologic impairment (11% total cohort)	Mean age 7.36 years (SD 6.80; range 0–21 years)	Up to 180 days (and sensitivity analyses up to 365/548 days) post-discharge from index pneumonia hospitalization	Gastrostomy tube (G-tube)	Children with new G-tubes vs. children never receiving G-tubes (1:2 propensity score matched)
Sakamoto 2021 (69)	Japan	Retrospective cohort, Single-center	*N* = 89, 82 were included in the final short- and long-term complication analysis (2 died from underlying diseases, 5 were lost to follow-up); NI: 1	Of the 82 patients analyzed: "Cerebral damage in the neonatal period or infancy" 75.6% (*n* = 62). Other diagnoses included genetic or chromosomal anomaly 12.2% (*n* = 10), adrenoleukodystrophy 6.1% (*n* = 5), metabolic disorder 4.9% (*n* = 4), and mitochondrial disease 1.2% (*n* = 1)	Median 11.5 years	Categorized into short-term ( < 6 months) and long-term (>2 years). The median long-term follow-up was 1,770 days (interquartile range: 1,088–1,979 days)	VAG/LAP	—
Wadman 2021 (99)	United Kingdom, England & Italy	Retrospective chart review, Multi-center	*N* = 146; NI: 1	Spinal Muscular Atrophy (SMA) type 2 (100%). Broken down by subtype: Type 2a (83%) and Type 2b (17%)	Median 8 years (range 2.8–18.7 years)	Overall median 4 years (range 0–16). UK cohort median: 6 years; Italian cohort median: 3 years	NR	Pre-gastrostomy baseline (before/after)
Zambaiti 2021 (100)	Italy	Retrospective cohort, Single-center	*N* = 145; NI: 1	erebral palsy 29% (*n* = 42), Genetics 28% (*n* = 40), Metabolic disorders 17% (*n* = 24), Encephalopathy due to epilepsy 11% (*n* = 16), Acquired (post-trauma/infection) 6% (*n* = 9), Miscellanea 6% (*n* = 9), Mitochondrial disorders 3% (*n* = 5)	Median 3.4 years, range 9 months to 18 years	Median 4 years (range 1–12 years)	For the 127 patients who had initial gastrostomy alone: 91% PEG (Gauderer–Ponsky) and 9% laparoscopic-assisted PEG or Stamm. For the 18 synchronous fundoplication patients: 13 laparoscopic, 5 open	Gastrostomy only (GO) compared to Gastrostomy with fundoplication
Demirbaş, 2021 (44)	Turkey	Prospective study, Single-centre (Ondokuz Mayis University Medical Faculty, Samsun, Turkey)	*N* = 20 patients; NI: 20 (100% have neurological impairment)	Severe Class IV neurological deficiency (diverse etiologies including epilepsy, hydrocephalus, Rett syndrome, etc.)	Median age was 5.60 years (range 14 months–14.7 years)	Mean of 16.5 months (6–24 months) post-gastrostomy	Percutaneous endoscopic gastrostomy (PEG) for Group 1; laparoscopic gastrostomy with laparoscopic Nissen fundoplication (LNF) for Group 2)	Gastrostomy alone (Group 1) vs. Gastrostomy + laparoscopic Nissen fundoplication (Group 2)
Chae-ri Suh, 2020 (7)	South Korea	Retrospective cohort study, Single-centre	*N* = 18 (30 recruited, 12 excluded)	Spasticity conditions (77.8% total: Hypoxic ischemic encephalopathy 11.1%, Cerebral palsy 22.2%, Schizencephaly 5.6%, Alpert syndrome 5.6%, Lennox-Gastaut syndrome 16.7%, Devic syndrome 11.1%, Lissencephaly 5.6%); Hypotonic conditions (22.2% total: Cerebral palsy 5.6%, Rett syndrome 5.6%, Lennox-Gastaut syndrome 5.6%, Spinal muscular dystrophy 5.6%)	Median age at baseline was 132.4 months (IQR, 43.0–180.7 months)	Median intervention period of 50.4 months (IQR, 33.9–71.8 months); minimum follow-up criteria was ≤6 months	Percutaneous endoscopic gastrostomy (PEG) (16 pull-through, 1 push-through, 1 surgical re-insertion)	Subgroup comparisons (previous oral vs. NG tube feeding; hypertonic vs. hypotonic muscle tone)
Tavares de Sousa, 2020 (31)	Brazil	Cross-sectional study, Single-centre (Outpatient Clinic for Special Patients of Hospital de Clínicas de Uberlândia—Universidade Federal de Uberlândia, HC-UFU)	*N* = 28 patients; NI: 28 (100% have spastic quadriplegic cerebral palsy)	Spastic quadriplegic cerebral palsy (100%)	Mean participant age was 7.0 years (95% CI: 5.75–8.32, age ≤ 13 years); specific age at gastrostomy insertion not reported	Not applicable (cross-sectional study)	Gastrostomy used by 68% (*n* = 19) of the total cohort	None (correlational analysis between nutritional status and dietary/feeding variables)
Figueiredo 2020 (115)	Brazil	Cross-sectional, Single-center	*N* = 30 caregivers (not patients); NI: 1	tetraplegic cerebral palsy	not at gastrostomy: median 7 (4–19)	—	—	None—single arm
Glasson, 2020 (108)	Australia	Qualitative study using semistructured interviews and directed content analysis, Multi-centre/clinical networks (Perth Children's Hospital and Department of Communities, Western Australia)	*N* = 21 primary caregivers (representing 21 children); NI: 21 (100% have intellectual or developmental disability: 52% cerebral palsy, 33% genetic diagnosis, 14% global developmental delay/intellectual disability)	Cerebral palsy 52% (*n* = 11), Genetic diagnosis 33% (*n* = 7), Global developmental delay/intellectual disability 14% (*n* = 3)	Inserted at age 0–2 years in 48% (*n* = 10), 3–5 years in 29% (*n* = 6), 6–11 years in 19% (*n* = 4), unknown in 5% (*n* = 1)	Duration of using gastrostomy ranged from less than 2 years to 11 years (median 2.5 years)	Gastrostomy (percutaneous endoscopic gastrostomy/PEG mentioned in qualitative data)	None (qualitative interview study)
Jacoby 2020 (50)	Australia	Retrospective cohort, Multi-center	*N* = 673; NI: 1	nr	Median age for Cohort 1 was 3.4 years (IQR: 1.6–7.9). For Cohort 2, WA was 2.7 years (IQR: 1.3–4.6) and NSW was 1.5 years (IQR: 0.6–3.0)	median of 7.5 years for Cohort 1, 4.8 years for WA Cohort 2, and 6.7 years for NSW Cohort 2	nr	Self-controlled (1 year pre-gastrostomy vs. 1, 2, 3, 4, 5, and ≥6 years post-gastrostomy). It also compared Gastrostomy alone vs. Gastrostomy with Fundoplication
AlNamshan, 2019 (55)	Saudi Arabia	Retrospective review/cohort study, Single-centre (King Abdulaziz Medical City-Riyadh/King Abdullah Specialized Children Hospital, Riyadh, Saudi Arabia)	*N* = 178 children; NI: 178 (100% have neurological impairment)	Hypoxic-ischemic encephalopathy (HIE) 29% (51/178), Seizure disorder 18.5% (33/178), Syndromes 18.5% (33/178), Cerebral palsy 18% (32/178), Encephalopathy 6.2% (11/178), Congenital malformation 4.5% (8/178), Metabolic 3.4% (6/178), Traumatic brain injury 1.7% (3/178)	Mean age at fundoplication was 37.5 months (range 2–163 months); GT mean age was 44 months (range 0.25–206 months)	Median follow-up of 36 months (range 1–163 months) for fundoplication/44 months (range 0.25–206 months) for GT	Surgical gastrostomy (GT) (8% before, 90% with procedure, 2% after)	None (pre- vs. post-intervention comparison)
Franken 2020 (47)	Netherlands	Cross sectional, Single-center	*N* = 150; NI: 0.74	psychomotor retardation, epilepsy, microcephaly, spasticity, visual impairment, and/or hypotonia, but do not provide specific diagnosis percentages	Median 2.7 years (interquartile range 1.4–6.1 years), complete range 22 days to 16.7 years	Median 4.0 years (interquartile range 2.9–6.2 years)	Laparoscopic	The NI gastrostomy cohort was compared against neurologically normal (*N*N) children with a gastrostomy, as well as a reference group of healthy normal children
Franken 2019 (109)	Netherlands	Prospective longitudinal cohort, Single-center	*N* = 50; NI: 0.78	psychomotor retardation, epilepsy, spasticity, visual impairment, and/or hypotonia	Median age 3.4 years (interquartile range 1.4–5.6)	3 months post-operatively	Laparoscopic	Pre-operative vs. post-operative
Nelson, 2019 (57)	Canada (Ontario)	Population-based exposure-crossover study (retrospective cohort using linked administrative data), Multi-centre (24 Ontario hospitals)	*N* = 1,445 initially identified, 948 in final cohort (plus 17 early deaths included in survival analysis); NI: 948 (100% have neurologic impairment)	Cerebral palsy 49.3% (467/948), Central nervous system degeneration and diseases 19.9% (189/948), Brain and spinal cord malformations 18.8% (178/948), Muscular dystrophy, myopathies, and movement disorders 7.4% (70/948), Epilepsy 5.8% (55/948), Other central nervous system disorders 4.7% (45/948), Genetic and metabolic 3.9% (37/948), Malignancy 2.3% (22/948)	Aged 13 months to 17 years at tube placement (1–2 years: 38.6%, 3–5 years: 24.8%, >6 years: 36.6%)	Up to 5 years (median follow-up maximum 5 years, IQR: 3.6–5)	Gastrostomy tube [GT only: 57.4% [544/948]; Antireflux procedure/GJT or Nissen: 42.6% [404/948]]	Self-matching exposure-crossover design comparing 2 years before vs. 2 years after tube placement
Aumar, 2018 (63)	France	Prospective and retrospective observational study, Single-centre (CHU Lille, University of Lille, France)	*N* = 368 patients enrolled, 326 analyzed (42 lost to follow-up); NI: 208 patients (56% of analyzed cohort)	Neurologic impairment (56%)	Median age at PEG placement was 2.3 years (range, 1 month–25 years)	Median follow-up of 3.5 years (range, 2.0–13.5 years)	Percutaneous endoscopic gastrostomy (PEG) using standard pull technique	Neurologically impaired vs. typically developing children
Caselli 2017 (24)	Brazil	Cross-sectional, Single-centre	*N* = 25; NI: 25 (100% NI)	tetraspastic cerebral palsy 100%	N/A	N/A	N/A	Oral/NG feeding (parallel)
Figueroa 2017 (29)	Chile	Prospective cohort, Single-center	*N* = 81; NI: 81 (100%)	Cerebral palsy, 81 (100%)	with nutritional risk:8.0 (0 a 15) without nutritional risk: 4.5 (1 a 16)	1 year	—	None—single arm
Russell, 2017 (110)	USA	Mixed-methods study (online survey + qualitative semi-structured phone interviews), Multi-centre (US-based national non-profit organisations)	*N* = 36 survey respondents (6 interview participants); NI: 36 (100% have cerebral palsy)	Cerebral palsy (100%)	Mean age 3.4 years (SD 5.35, range birth–18 years) when enteral feeding was introduced	Children with GT for at least 1 month (mean child age 9.4 years, mean age at GT introduction 3.4 years)	Gastrostomy tube (GT)	None
Koca, 2015 (74)	Turkey	Retrospective study, Single-centre (Süleyman Demirel University Faculty of Medicine, Isparta, Turkey)	*N* = 34 children (47 PEG and PEG-related procedures); NI: 34 [100% have underlying feeding difficulties/dysphagia, predominantly neurological [64.7%] and metabolic [26.5%] diseases]	Cerebral palsy 32.4% (11/34), Hypoxic ischemic encephalopathy 17.6% (6/34), Hydrocephaly 8.8% (3/34), Metabolic diseases 26.5% (9/34), Pseudohypoaldosteronism 5.9% (2/34), Panhypopituitarism 2.9% (1/34), Congenital muscular dystrophy 2.9% (1/34), Tuberous sclerosis 2.9% (1/34)	Median age was 2.25 years (range 3 months–16 years)	Mean follow-up of 19.4 ± 10.3 months (range 6–42 months)	Standard PEG (Kimberly-Clark MIC PEG set) and button PEG (12 of 35% switched to button PEG at mean 8 ± 3.4 months)	Pre- vs. post-PEG comparison (subgroup analysis by age <2 vs. ≥2 years)
Kawahara 2014 (96)	Japan	Retrospective cohort, Single-center	*N* = 26; NI: 1	Cerebral palsy: 11 (42.3%) Chromosomal anomaly: 4 (15.4%) West syndrome: 3 (11.5%) Posttraumatic brain damage: 1 (3.8%) Advanced mental retardation: 1 (3.8%) Postherpes encephalitis: 1 (3.8%) Menkes disease: 1 (3.8%) Holoprosencephaly: 1 (3.8%) Metachromatic leukodystrophy: 1 (3.8%) Myoclonic encephalopathy: 1 (3.8%) Mitochondrial disease: 1 (3.8%)	Median 6 years, range 1–17 years	Clinical follow-up ranged from 10 months to 5 years and 6 months (median, 46 months)	Laparoscopic	Pre-operative vs. post-operative
Backman 2014 (122)	Sweden	Prospective cohort (consecutive series, before-after; labelled 'Clinical Study'), Single-centre (Department of Paediatric Surgery, Skane University Hospital, Lund)	*N* = 180; NI: Uncertain—61 (34%) have an unambiguous neurological diagnosis (cerebral palsy). Metabolic disease 37 (21%), “syndrome” 19 (10%) and part of the respiratory insufficiency group 15 (8%) may also be CNS-impaired but the paper does not say. Whole-cohort comorbidities: epilepsy 27 (17%), ventriculo-peritoneal shunt 9 (5%), mitochondrial disease 6 (4%), not mapped to diagnosis groups	Cerebral palsy 61 (34%), metabolic disease 37 (21%), cardiac malformation 30 (17%), “syndrome” 19 (10%), respiratory insufficiency 15 (8%), malignancy 11 (6%), GI malformations 7 (4%). Comorbidity across whole cohort: epilepsy 27 (17%), VP shunt 9 (5%), mitochondrial disease 6 (4%)	NR—mixed population, not separated [whole cohort: median age at operation 1 year, range 1 month–14 years]	6 months (prospective review at 1 month and 6 months; study endpoint 6 months)	Video-assisted gastrostomy (VAG)—laparoscopy-assisted button gastrostomy via continuous double U-stitch technique, all 180 children; gastroscopic confirmation of button position at end of procedure	Pre-gastrostomy baseline (before/after in the same children), plus between-diagnosis-group comparison (CP vs. cardiac, metabolic, GI, malignancy, respiratory, syndrome)
Franken 2015 (34)	The Netherlands	Retrospective cohort, Single-center	*N* = 300; NI: 0.75	psychomotor retardation, epilepsy, microcephaly, spasticity, visual impairment, and/or hypotonia. (Note: 9.7% of the total cohort had cystic fibrosis)	Median 2.66 years	Median 2.63 years	Laparoscopic	Pre-operative vs. post-operative
C. Barnhart, 2013 (104)	USA	Retrospective observational cohort study with propensity score matching, Multi-centre (42 children's hospitals in the United States via PHIS database)	*N* = 4,163 infants (1,027 propensity-matched pairs); NI: 4,163 (100% have neurological impairment and underwent GT placement in the NICU)	Static or progressive, central or peripheral neurological diagnoses associated with chronic functional or intellectual impairment	Infants admitted to the NICU within the first 90 days of life	1-year follow-up period post-discharge from NICU	Gastrostomy tube (GT) with or without fundoplication	Fundoplication and GT placement vs. GT placement alone
Åvitsland, 2013 (81)	Norway	Prospective cohort study, Single-centre (Oslo University Hospital, Norway)	*N* = 58 children; NI: 39 children (67%)	Neurologically impaired 67% (39/58), Congenital heart disease 17% (10/58), Other diagnoses 16% (9/58)	Median age 1.7 years (range, 0.5–14.7 years)	18 months postoperatively (assessed pre-op, 6 months, and 18 months)	Gastrostomy tube	Preoperative baseline (self-control)
Sharma 2012 (123)	UK	Retrospective cross-sectional study, Single-center	*N* = 41; NI: 1	Cerebral Palsy: 8 (19.5%) Chromosomal abnormality: 8 (19.5%) Neuro—Metabolic: 5 (12.2%) Other: 7 (17.1%) Expremature 25–28 weeks gestation: 3 (7.3%) Expremature 23–25 weeks gestation: 3 (7.3%) Global impairment—unknown cause: 2 (4.9%) Feeding difficulties—unknown cause: 2 (4.9%) Congenital brain malformation: 2 (4.9%)	Median (IQR) in early group: 11.5 (6–13) months; Median (IQR) in late group: 68 (38–82) months	12 months post-gastrostomy	nr	Early insertion (≤18 months, *n* = 24) vs. Late insertion (>18 months, *n* = 17). Pre- vs. post-operative data was also compared
Dahlseng 2012 (114)	6 European countries	Registry-based cross sectional	*N* = 245 CP patients	Cerebral Palsy	—	—	GT/PEG	None.
O'Loughlin, 2013 (43)	Australia	Retrospective cohort study with a cross-sectional questionnaire survey, Single-centre (Children's Hospital at Westmead and Westmead Hospital, Sydney, Australia)	*N* = 122 children; NI: 122 (100% have severe neurological impairment)	Spastic quadriplegic cerebral palsy 63% (77/122), Genetic/chromosomal 24% (30/122), Neurodegenerative 4% (5/122), Congenital cytomegalovirus 2% (2/122), Metabolic 1% (1/122), Unknown 6% (7/122)	Median age at fundoplication was 74 months (interquartile range 29–124)	Median duration from fundoplication to questionnaire was 77 months (43–89); overall follow-up included 122 patients (20 died, 13 lost to follow-up, 89 completed questionnaire)	Gastrostomy inserted in 117 of 122 (96%) patients (79% at fundoplication, 17% initially with later fundoplication, 4% fundoplication only without gastrostomy)	None
Mahant, 2011 (107)	Canada	Qualitative study using in-depth individual interviews, Single-centre (The Hospital for Sick Children, Toronto, Ontario, Canada)	*N* = 16 participants (parents); NI: 16 (100% have severe neurologic impairment)	Hypoxic ischemic encephalopathy 31.3% (5/16), Genetic/syndrome/metabolic 31.3% (5/16), Infectious 18.8% (3/16), Unknown 12.5% (2/16), Preterm IVH 6.3% (1/16)	Median age at intervention was 4.1 years (range, 0.1–14.5 years)	Minimum of 1 year post-procedure at time of interview	Fundoplication with gastrostomy (*n* = 8) or gastrojejunostomy tube (GJT) (*n* = 8)	Subgroup qualitative contrast between GJT group (*n* = 8) and fundoplication with gastrostomy group (*n* = 8)
Toporowska-Kowalska 2011 (95)	Poland	Prospective study, Single-centre	*N* = 15; NI: 1	Cerebral palsy: 10 (66.7%). Ceroidolipofuscinosis: 2 (13.3%). Encephalopathy following brain tumour surgery: 1 (6.7%). Encephalopathy following congenital toxoplasmosis: 1 (6.7%). Aicardi syndrome: 1 (6.7%)	Median age 122 months (10 years 2 months), interquartile range 77–178 months, range 4 to 16.7 years	6 to 8 months	PEG	Pre- versus post-operative within-subject comparison
Lefton-Greif 2011 (48)	USA	Retrospective review, Single-center	*N* = 28; NI: 1	Ataxia Telangiectasia (A-T): 28 (100%)	Median 12.6 years	4.11 years (median 3.58, range 0.28–11.16 years) after GT placement	gastrostomy tube	Pre- vs. post-operative within-subject comparison, and early mortality (≤30 days) vs. tolerated GT (>30 days survival)
Thomson 2011 (53)	UK	Prospective observational, Single-center	*N* = 10; NI: 1	Cerebral palsy: 9 (90%) Down's syndrome: 1 (10%)	Median 4.9 years (range 0.5–16.8 years) at pre-PEG evaluation	Impedance procedures took place 12–384 days (median 55 days) after PEG placement	PEG	Pre-PEG vs. Post-PEG within-subject comparison
Kawahara 2010 (97)	Japan	Retrospective cohort, Single-center	*N* = 54; NI: 1	pecific percentages are not listed, broadly described as “advanced neurologic impairment”. Specific syndromes mentioned in case outliers include alpha-thalassemia X-linked mental retardation, posttraumatic pituitary insufficiency, and Cockayne syndrome	Median 7 years (range 1–18 years)	Median 4 years (range 2–7 years) for the 48 patients who were followed up long-term	Laparoscopic	Pre- vs. post-operative within-subject comparison. The cohort was stratified by preoperative reflux index (RI): Group I (RI < 5.0%, *n* = 33) and Group II (RI ≥ 5.0%, *n* = 21)
Vernon-Roberts, 2010 (52)	UK	Longitudinal prospective feasibility study (Pre-post), Multi-centre	*N* = 14; NI: 1	Spastic quadriplegic cerebral palsy (SQCP) (100%)	Median 2y, range 10mo-11y	6 months	NR	Baseline (pre-gastrostomy)
Viswanath 2010 (61)	New Zealand	Retrospective cohort, Single Centre	*N* = 137; NI: 1	Cerebral palsy: 68 (49.6%), Developmental delay: 37 (27%) Seizures: 11 (8%) Encephalopathy: 9 (6.6%) Metabolic: 5 (3.6%) Chromosomal: 4 (2.9%) Microcephaly: 3 (2.2%)	Median age was 4.2 years (range 4 months to 15 years)	Median of 3.1 years (range 4 months to 9 years)	Gastrostomy type: Percutaneous endoscopic gastrostomy (PEG): 66 (48.2%). Laparoscopic gastrostomy: 44 (32.1%). Open gastrostomy: 27 (19.7%)	Historical comparison across three consecutive management protocols: Group A (1997–1999; *n* = 27): Concomitant open Nissen fundoplication performed in all children. Group B (2000–2002; *n* = 28): Concomitant laparoscopic fundoplication performed only in children with documented gastroesophageal reflux on preoperative 24 h pH study (18/28 underwent fundoplication). Group C (post-2002; *n* = 82): Gastrostomy alone performed; fundoplication was reserved selectively on follow-up only if GERD was persistent despite maximal
Sathesh-Kumar 2009 (70)	UK	Cohort study, Single centre	*N* = 76 children; NI: 0.76	Cerebral palsy: 25 (32.9%) Chromosomal abnormality: 13 (17.1%) Developmental delay: 4 (5.3%) Progressive neuro-axonal degeneration: 4 (5.3%) Brain malformations (microcephaly, lissencephaly): 4 (5.3%) Autistic spectrum disorders: 2 (2.6%) Muscular dystrophy and dystonia: 2 (2.6%) Epilepsy: 2 (2.6%) Spastic paraplegia (acquired): 2 (2.6%) Viral encephalopathy: 2 (2.6%) Encephalomalacia: 1 (1.3%) Metabolic abnormality: 1 (1.3%)	Median 3 years (range 6 months to 18 years)	Median 52 months (4.3 years); range 1 month to 12.6 years	Percutaneous endoscopic gastrostomy (PEG) using the standard pull-through (Gauderer–Ponsky) technique	Descriptive cohort
Day 2007 (25)	California	Retrospective cohort, Single-center	*N* = 24,920; NI: 24,920	CP	—	—	—	Oral/NG feeding (parallel)
Sullivan 2006 (32)	UK	Prospective cohort, Single centre	*N* = 40; NI: 1	Spastic quadriplegic cerebral palsy: 40 (100%)	Overall median 8 years 6 months (range 1y 4mo–18y 11mo). Specifically, median 9.0 years for the GT group and 8.0 years for the oral group	12 months	Gastrostomy tube (GT)	Gastrostomy-fed (*n* = 22) versus orally-fed (*n* = 18) children with CP
Petersen, 2006 (65)	USA	Descriptive, non-experimental (semi-structured interview), Multi-centre	*N* = 26; NI: 1	Cerebral palsy (100%): Athetoid (*n* = 10), Spastic (*n* = 8), Mixed (*n* = 6), Ataxic (*n* = 2)	NR (Mean child age at interview 4y 8mo; GT in place for mean 3y 1mo)	Mean 3y 1mo (range 3mo to 15y 9mo)	GT (not specified)	Retrospective caregiver recall
Craig 2006 (124)	UK	Prospective cohort, Single-centre	*N* = 76; NI: 76 (100%)	Cerebral palsy (*n* = 32), Syndromes (*n* = 25), Slowly progressive degenerative (*n* = 11), Unconfirmed diagnosis (*n* = 8)	median: 3 years 4 months, range 4mo–17y 5mo; only one child was aged more than 15 years	6 months post-op	51.3% PEG, 40.7% open, 8% laparoscopic	Pre-gastrostomy baseline (before/after)
Sullivan 2006 (11)	UK	longitudinal prospective, Multi Centre	*N* = 57; NI: 1	Spastic quadriplegic cerebral palsy: 43 (75.4%) Mixed cerebral palsy: 6 (10.5%) Hemiplegia: 3 (5.3%) Undiagnosed severe neurological impairment: 3 (5.3%) Ataxia: 1 (1.8%) Extrapyramidal disorder: 1 (1.8%)	Median 4.32 years (4 years 4 months); range 0.44 to 17.28 years	6 and 12 months	Percutaneous endoscopic gastrostomies (PEG) in 53 children, and open gastrostomies in 4 children	Pre- vs. post-operative within-subject comparison (baseline vs. 6 and 12 months).
Sullivan 2005 (33)	UK	longitudinal prospective, Multi Centre	*N* = 57; NI: 1	Spastic quadriplegic cerebral palsy: 43 (75.4%) Mixed cerebral palsy: 6 (10.5%) Hemiplegia: 3 (5.3%) Undiagnosed severe neurological impairment: 3 (5.3%) Ataxia: 1 (1.8%) Extrapyramidal disorder: 1 (1.8%)	Median 4.32 years (4 years 4 months); range 0.44 to 17.28 years	6 and 12 months	Percutaneous endoscopic gastrostomies (PEG) in 53 children, and open gastrostomies in 4 children	Pre- vs. post-operative within-subject comparison (baseline vs. 6 and 12 months).
Sullivan, 2004 (113)	UK	Prospective, longitudinal, Multi-centre	*N* = 57; NI: 1	Cerebral palsy: 54 (94.7%) consisting of: Spastic quadriplegic CP: 43 (75.4%). Hemiplegic CP: 3 (5.3%). Ataxic CP: 1 (1.8%). Mixed CP: 6 (10.5%). Extrapyramidal CP: 1 (1.8%) Undiagnosed severe neurological impairment: 3 (5.3%)	Median age was 4 years 4 months (range 5 months to 17 years 3 months)	post-gastrostomy insertion	Gastrostomy feeding tubes	Pre vs. post operation
Harrington et al., 2004 (103)	USA	Retrospective cohort, Single	*N* = 102; NI: 1	CP 71.6%; seizure disorder 48.0%; genetic defect 23.5%; anoxic brain injury 20.6%; prematurity <36wk 20.6%; congenital infection 11.8%; congenital hydrocephalus 5.9%; other (brain tumor ± hydrocephalus) 2.0%.	Mean 3.0 years (range 2 weeks–14 years); age distribution: <1y 32.4% (33/102), 1–4y 42.2% (43/102), 5–9y 16.7% (17/102), 10–14y 8.8% (9/102)	2 years post-discharge	PEG vs. surgical not distinguished	Gastrostomy alone (*n* = 37) vs. gastrostomy + concurrent antireflux procedure (*n* = 65); within gastrostomy-alone subgroup, seizure disorder present vs. absent
Wales et al., 2002 (84)	Canada	Retrospective cohort, Single	*N* = 111; NI: 1	Seizures: GJ 52.1% (25/48), FG 49.2% (31/63); CP: GJ 37.5% (18/48), FG 49.2% (31/63); developmental delay: GJ 20.8% (10/48), FG 25.4% (16/63); encephalopathy: GJ 20.8% (10/48), FG 19.0% (12/63); chromosomal: GJ 12.5% (6/48), FG 15.9% (10/63); microcephaly: GJ 16.7% (8/48), FG 11.1% (7/63); CNS tumor: GJ 10.4% (5/48), FG 0% (0/63, *P* < 0.05—significant difference between groups); metabolic: GJ 4.2% (2/48), FG 1.6% (1/63). Note: patients could have >1 diagnosis	Age at diagnosis: GJ mean 23.4mo (range 1–168); FG mean 29.3mo (range 1–192). Age at intervention: GJ mean 31.5mo; FG mean 41.6mo (*P* = 0.26, NS)	GJ: mean 3.11 years; FG: mean 5.71 years	FG: 100% received gastrostomy tube (Stamm technique) with fundoplication (76.2% complete 360° wrap, 23.8% partial wrap); pyloroplasty in 11.1% (7/63) for delayed gastric emptying. GJ: image-guided percutaneous gastrojejunal tube (Cook Pediatric Pigtail catheter, 8F/10F/12F), placed via interventional radiology	FG (open fundoplication + Stamm gastrostomy) vs. GJ (image-guided gastrojejunal tube, interventional radiology)
Nakao, 1999 (66)	NR	Prospective cohort (Pre-post), NR	*N* = 19 (16 followed prospectively); NI: 1	Neurological sequelae (100%)	NR	30 days to 11 months (median: 6.4 months)	NR	Baseline (pre-PEG)
Arnbjörnsson 1999 (125)	Sweden	Retrospective cohort, single-center	*N* = 98; NI: they say 18% but looking at the primary diagnosis list it's much closer to 75	unable to swallow: Multiple congen malformation 5 (2) Oropharyngeal dysmotility 7 (0) Neurological impairment 34 (18) Contusion cerebra/anoxia 4 (0) Cerebral tumour 4 (2)	range: 2mo to 19yr Average 3.2 yr	mean 39 months (range, 1 month to 5.5 years).	—	patients with lack of nutritional intake despite an ability to swallow
Smith, 1999 (9)	Canada	Population-based retrospective cohort + semi-structured caregiver interview + Vital Statistics linkage, Population-based (single tertiary center serving entire province; confirmed only 2 additional cases existed elsewhere, excluded due to unavailable data)	*N* = 61 total identified (1980–1998); of these, 45 had TF initiated 1990–1998 and were eligible for interview; 40/45 interviewed (4 refused, 1 lost to follow-up); NI: 100% (all CP)	CP subtypes (*n* = 40 interviewed): spastic 35/40 (87.5%); athetoid 3/40 (7.5%); mixed 2/40 (5%). Associated diagnoses: severe mental handicap 40/40 (100%); seizure disorder 27/40 (67%); contractures requiring surgery 25/40 (62%); cortical blindness 16/40 (39%); congenital abnormalities 7/40 (17%); non-verbal 37/40 (93%)	Mean 5 years (range 2 months–18 years); 42% received tube in first 2 years of life	Mean age at follow-up 8.5 years (range 1–20); Kaplan–Meier survival tracked to 10 years	Type breakdown (*n* = 40): gastrostomy tube 6; jejunostomy tube 3; gastrostomy + jejunostomy 6; gastrostomy button 23; jejunostomy button 2	None (single-arm, descriptive + survival analysis)
Brant, 1999 (13)	Brazil	Prospective cohort, Single-center	*N* = 20; NI: 1	CP 80% (16/20), myopathy 10% (2/20), brain trauma 10% (2/20)	Mean 6.5y	Mean 5.9 months	PEG	None—single arm
Tawfik, Dickson, Clarke & Thomas, 1997 (89)	UK	Retrospective cohort + structured caregiver questionnaire (35 items, statistically tested via one-tailed binomial test), Single	*N* = 29; NI: 1	CP (incl. 6 spastic quadriplegia) 9/29 (31%); neurological syndromes (Dandy-Walker, Cornelia de Lange, hypomelanosis of Ito, CHARGE, Joubert, Angelman, Cockayne) 7/29 (24%); postencephalitic brain damage 3/29 (10.3%); post-RTA head injuries 2/29 (6.9%); neurodegenerative disease 2/29 (6.9%); miscellaneous (nemaline rod myopathy, mitochondrial cytopathy, agenesis of corpus callosum, cerebral hypodevelopment, accidental drug overdose, translocation chromosomal abnormality) 6/29 (20.7%)	Median 4.5 years (range 0.4–20.6 years); majority (16/29) performed in first 5 years of life	1–5 years between op and assessment	Percutaneous endoscopic (for gastrostomy-alone group, (*n* = 13)	Gastrostomy alone (*n* = 13) vs. gastrostomy + fundoplication (*n* = 16)
Borowitz, 1997 (72)	USA	Prospective cohort (Pre-post), Single-centre	*N* = 19; NI: 1	Major neurologic impairment (100%)	Mean 33.6 months (SD 11.7, range 1.5–157 months)	Mean 20.7 months (range 7 to 33 months)	PEG	Baseline (Pre-PEG)
Cameron, 1995 (62)	Canada	Retrospective cohort, Single-centre	*N* = 63; NI: 1	Perinatal asphyxia/cerebral palsy (27% PEG, 33% SG), Cerebral dysgenesis/hydranencephaly (20% PEG, 36% SG), Chromosomal disorder/syndrome (13% PEG, 18% SG), CNS infection (13% PEG, 6% SG), Trauma/drowning (17% PEG, 0% SG), Metabolic (7% PEG, 3% SG), Other (3% PEG, 3% SG)	Average 3.5y (range 3mo-17y for PEG; 1mo-15y for SG)	Mean 23 months (minimum 12 months)	PEG (*n* = 30), Stamm gastrostomy (*n* = 33)	PEG vs. Stamm gastrostomy (SG)
Heine, 1995 (105)	Australia	Prospective before/after (42-item caregiver questionnaire +24 h oesophageal pH + endoscopy), pre/post PEG, Single-centre	*N* = 30 (18 M, 12 F); NI: 30 (100%)	Severe CP + intellectual disability 15/30 (50%); severe intellectual disability 8 (27%); severe CP normal intelligence 2 (7%); acquired NI 5 (17%: degenerative disorder, spinal cord compression, ruptured AVM, transverse myelitis, cerebro-costo-mandibular syndrome)	Mean 6 y (SD 5.7; range 3 mo–17 y)	Questionnaire 3–9 mo post-PEG; weight follow-up 3–12 mo; deaths tracked (mean time to death 9.7 mo, range 2–26 mo)	PEG (Gauderer push/pull); changed at 3–6 mo to silicone balloon catheter or low-profile device	Single-arm pre/post PEG (before vs. after). Baseline: 15 NG-fed, 15 oral-fed.
Sanders, 1990 (40)	USA	Retrospective cohort, before-after (3 groups by time since CNS insult), Single-centre	*N* = 51 (Grp1 *n* = 14, Grp2 *n* = 27, Grp3 *n* = 10); NI: 51/51 (100% CP)	Cerebral palsy 100% (spastic quadriplegia/diplegia; 4 hypotonic); cause pre/perinatal 34, head trauma 5, infection/hypoxia 12	Grp1 0.8y (3mo–3y), Grp2 4.1y (1.5–7.3y), Grp3 12.2y (8.5–15.75y); overall 3mo–15.5y	6 mo–5.5 y (mean 2.4 y)	Gastrostomy 47/51 (rest of cohort after 4 NG-only); 4 NG-only	Pre vs. post enteral feeding
Stringel, 1989 (91)	USA	Retrospective case series (3-year single-institution experience) with pre/post data, Single-centre	*N* = 90; NI: 90 (100%)	All 90 static encephalopathy. Aetiology: perinatal asphyxia 45; hydrocephalus 8; meningitis 5; microcephaly 5; head injury 5; congenital CMV 4; neonatal brain haemorrhage 3; prenatal infection 2; agenesis corpus callosum 2; neonatal hypoglycaemia 1; near-drowning 1; hypothyroidism 1; unknown 8	Mean 4 y (range newborn–17 y); 54 M, 36 F	Weight measured 3 mo post-op; parent questionnaire in 35 with >1 y follow-up; deaths tracked 2 mo–3 y post-op; '3-year follow-up' cited for GER recurrence	Stamm gastrostomy, de Pezzer catheter initially; at reassessment 40 button, 26 MIC tube, 22 de Pezzer (88/90 had a gastrostomy)	None (single-arm; pre/post surgery)
Wesley et al., 1981 (90)	USA	Retrospective case series, Single	*N* = 33; NI: 66.7% (22/33)—but ALL reported results are specific to this 22-patient subgroup	Causes of brain damage (*n* = 22): cerebral palsy 11/22 (50%); hypoxic insults (documented) 7/22 (31.8%); mental retardation as part of a specific syndrome 4/22 (18.2%)—specific syndromes listed in Table 1: Noonan (1), Cornelia de Lange (1), Down syndrome (1), Goldenhar (1), neurocutaneous disorder (1), achondroplastic dwarf (1)	Range 2 weeks–18 years; 11 patients < 2 years (average 1 year), 11 patients > 2 years (average 10.5 years)	Mean 11 months (range 2 months–2.5 years), excluding the 1 perioperative death (*n* = 21 analyzed for follow-up outcomes)	Open surgical technique—Malecot tube (16 or 18 French) placed via pursestring sutures in gastric fundus, performed concurrently with Nissen fundoplication + posterior gastropexy	None—single arm
Pinheiro et al., 2025 (42)	Brazil	Prospective cohort, before-after (T0/T1 pre, T2 post), Single-centre	*N* = 18 (23 operated; 5 excluded: 1 died, 1 no pre-data, 3 lost); NI: 18/18 (100% congenital Zika syndrome)	Congenital Zika syndrome 100% (microcephaly; all GMFCS V)	Mean 47.2 ± 18.7 mo (range 20–75)	∼1 y (T2 ≈ 376 ± 65 days post)	Gastrostomy (type NR); all exclusively gastrostomy-fed post	Pre vs. post gastrostomy
Tarek, 2024 (92)	Egypt	Prospective observational, Single-centre	*N* = 34 (17 peptide/17 casein); NI: 34/34 (100% severe NI, GMFCS 4–5)	Severe NI/CP (GMFCS 4–5); spastic quadriplegia grpA 82.4%, grpB 76.5%; flaccid tone minority	GrpA mean 4.1 ± 3y; grpB 4.7 ± 3.4y (range 2–14)	6 months (monthly monitoring)	PEG (pull technique, 20F)	Pre vs. post PEG (both arms); +peptide vs. casein formula
Salomone, 2022 (126)	Italy	Retrospective, before-after (6 mo + 1 y), Single-centre	*N* = 24; NI: 24/24 (100%)	NI, all GMFCS V (CP proportion NR here; 59% in A34 version)	Mean 9.7 ± 3.8 y	6 months (T1) + 1 year (T2)	PEG	Pre vs. post PEG
Jennuvat, 2023 (49)	Thailand	Cross-sectional descriptive + retrospective pre/post questionnaire, Single-centre	*N* = 39 completed (109 PEG performed; 60 followed up); NI: 39/39 (100% neurodevelopmental disability)	Spastic CP + epilepsy 32 (82.1%); Down syndrome 3; Trisomy 13 1; Pseudotrisomy 13 1; Aicardi 1; AADC deficiency 1	Median age at survey 103 mo (30–178); NG before PEG median 12 mo	Nutrition at 6 mo & 3 y; median PEG feeding 33 mo (2–106)	PEG (pull technique)	Pre vs. post PEG; vs. prior NG feeding
Işık & Akbulut, 2021 (68)	Turkey	Retrospective single-centre, Single-centre	*N* = 91; NI: 70/91 (76.9%)	Neurological disease (swallowing dysfunction) 70 (76.9%); metabolic 19 (20.9%); CF 2 (2.2%)	Median 79.5 mo (3 mo–18 y)	6 mo–5 y (mean 2.34 ± 2.45 y)	PEG (pull technique)	None (descriptive complications)
Cabello, 2021 (127)	Spain	Retrospective review (Pre-post), Single-centre	*N* = 87; NI: 73/84%	CNS abnormalities (89%), PNS disease (11%)	Median 30 months (range 74 days to 17 years)	NR (Median time to subsequent fundoplication was 236.6 days, range 36–786 days)	GTT (technique unspecified)	Baseline (Pre-GTT)
Noviello, 2021 (75)	Italy	Retrospective review, Title ’single-centre' but two surgical units (Naples + Ancona) listed	*N* = 148 (159 operated; 11 open-technique esophageal-atresia excluded); NI: 111/148 (75%)	NI 111 (75%); complex heart disease 18 (12%); metabolic 10 (6%); CF 4 (3%); muscle disease 4 (3%); chromosomopathy 1	Mean 4y overall (1 day–15y); NI + CF mean 7y (2–15y); others mean 6mo	Controls 1wk,1mo, then 6-monthly (total duration NR)	PEG 101 (68%), laparo-assisted 44 (29.7%), open/Stamm 3 (2%); GJ tube in 5	None (case series; by technique/age)
Franken, 2020 (14)	Netherlands	Prospective longitudinal cohort (before-after; pre vs. 3 mo post), Single-centre	*N* = 50 (symptom analysis); 25 completed pre + post MII-pH; NI: NI 36/50 (72%) overall; 20/25 (80%) in MII-pH group—SEPARABLE	Neurologic impairment 72%; CF 4%, undiagnosed FTT 4%, short bowel 4%, pulmonary 4%, cardiac 2%, metabolic 4%	Median 3.2y (IQR: 1.2–5.6) grp1; 4.7y (1.5–8.7) grp2	Median 3.9 mo (MII-pH); symptom f/u to Oct 2018	Laparoscopic gastrostomy (all)	Pre vs. post gastrostomy (self-controlled)
Wong, 2019 (86)	Australia	Retrospective population cohort/case series (CP register–ED linkage), Multi-centre	*N* = 183 ED presentations from 87 children (234 total FT presentations; CP cohort *n* = 2,183); NI: 100% CP	Cerebral palsy 100%	NR (age at PRESENTATION: 0–4y 39.9%, 5–9y 26.8%, 10–14y 26.2%, 15–19y 7.1%)	5-yr presentation window (retrospective)	G-tube 82.5% (151), GJ-tube 5.5% (10), other 10%; low-profile balloon 76%, initial PEG 12%	None (descriptive; g-tube vs. gj-tube contrasted)
Downs, 2019 (60)	Australia	Retrospective birth cohort study (Pre-post), Multi-centre (Population databases)	*N* = 673; NI: 1	Severe intellectual disability (100%)	NR	NR (Observed up to at least 4 years post-gastrostomy)	NR (Gastrostomy)	Baseline (the year before gastrostomy)
Downs, 2019 (51)	Australia	Retrospective cohort study, Multi-centre (Population databases—Western Australia)	*N* = 1,011 (severe ID cohort analyzed); NI: 1	Severe intellectual disability (100%)	NR	Up to 23 years	NR (Gastrostomy)	No gastrostomy
Martínez-Costa et al., 2019 (6)	Spain	Prospective, longitudinal, multicenter cohort, Multi-center	*N* = 98 recruited; 33 excluded (6 skeletal deformity affecting height measurement, 7 genetic disorder affecting growth, 20 missing anthropometric data); final *n* = 65; NI: 61.5% (40/65)	Neurological disease 61.5% (40/65), majority CP with GMFCS IV-V. Other cohort diagnoses (non-NI): cardio-respiratory disease 21.5% (14/65), inherited metabolic disease 9.3% (6/65), miscellaneous 7.7% (5/65: 2 swallowing disorder, 2 digestive disorder, 1 oncologic)	Overall cohort: 8 months–16.2 years (mean 3.1, SD 2.4). Subgroup: <18 months *n* = 24 (36.9%); >18.1 months *n* = 41 (63.1%)	12 months, assessed at baseline, 6 months, and 12 months	PEG 52/65 (80%: 50 pull-through technique, 2 gastropexy); open surgery 13/65 (20%). Pull-through PEG tubes replaced with low-profile button (Mic-Key) at 5–6 months	None—single arm
Kaur, 2019 (93)	India	Retrospective single-centre, Single-centre	*N* = 95; NI: ≈73/95 (≈77%)—borderline (see notes)	Acquired neurological disorders 34 (meningoencephalitis, TB meningitis, ADEM, intracranial neoplasms); CP 22; muscular/neuromuscular 17; polytrauma 12; metabolic 4; oropharyngeal 4; congenital heart 2	By band: <3mo 12 (mean 3.02 kg), 3–12mo 16, 1–5y 36, 5–10y 15, 10–15y 9, >15y 7	NR (16 lost to follow-up)	PEG (pull technique); 14/20/24 Fr by weight	None (descriptive)
Lertudomphonwanit et al., 2019 (59)	Thailand	Retrospective cohort, Single	*N* = 201; NI: 83.6% (168/201)	Cerebral palsy 20% (40/201); seizure disorders 15% (30/201); encephalopathy (various causes: anoxic/traumatic brain injury, post-viral encephalitis, CNS vasculitis, adrenoleukodystrophy) 14% (28/201); brain anomaly/post brain surgery 10% (20/201); muscular disorder/dystrophy 3.5% (7/201); globally developmental delay 4.9% (10/201).	Median 1.8 years (IQR: 0.6–5.8); 77/201 (38.3%) younger than 1 year; minimum age 1.5 months	1 year (nutritional outcome data available for 146/201 patients at this timepoint); complications tracked to 30 days post-procedure	PEG	None
Wadman, NR (99)	UK	Retrospective chart review (Pre-post), Single-centre	*N* = 47 (14 received gastrostomy); NI: 1	Spinal muscular atrophy (SMA) type II (100%)	NR (Cohort mean age is 10 years, range 2–19 years)	Mean 6 years (range 1–15 yrs) for total cohort	NR (Gastrostomy)	Baseline (pre-gastrostomy)
Ercoli 2017 (128)	Spain	Single-center	*N* = 224; NI: 81.4% (*n* = 22)	—	6.1 years (range 0.2–18)	—	PEG	None—single arm
Samuel, 2016 (45)	USA	Retrospective study (Pre-post), Single-centre	*N* = 32; NI: 32/100%	Rett Syndrome (100%)	NR (17 had G tube before 10 years of age; 13 had G tube placed after age 10)	3 months (for weight *z*-score)	Low profile (button) G tube in most	Baseline (pre-G tube)
Breton, 2016 (129)	Canada	Retrospective cohort (Pre-post), Single-centre	*N* = 259; NI: 164/63%	Neuromuscular disease (134/52% of total cohort)	Median 3.9 years (range 0.4–19.9 years)	Median 3.2 years (range 0–16 years)	NR	Baseline (time of PEG placement)
Machado 2016 (94)	NA	LONGTITUDONAL, SINGLE	*N* = 12; NI: 100	not specified	2 to 17 yrs-old (median 10yrs, 7F/5M)	5 months	PEG (standard pull technique)	none
Romano 2016 (130)	NA	Prospective, SINGLE	*N* = 56; NI: 100	not specified	aged 0.8–18 years)	6 months	Not specified	None
Frediani 2015 (111)	Italy	Survey, Single	*N* = 60	cerebral palsy 100%	NA	6 months	—	Oral fed
Lertudomphonwanit 2014 (131)	Thailand	Retrospective, Single	*N* = 133; NI: 100	cerebral palsy, intractable seizure disorders, congenital brain anomaly and encephalopathy	4.7 YEARs	nr	PEG	NONE
Kehar, 2014 (54)	India	Prospective cohort (Pre-post), Single-centre	*N* = 46; NI: ∼34/∼74%	Cerebral palsy (47.8%), Head injury (6.5%), Tubercular meningitis (4.3%), Intraventricular bleeding (4.3%), Medulloblastoma (4.3%), Rett syndrome (2.1%), GBS (2.1%), Duchenne muscular dystrophy (2.1%)	Median 6.75 years (range 5 weeks to 16 years)	Up to 12 months	PEG (standard pull technique)	Baseline (pre-PEG)
Toporowska-Kowalska 2013 (132)	Poland	Retrospective multicentre electronic questionnaire study (demographic and clinical data), Multi-center	*N* = 348; NI: 285/343 (81.9%) among subset with GER-diagnosis data available (denominator *n* = 343 of 348 total enrolled)—CNS impairment of various origins	CNS impairment of various origins 81.9% (285/343); specific diagnosis breakdown (e.g., CP subtype) NOT reported	5.78 ± 5.49 y	12 months (for nutritional status outcome specifically); GER/AR data collected retrospectively over the 2000–2010 decade	PEG 74.1%, SURGICAL 23%	Pre-gastrostomy baseline (before/after) for nutritional status only; no comparator for GER/AR outcomes
Gebora-Kowalska 2012 (41)	NR (not stated in abstract; author name suggests Poland—unconfirmed)	Prospective longitudinal cohort study (measurements at baseline, 6mo, 12mo), NR (not stated in abstract)	*N* = 21; NI: 21 (100% NI)	Neurological impairment 100% (specific subtype/diagnosis breakdown NOT reported)	Median age 9.67 (±4.72) years, range 0.6–16.2 years (age of participants at study entry, receiving HEN; not explicitly stated as age at gastrostomy)	12 months (measured at baseline, 6mo, 12mo post-PEG)	PEG (percutaneous endoscopic gastrostomy)	Pre-gastrostomy baseline (before/after)
Fascetti-Leon 2012 (76)	Italy	Prospective cohort, Multi-center	*N* = 239; NI: 189 (79%)	N/A	mean age was 6.05 ± 6.1 years (median 8.13, range 10 days–25 years)	24 months	PEG	Pre-gastrostomy baseline (before/after)
Vergara 2011 (26)	Not stated explicitly; published via Medigraphic (Mexican journal platform)—likely Mexico, unconfirmed	Prospective comparative cohort study (two treatment groups), NR (not stated in abstract)	*N* = 55; NI: 55 (100% CP)	Cerebral palsy 100% (*n* = 55). Etiology: neonatal hypoxia 41%, meningitis 27%, cranial trauma 9.1%	NR (not stated in abstract)	NR (not stated in abstract)	NR (not specified as PEG vs. open/surgical)	Gastrostomy + Nissen fundoplication (*n* = 29) vs. no surgical treatment (*n* = 26)
Chao, 2009 (46)	Hong Kong (China)	Retrospective cohort (Pre-post), Single-centre	*N* = 46 (39 completed review); NI: 1	Cerebral palsy from perinatal anoxia (33%), Hydrocephalus/cerebral atrophy (33%), Syndromic (26%)	Mean 6.8 years (range 4 months to 15 years)	Mean 25 months (range 7 months to 6 years)	Gastrostomy (concomitant with fundoplication)	Baseline (6 months pre-operation)
Saadah 2009 (98)	Saudi Arabia	Retrospective cohort, single-center	*N* = 25; NI: 25 (100%)	cerebral palsy 25 (100%)	mean age 6.0 ± 5.3 years, range 1–21	6 months	—	Pre-gastrostomy baseline (before/after)
Motil 2009 (36)	United States	Retrospective cohort, Single-center	*N* = 95; NI: 95 (100%)	rett syndrome 95 (100%)	mean 5.9 ± 3.8 years	4.5 3.9 years	—	Pre-gastrostomy baseline (before/after)
Vernon-Roberts 2009 (133)	UK	Retrospective cohort, single-center	*N* = 84; NI: 84 (100%)	—	—	minimum 1 year	—	None—single arm
Toporowska-Kowalska 2008 (134)	Poland	Prospective single-arm cohort, Single	*N* = 14; NI: 14 (100% NI)	Chronic disease of the central nervous system 100% (14/14); specific diagnoses NR	NR (age at gastrostomy); age at study 4–16.7 years (range only, no mean/median)	NR	PEG in 11; PEG/PEJ (gastrojejunostomy) in 1;	None—single arm (pre- vs. post-gastrostomy clinical status; MII-pH subgroups compared)
Vajsar 2006 (58)	Canada	Retrospective chart review (single-arm case series), Single-centre (“the Hospital for Sick Children”)	*N* = 12; NI: 12 (100% NI—all SMA type 1)	Spinal muscular atrophy type 1, 100% (12/12)	Median 6.1 months (range 2.2–14.5 months)	30 days post-insertion (by design—no longer-term follow-up)	Gastrostomy and/or gastrojejunostomy, placed by image-guided percutaneous technique. The split between G and GJ tubes is NR, so outcomes cannot be attributed to gastrostomy alone	None—single arm
Arnbjörnsson 2006 (67)	Sweden	Retrospective cohort, Single-center	*N* = 54; NI: 27 (50%)	Neurological disease	—	6 months post-op	video-assisted gastrostomy	Children with malignant disease
Seguy 2002 (37)	France	Retrospective cohort, single-center	*N* = 12; NI: 12 (100%)	DMD *n* = 3 CMD *n* = 2 SMA *n* = 3 CM *n* = 3 CP *n* = 1	range: 1 month to 25.5 years	1 year	PEG	—
Benoit 2001 (64)	Canada	Retrospective chart review of a consecutive case series, with between-diagnosis comparison of tube removal and Kaplan–Meier tube-retention analysis, Single-centre (tertiary care paediatric hospital, Hospital for Sick Children, Toronto)	*N* = 325 (CP subgroup 49; 203 had ≥12 months follow-up, of whom CP 36); NI: 49/325 (15%) cerebral palsy (definition includes birth asphyxia). A further 44 (14%) in the “other” group mixes CNS conditions (closed head injury, shaken baby syndrome, meningitis/encephalitis, near drowning, infantile encephalopathy, subdural haematoma) with non-CNS ones (myopathy, muscle atrophy, central hypoventilation, diabetic coma) and is NOT separable—so the true NI proportion lies somewhere between 15% and about 27%	Cerebral palsy (incl. birth asphyxia) 49 (15%); genetic and congenital disorders 220 (68%); cancer 12 (4%); other 44 (14%)	CP subgroup: median 7 months (range 1–36 months). [Whole cohort: median 6 months, range birth-36 months—eligibility capped insertion age at 36 months by design]	Variable. 203/325 (62%) had at least 12 months of follow-up after insertion; Kaplan–Meier retention plotted to 36 months. Age at tube removal median 19 months (range 3–58)	CP subgroup: gastrostomy 27/49 (55%) AND gastrojejunostomy 22/49 (45%)—radiologically inserted. All analyses pool G with G-J, so outcomes cannot be attributed to gastrostomy alone. [Whole cohort: G 199/323 (62%), G-J 124/323 (38%)]	Other diagnostic groups within the same cohort (genetic/congenital *n* = 220, cancer *n* = 12, other *n* = 44). No non-tube-fed comparator. Nutritional status also compared before insertion vs. at removal
Thorne 1998 (112)	Canada	Retrospective cohort, single center	*N* = 62; NI: 61 (100%)	spastic CP 61 (100%)	—	at least 6 months	tube *n* = 31, surface *n* = 31	None—single arm
Saleem 2023 (73)	Pakistan	Prospective cohort, single center	*N* = 40; NI: 40 (100%)	Cerebral palsy 32 (80%) neurodegenerative disorder 4 (10%) neuromuscular disorder 4 (10%)	mean 39.8 ± 20.2 months (Range 11–78 months)	6 months	—	Pre-gastrostomy baseline (before/after)
Wu 2026 (28)	United States	Retrospective cohort study (non-randomised: new gastrostomy vs. continued nasogastric tube at inpatient rehabilitation admission), Single-centre (paediatric inpatient rehabilitation unit, Spaulding Rehabilitation Hospital, Boston)	*N* = 74 (86 screened; 10 excluded as tube was for medication only on a regular diet, 2 excluded for an interval rehab admission elsewhere); NI: 74 (100% NI—all new neurologic impairment due to acquired brain injury)	Acquired brain injury 100% (74/74): traumatic brain injury 28 (38%), brain cancer 23 (31%), childhood stroke 15 (20%), non-traumatic acquired brain injury 8 (11%)—the latter comprising autoimmune encephalitis/encephalopathy 3, hypoxic-ischaemic brain injury 3, infectious encephalitis 2. 47 boys/27 girls	GT group mean 12.2 ± 5.0 years vs. NG group 9.0 ± 4.6 years (t = 2.86, *p* = .006—GT children were significantly OLDER). Whole cohort mean 10.7 ± 5.1 years; eligibility 3–21 years	Inpatient rehabilitation stay only: mean 43.2 ± 29.1 days from admission to discharge. No post-discharge follow-up. (Preceding acute hospital stay 36.7 ± 28.6 days)	Gastrostomy, newly placed during the preceding acute hospital admission (before rehab transfer). Tube type, route and technique NR. 3 children converted NG-to-GT during rehab and were analysed in the NG arm on initial admission status	Continued nasogastric tube feeding, parallel and NOT randomised. Group sizes in Results: NG 34 (46%) vs. new GT 40 (54%), chi-sq = 0.05, *p* > .4. NOTE the Abstract instead states NG 51%/GT 49%—the two do not agree
Stewart 2017 (85)	United Kingdom	Retrospective case-note review (case series with within-cohort risk-factor comparison; two-tailed Fisher's exact test), Single-centre (Evelina London Children's Hospital, London—affiliation from the PubMed record, not the abstract text)	*N* = 535 gastrostomy patients reviewed; 29 developed buried bumper syndrome; NI: NR—mixed population, not separated. Neurodevelopmental comorbidity is reported ONLY for the 29 BBS cases (26/29, 90%). The proportion of the 535-patient denominator with neurological impairment is never given, so no NI-specific rate can be calculated	NR—mixed population, not separated. Comorbidities are given only for the 29 BBS cases: neurodevelopmental 26/29 (90%), cardiorespiratory 14/29 (48%), genetic 16/29 (55%); 27/29 (93%) had two or more comorbidities. No specific neurological diagnoses are named, and no comorbidity data are given for the 506 patients without BBS	NR (age at gastrostomy). Age at BBS presentation median 8.6 years (range 1–18 years)	NR overall. Mean time from insertion to development of BBS 1,025 ± 634 days (about 2.8 years); no total cohort follow-up duration given, so 5.4% is a crude proportion rather than an incidence rate	PEG alone 475/535 (89%); PEG with jejunal extension (PEG-J) 60/535 (11%)	Within-cohort comparisons only: PEG alone (475) vs. PEG with jejunal extension/PEG-J (60), and first vs. second/subsequent tube. No non-gastrostomy comparator
Park 2011 (78)	South Korea	Retrospective Cohort, Single centre	*N* = 32; NI: 100	Specific breakdown by percentage is not provided, though the authors note patients in this population may be diagnosed with conditions like hypoxic brain damage, leukomalacia, infantile spasms, epilepsy, or cerebral palsy.	Mean 9.4 years (SD 4.5); range 2.4 to 17 years	Mean 12.2 months (SD 6.6)	PEG	Pre- vs. post-operative within-subject comparison
Downs 2014 (135)	Australia	Retrospective cohort, Multi-center	*N* = 229; NI: 229	Rett syndrome 100%	N/A	11 years	Not stated	Oral/NG feeding (parallel)
Ramelli 2007 (56)	UK	longitudinal cohort, Single centre	*N* = 31; NI: 100	Congenital muscular dystrophy (*n* = 15, 47%), structural congenital myopathies (*n* = 11, 34%), other neuromuscular disorders (*n* = 6, 19%)	Mean 7 years 4 months (range 3 months to 28 years)	12 months to 19 years (mean 5 years)	PEG	Pre- vs. post-operative within-subject comparison
Valletta 2004 (116)	UK	Longitudinal cohort study, Single centre	*N* = 11; NI: 1	—	—	Median 15 months (range 3–66).	PEG	—
Issacs, 1995 (136)	USA	Pre-post cohort (own control), Single-centre	*N* = 22; NI: 1	Severe neurologic impairments (100%)	Range 1 to 12 years	10 months to 4 years	Gastrostomy (unspecified)	Baseline (pre-surgical)
Puntis 2000 (77)	UK	Retrospective cohort, Single centre	*N* = 29 (27 underwent successful PEG placement; 2 required open gastrostomy due to anatomical issues); NI: 29 (100%)	Developmental delay with or without recognized syndrome: 12 (41.4%). Cerebral palsy: 8 (27.6%). Neurodegenerative disorder: 3 (10.3%). Neuromuscular disease: 3 (10.3%). Hypoxic or traumatic brain injury: 3 (10.3%)	Median 5.6 years (range 1.1 to 18.0)	Median 2.6 years (range 0.4 to 4.9)	PEG & 2 Children required open	Pre- vs. post-operative within-subject comparison
Khattak, 1998 (79)	UK	Retrospective case series (medical-records review), Single-centre	*N* = 120 patients (130 PEGs); NI: 52 (43%)	NI subgroup 52 (specific mix not broken out). Whole-cohort indications: inability to swallow 74 (NI 52, congenital malformations 10, oropharyngeal dysmotility 4, EB 3, other 5), inadequate calorie intake 30 (CF 14, CHD 6, chronic resp failure 3, other 8), special feeding 12, continuous enteral 4	Whole-cohort range 5 mo–22 y (mean 7 y); NI-specific age not given	Mean 26.6 mo (range 3 mo–6 y); long-term (>12 mo) follow-up in 80%	PEG (“pull” technique, Gauderer)	None (single-arm case series)
Langer, 1988 (83)	Canada	Retrospective cohort (FG alone vs. FG + ARP; stratified by pre-op GER status: group 1 GER + *n* = 44, group 2 GER− *n* = 54, group 3 no assessment *n* = 9), Single-centre	*N* = 107 (58 M, 49 F); NI: 107 (100%)	Cerebral palsy 51; familial syndromes 16; head injury 9; mental retardation 8; hydrocephalus 7; microcephaly 6; degenerative disorders 5; brain tumours 4; stroke 1 (*n* = 107)	Mean 4.3 y (range 1 mo–16 y)	Mean ∼20 mo (group 2 = 20.0 mo; group1 FG-alone 22.6 mo, FG + ARP 15.5 mo; overall range 1–66 mo)	Feeding gastrostomy—DePezzer 55, Mallecot 49, Foley 3; 12–14 Fr. Endoscopic in 38 of FG-alone; Stamm (open) in the other 33	FG alone (*n* = 71) vs. FG + antireflux procedure (*n* = 36)
Shapiro, 1986 (106)	USA	Retrospective before/after (pre/post-gastrostomy growth review), Single-centre	*N* = 19; NI: 19 (100%)	All 19 cerebral palsy, severely/profoundly impaired; all severely–profoundly mentally retarded; 11 seizure disorders; 2 genetic syndromes w/cleft palate; 3 slowly progressive neuro disease; 17/19 oral-motor dysfunction	Mean 60.4 mo (range 5–168 mo)—NB table “Age” is age at assessment	≥6 mo post-op; mean 23.1 mo (range 6–41 mo)	Not specified	Single-arm pre/post gastrostomy (weight/length before vs. after)
Wong, 2020 (87)	Australia	Retrospective propensity score matched cohort study, Population-based (procedures performed at a single tertiary children's hospital)	*N* = 1,011 in overall cohort, 252 in 2000–2009 cohort, 212 in matched cohort; NI: 100% (all patients had severe intellectual disability)	Severe intellectual disability (100%)	Not reported	Median 6 years 3 months (IQR: 3 years 7 months to 8 years 9 months)	Not reported	Propensity-matched individuals with severe intellectual disability who did not receive a gastrostomy
Arrowsmith, 2010 (38)	Australia	Prospective cohort study, Single-centre	*N* = 21; NI: 1	Spastic quadriplegic cerebral palsy (100%)	Median 8 years 5 months (IQR: 6 years 9 months to 11 years 10 months)	Median 19.4 months on gastrostomy feeds (IQR: 7.7–29.9 months)	Not reported	Healthy comparison children (data extracted from databases)
Catto-Smith, 2006 (35)	Australia	Retrospective cohort study, Single-centre	*N* = 98 (out of 173 total PEGs); NI: 1	Cerebral palsy (35%), Chromosomal/multiple congenital abnormalities (22%), Infection (15%), Metabolic disease (11%), Postnatal injury (5%), Others (11%)	Median 3.5 years (range 60 days to 20 years)	6–14 years	PEG	No comparator group
Finbrårten 2011 (30)	Norway	Registry-based cross-sectional study	*N* = Multi-centre (all Norwegian paediatric habilitation centres, via national CP registry); NI: 661	N/A (no comparator group; growth compared to Norwegian population growth charts)	0 (0%)	Born 1996–2003 (birth cohort); registry data collection period not stated	Not reported	N/A (cross-sectional)

Several included records derive from the same or an overlapping source population but are retained as separate rows because each reports different outcome domains for this review; they are not treated as independent samples in any synthesis. Studies drawn from the same cohort: (1) Kawahara 2010 and Kawahara 2014; (2) Sullivan 2004, Sullivan 2005, and Sullivan 2006 (Oxford Feeding Study); (3) the Clayton 2024 medical-record cohort (*n* = 124) and the Clayton 2024 caregiver-survey cohort (*n* = 65), which may include overlapping caregivers and are therefore not combined; and (4) Downs 2019, Wong 2019, Wong 2020, and Jacoby 2020, which draw on the same Western Australian population-linked data collections.

CP, cerebral palsy; GERD, gastro-oesophageal reflux disease; NI, neurological impairment; PEG, percutaneous endoscopic gastrostomy.

The evidence base is overwhelmingly observational. One randomised controlled trial was identified, and this trial is labelled by its own journal as an observational study despite describing random-number-table allocation, so its design classification is uncertain. The remaining studies comprised retrospective cohorts and chart reviews, prospective cohorts, retrospective case series, cross-sectional and registry-based studies, caregiver surveys, and three qualitative interview studies. Most cohorts were single-centre. The most common analytical structure by a wide margin was the single-arm before-and-after comparison, in which children served as their own controls; genuinely parallel comparisons against continued oral or nasogastric feeding were reported in only a small minority of studies, and the largest of these are administrative or population-linked datasets rather than clinical cohorts.

Studies originated from 30 countries, most frequently the United Kingdom (*n* = 20), the United States (*n* = 17), Australia (*n* = 9), Canada (*n* = 9) and Italy (*n* = 7), with smaller contributions from across Europe, the Middle East, East and South Asia and Latin America. Publication dates spanned more than four decades, from 1981 to 2026, and the older surgical case series report techniques, devices and perioperative care that differ materially from contemporary practice.

Cohort size ranged from 10 children to more than 24,000, the largest datasets being administrative or registry-linked. Age at gastrostomy varied widely both within and between studies, from the neonatal period to late adolescence, with most cohorts reporting a median age between one and eight years. Follow-up ranged from 28 days to a maximum of 19 years and was under one year in a substantial proportion of studies. Reporting of impairment severity was poor: GMFCS level was recorded in only a minority of cohorts, and many studies grouped participants under an undifferentiated heading of neurological impairment without specifying the underlying diagnosis or its distribution. Cerebral palsy was the single most common diagnosis where diagnosis was reported, but a substantial number of studies did not separate cerebral palsy from other causes of neurological impairment, which limits the precision with which findings can be attributed to any specific population.

### 3.2 Synthesized findings

Findings are presented by outcome domain. The direction-of-effect vote count for each domain is summarised in Table 2, and study-level data are given in Tables 3–6.

**Table 2:** Direction-of-effect vote count by outcome domain.

Outcome domain	Contributing studies	Favourable	Unfavourable	No difference	Mixed	Not assessable
Nutritional status	53	39	0	1	9	3
Respiratory outcomes	56	24	4	4	8	16
Minor complications	43	8	13	0	22	0
Major complications	60	30	16	0	10	4
Gastro-oesophageal reflux	60	19	15	3	15	8
Quality of life—child	16	13	0	0	1	2
Quality of life—caregiver	17	14	0	0	3	0
Mortality	69	41	7	1	14	6
Neurodevelopmental function	11	3	0	3	0	5

Counts are frequencies of studies and are not effect estimates; they are not weighted for sample size, precision or study quality, and should be read alongside the design-stratified interpretation in Sections 3.3, 4.1. Studies contributing to more than one domain are counted once in each.

**Table 3:** Nutritional outcomes after gastrostomy.

Author/Year	N (NI)	Design	Follow-up	Finding	Direction	Risk of bias
Caselli 2017 (24)	25 (25, 100% NI)	Cross-sectional	N/A	GT-fed children had greater arm circumference, triceps skinfold, and adipose arm area than orally-fed peers; higher protein/fibre intake in oral group, higher lipid intake in GT group; no difference on other measures.	Mixed (favourable on adiposity/anthropometry vs. oral comparator)	Moderate/High
Craig 2006 (124)	76 (76, 100%)	Prospective cohort	6 months post-op	Upper-arm circumference 16.04 → 17.16 cm; ≤2 SDS for weight 64% → 54% (≤3 SDS: 42% → 33%); 70% achieved catch-up growth (mean WAZ + 0.51). No change in micronutrient intake except small fall in vitamin C (reduced fruit juice).	Favourable (growth); NS (most micronutrients)	Moderate
Dipasquale 2023 (8)	50 (50, 100%)	Retrospective cohort	Single-arm; 3 & 6mo	WAZ and BMIZ rose progressively baseline → 3mo → 6mo (BMIZ: −1.35 → −0.1 → +1.0); MUAC change was NS. Older age at initiation associated with poorer 12mo status.	Favourable	Moderate
Day 2007 (25)	24,920 (100% NI)	Retrospective cohort	Not reported	Large administrative cohort: GT-fed children gained more weight than orally-fed peers across all age bands (largest gain ≈ 29%–30% at age 10); GT-fed children with severe CP grew taller than orally-fed peers with severe CP.	Favourable (vs. oral comparator)	Low
Figueroa 2017 (29)	81 (81, 100%)	Prospective cohort	1 year (cross-sectional)	Cross-sectional status at 1yr post-GT (BMIZ −1.86, HAZ −2.80, fat mass 105%); no baseline, so within-subject change not determinable.	Not assessable (no baseline comparison)	Moderate
Civan 2021 (120)	41 (1 NI reported)	Retrospective observational	Not reported	Significant gains in height, weight, triceps skinfold (*p* < 0.05); haemoglobin improved (*p* = 0.022); vitamin D, B12, folate, iron, zinc also monitored.	Favourable	Serious
Jadi 2023 (71)	63	Retrospective chart review	≥1 year (3, 6, 12mo)	WAZ: −4.13 (baseline) → −2.19 (3mo) → −2.71 (6mo) → −1.80 (12mo), net +2.33. Increasing *z*-score in 81% at 3mo, 44% at 6mo, 64% at 12mo.	Favourable (non-linear trajectory)	Serious
Arrowsmith 2010 (38)	21 (100%)	Prospective cohort	Median 19.4 months on gastrostomy feeds (IQR: 7.7–29.99 months)	Weight SDS improved (−4.8 → −3.0) but adiposity rose significantly, no height catch-up	Mixed	Serious
Catto-Smith 2006 (35)	98 (100%)	Retrospective cohort	6–14 years	WAZ +1.05	Favourable	Serious
Ulman 2021 (101)	109 (100%)	Retrospective cohort	Median > 2 years	Fundoplication subgroup: 100% significant weight gain. Cohort-wide *z*-score data not reported.	Favourable (fundoplication subgroup only)	Moderate
Wadman 2021 (99)	146	Retrospective chart review	Median 4 years (0–16)	BMI improved in 16/19 (84%) with paired data; growth velocity not quantified.	Favourable	Moderate/High
Franken 2015 (34)	300 (75% NI)	Retrospective cohort	Median 2.63 years	WLZ improved significantly: −0.98 → −0.26 (*p* < 0.0005). HAZ essentially unchanged: −1.62 → −1.54 (*p* = 0.695).	Mixed (favourable weight; NS height)	Moderate
Sharma 2012 (123)	41	Retrospective cross-sectional	12 months post-op	Weight-Z improved: −2.94 → −1.87 (*p* < 0.0001). Early GT (≤18mo) gave larger gain (+1.33) than late (>18mo, +0.45; *p* = 0.021). Height/BMI not recorded.	Favourable	Moderate/High
Toporowska-Kowalska 2011 (95)	15	Prospective study	6–8 months	Baseline BMIZ severely low (<−3 in 8/15; −2 to −3 in 7/15); post-op *z*-scores not reported, but median weight rose 22.0% (IQR: 14.4–29.2%) over 6mo.	Favourable	Moderate
Lefton-Greif 2011 (48)	28 (paired data *n* = 10)	Retrospective review	Mean 4.11 years	Paired BMIZ (*n* = 10): median change +0.87 (range −3.58 to +6.72, *p* = 0.32, NS); improved/stabilised in 70%, fell in 30%.	NS (small paired subset)	Moderate
Thomson 2011 (53)	10	Prospective observational	12–384 days (median 55)	Median weight gain 2.53 kg (range 0.8–7.24 kg) pre → post-PEG.	Favourable	Moderate/High
Vernon-Roberts 2010 (52)	14	Prospective, multi-centre	6 months	Weight-Z: −2.4 → −2.1 (NS, *p* = 0.069); absolute weight rose significantly (median +1.9 kg, *p* = 0.012). Micronutrients mostly normal; chromium elevated and rising, zinc/copper/B1 slightly out of range.	Mixed	Not appraised
Sullivan 2006a (32)	40	Prospective cohort	12 months	GT-fed (*n* = 22) vs. orally-fed (*n* = 18) CP children. Baseline weight-Z similar (GT −2.8 vs. oral −3.2); after adjustment for baseline/GMFCS, no between-group difference at 12mo (mean diff 0.002, *p* = 1.0).	NS (vs. oral comparator)	Serious
Sullivan 2005^a^ (33)	57	Longitudinal prospective, multi-centre	6 & 12 months	WAZ improved: −3.03 (baseline) → −2.23 (6mo) → −1.60 (12mo), *p* < 0.0001; 91% (42/46) had a WAZ increase over 12mo; daily energy/fat intake significantly increased.	Favourable	Serious
Puntis 2000 (77)	29 (27 PEG)	Retrospective cohort	Median 2.6 years	WAZ improved: −3.0 → −2.0 (*p* < 0.01); of 20 children below 3rd centile pre-PEG, 5 crossed above it.	Favourable	Serious
Valletta 2004 (116)	11	Longitudinal cohort	Median 15 months	W/H ratio increased significantly: 81.2% → 87.2% at 3mo (*p* = 0.002).	Favourable	Not appraised
Ramelli 2007 (56)	31 (100%)	Longitudinal cohort	1–19 years (mean 5)	Low weight-for-height fell 16/19 → 3/19 at 1yr; weight faltering reversed in 17/22, height faltering in 9/14 (most marked improvement).	Favourable	Serious
Park 2011 (78)	32	Retrospective cohort	Mean 12.2 months	Age-adjusted weight-Z improved: −1.34 → −1.01 at 6mo (*p* < 0.0001); 96.4% (27/28) gained weight (mean +1.03 kg at 6mo).	Favourable	Serious
Saadah 2009 (98)	25 (100%)	Retrospective cohort	6 months	Baseline WAZ −2.46 (75% underweight); significant gain at 3mo (*Δ*WAZ 0.33, *p* = 0.005) and 6mo (*Δ*WAZ 0.64, *p* < 0.001).	Favourable	Serious
Motil 2009 (36)	95 (100%)	Retrospective cohort	Mean 4.5 ± 3.9 years	Height/length-Z rose 1.8 → 2.6; lean body mass 116 → 151 g/cm height; BMIZ fell 1.9 → 0.7 as body-fat % rose 20% → 27%—improved linear growth/lean mass with a shift toward higher adiposity.	Mixed	Serious
Seguy 2002 (37)	12 (100%)	Retrospective cohort	1 year	WAZ improved significantly: −2.90 → −2.03 (*p* = 0.05). WHZ improved significantly: −3.47 → −1.84 (*p* < 0.001). HAZ: no significant change (−0.90 → −1.11 whole group; −0.94 → −1.00 in >1yr FU subgroup), trending slightly worse rather than improving.	Mixed (favourable weight indices; NS—slight worsening trend—height)	Serious
Thorne 1998 (112)	62 (61, ∼100%)	Retrospective cohort	≥6 months	S.H.A.P.E. screening tool indicated adequate caloric intake for body size in nearly all children; weight maintained or gained by all children over 12mo.	Favourable	Serious
Saleem 2023 (73)	40 (100%)	Prospective cohort	6 months	95% (38/40) gained weight post-PEG (mean +1.89 ± 1.0 kg); 2 lost weight (0.20, 0.30 kg), attributed to comorbid disease.	Favourable	Serious
Brant 1999 (13)	20	Prospective cohort	Mean 5.9 months	Significant overall weight gain (*p* < 0.01); significantly improved WHZ and mid-arm muscle area in under−4yr subgroup (*p* < 0.01); triceps skinfold change NS.	Favourable	Serious
Grayeb 2025 (39)	372 (327, 88% NI)	Retrospective cohort	Median 64 months	Weight-Z improved: −1.8 → −1.65 (*p* = 0.004). Iron deficiency in 64% tested, iron-deficiency anaemia in 31%; lower B12/folate/vitamin D deficiency rates in smaller tested subsets—none linked to recurrent respiratory hospitalisation.	Mixed (favourable growth; substantial residual micronutrient deficiency)	Low
Tawfik 1997 (89)	29	Retrospective cohort + caregiver questionnaire	1–5 years	Weight gain (pre-op problem in 15/15 applicable) improved post-op in all (*p* < 0.0001)—GT-alone and GT + fundoplication groups alike.	Favourable	Moderate/High
Wesley 1981 (90)	33 (22 NI subgroup)	Retrospective case series	Mean 11 months	Steady/accelerated weight gain contributed to a “good-to-excellent” outcome in 18/22 NI patients; no discrete quantitative pre/post values reported.	Favourable (qualitative)	Moderate/High
Heine 1995 (105)	30 (100%)	Prospective before/after	3–12 months	WAZ improved significantly: −2.99 → −2.36 (mean +0.64, *p* < 0.005); 21/26 followed children gained weight significantly (mean +3.1 kg); overfeeding in 2 children.	Favourable (2 cases of overfeeding)	Moderate
Shapiro 1986 (106)	19 (100%)	Retrospective before/after	Mean 23.1 months	W/L-Z improved: −2.71 → −1.18 (≈1.5 SD gain); 16/19 increased W/L ratio, 11 exceeding 10th percentile (*p* < 0.01); 3 had net W/L loss.	Favourable	Moderate/High
Stringel 1989 (91)	90 (100%)	Retrospective case series	3mo (weight); subgroup >1yr	Average weight gain 3.8 kg/patient at 3mo; significant in most patients; caloric intake deliberately reduced in some to avoid obesity.	Favourable	Serious
Sanders 1990 (40)	51 (100% CP)	Retrospective cohort, 3 groups by time since CNS insult	Mean 2.4 years	Percent-of-ideal weight/length/W-for-L improved in all 3 groups (earliest-intervention: weight 71% → 88%, W-for-L 93% → 99%); weight-gain velocity 3.3, 2.8, 1.5 g/kg/day (early, middle, late intervention)—earlier GT gave greater malnutrition reversal.	Favourable	Moderate
Pinheiro 2025 (42)	18 (100% congenital Zika syndrome)	Prospective cohort, before-after	∼1 year	WAZ improved significantly: −3.0 → −2.0 (*p* < 0.05; improved in 14/18); LAZ and BMIZ-for-age also improved in most, though 39% remained markedly underweight and body-fat % rose significantly (25.2% → 29.3%, *p* = 0.017).	Mixed (significant improvement, incomplete catch-up, rising adiposity)	Serious
Tarek 2024 (92)	34 (100% severe NI, GMFCS 4–5)	Prospective observational	6 months	BMI improved significantly in both feed-type arms (peptide 14.9 → 20.4; casein 16 → 20.9; both *p* < 0.001), with significant WAZ/StatZ improvement also (*p* < 0.001 overall).	Favourable	Serious
Salomone 2022^b^ (126)	24 (100%)	Retrospective, before-after	6mo & 1yr	WAZ and BMIZ improved significantly at 6mo and 1yr (*p* = 0.015–0.035); vitamin B12, folate, and iron rose significantly, correcting baseline deficiencies.	Favourable	Not appraised
Jennuvat 2023 (49)	39 (100%)	Cross-sectional + retrospective pre/post	6 months & 3 years	No significant change in WAZ, HAZ, or WHZ at 6mo or 3yr; authors attributed lack of catch-up growth to low household income.	NS	Moderate/High
Işık & Akbulut 2021 (68)	91 (70, 76.9% NI)	Retrospective, single-centre	6 months–5 years	3 GTs removed once oral intake became sufficient; detailed growth metrics not reported.	Favourable (qualitative)	Serious
Noviello 2021 (75)	148 (111, 75% NI)	Retrospective review	Variable (6-monthly review)	All patients gained weight; average WAZ improved by ≈2 SD.	Favourable	Moderate
Franken 2020b (47)	50 (72% NI overall)	Prospective longitudinal cohort	Median 3.9 months	Weight-for-height percentile rose 30 → 55 overall (NS, mean diff +5.2) and 70 → 55 in NI subgroup (also NS).	NS	Serious
Martínez-Costa 2019 (6)	65 (40, 61.5% NI)	Prospective, multi-centre cohort	12 months	Weight-Z improved −1.96 → −1.13 (6mo) → −1.22 (12mo); HAZ improved −2.64 → −2.47 → −2.44. Most BMI gain occurred in first 6mo; height gains continued linearly through 12mo, especially with GT before 18mo of age.	Favourable	Serious
Lertudomphonwanit 2019 (59)	201 (168, 83.6% NI)	Retrospective cohort	1 year	WAZ improved significantly: −2.13 → −1.38 at 1yr (*p* = 0.0001); median weight increase 38.9%; underweight (z < −2) fell 45.3% → 39.7%.	Favourable	Serious
Romano 2016 (130)	56 (100%)	Prospective	6 months	Average weight increase 800 g over follow-up.	Favourable	Not appraised
Gębora-Kowalska 2012^c^ (41)	21 (100% NI)	Prospective longitudinal cohort	12 months (baseline, 6mo, 12mo)	BMIZ improved significantly: −4.83 (baseline) → −3.40 (6mo) → −2.39 (12mo), *p* < 0.001. Cachectic children (BMIZ > 3SD below average) fell 52.4% → 19.0% (*p* < 0.001). Protein and albumin significantly increased by 12mo; transferrin rose but NS.	Favourable	Moderate
Vergara 2011 (26)	55 (100% CP)	Prospective comparative cohort (GT vs. NFD vs. non-surgical)	Not reported	Weight percentile improved significantly after GT (*n* = 10 evaluated, *p* = 0.043) and after NFD (*p* = 0.002); non-surgical group showed no improvement. Baseline malnutrition: severe 45%, moderate 21.8% (whole cohort).	Favourable (vs. non-surgical comparator)	Not appraised
Finbråten 2011 (30)	661 (100% CP)	Registry-based cross-sectional (Norwegian CPRN)	N/A (cross-sectional)	Cross-sectional BMI status: 63% normal, 10% thinness/severe thinness, 16% overweight/obese. Growth retardation associated with earlier GT insertion (*p* < 0.001) and greater GMFCS severity.	Not assessable (cross-sectional, no baseline)	Moderate
Zhang 2025 (27)	84 (100% NI, TBI-dysphagia)	RCT (42 vs. 42; journal labels article “Observational Study”)	28 days post-treatment	Weight increased from baseline in PEG group (*p* < .05) and was significantly higher than NG-control at 28d (*p* < .05); Serum total protein, albumin, and haemoglobin also rose more in PEG group (*p* < .05).	Favourable (vs. NG comparator)	High
Wu 2026 (28)	74 (100% NI, acquired brain injury)	Retrospective cohort (GT vs. continued NG at inpatient rehab admission)	Inpatient rehab stay only, mean 43.2 days	BMI at admission: NG 13.2 vs. GT 14.8 (*p* = .07, NS). Weight gain across stay: GT 5.1kg vs. NG 2.9kg (*p* = .2, NS). Underweight at admission: GT 11/16 vs. NG 5/16 (*p* = .1, NS).	No difference (vs. NG comparator)	Serious
Demirbaş 2021 (44)	20 (100% NI)	Prospective comparative cohort (GT-alone vs. GT + Nissen fundoplication)	Not reported (pre/post pH monitoring and anthropometrics)	WAZ improved significantly in both arms (GT-alone *p* = .043; GT + Nissen *p* = .040). BMIZ improved significantly in both arms (GT-alone *p* = .040; GT + Nissen *p* = .002).	Favourable	Serious
Chae-ri Suh 2020 (7)	18 (30 recruited, 12 excluded; 100% NI)	Retrospective cohort with structured paediatric nutrition-team intervention	6 months & last visit	BMIZ improved significantly at 6 months (*p* = 0.0005). HAZ improved significantly at 6 months and last visit (*p* = 0.034). WAZ improved (*p* = 0.011). Percent-ideal-body-weight improved significantly at 6 months (*p* = 0.003) and overall (*p* = 0.041), reaching ∼100% by 6 months.	Favourable	Moderate
Tavares de Sousa 2020 (31)	28 (100% spastic quadriplegic CP)	Cross-sectional	N/A (cross-sectional)	Cross-sectional BMI status: 57% eutrophic, 39% malnourished, 4% overweight.	Not assessable (cross-sectional, no baseline)	Moderate
O'Loughlin 2013 (43)	122 (100% severe NI)	Retrospective cohort + cross-sectional caregiver questionnaire	Not specified	WAZ improved significantly: −2.57 ± 0.16 → −1.65 ± 0.20 (*p* < 0.00005). 83/89 caregivers (93%) reported weight gain was better or much better post-op.	Favourable	Moderate/High
Samuel 2016 (45)	32 (100% NI)	Retrospective study	3 months	WAZ improved: −1.86 → −1.47 (*Δ* = −0.4; range −0.02 to 1.76).	Favourable	High
Chao 2009 (46)	46 (39 completed; 100% NI)	Retrospective cohort	Not specified	Mean body-weight percentile rose 15th → 17th (*p* = 0.64, NS overall); weight caught up by >10 percentiles in 8/39 children (20%).	Mixed	Serious

WAZ, weight-for-age *z*-score; HAZ, height-for-age *z*-score; WHZ, weight-for-height *z*-score; WLZ, weight-for-length *z*-score; W/L-Z, weight/length *z*-score; StatZ, stature(-for-age) *z*-score; BMIZ, BMI *z*-score; MUAC, mid-upper arm circumference; NS, not significant; GT, gastrostomy(-fed); NG, nasogastric; NFD, nasal-feeding device.

a Sullivan 2005 is shown as the representative publication for this 57-child multicentre cohort; companion papers from the same cohort (Sullivan 2004, caregiver QoL only—no nutritional outcome; Sullivan 2006b, interim report deferring growth data to this paper) are not shown separately.

b Salomone 2022 is the larger, later-cutoff analysis (*n* = 24, data to March 2022) of the same Naples cohort reported in Salomone 2023 (*n* = 17, data to December 2020); the earlier report is not shown separately.

c Gębora-Kowalska 2012 shares four of its five authors with Toporowska-Kowalska 2011; data were extracted from the abstract only, as the full text was unavailable.

**Figure 1:**
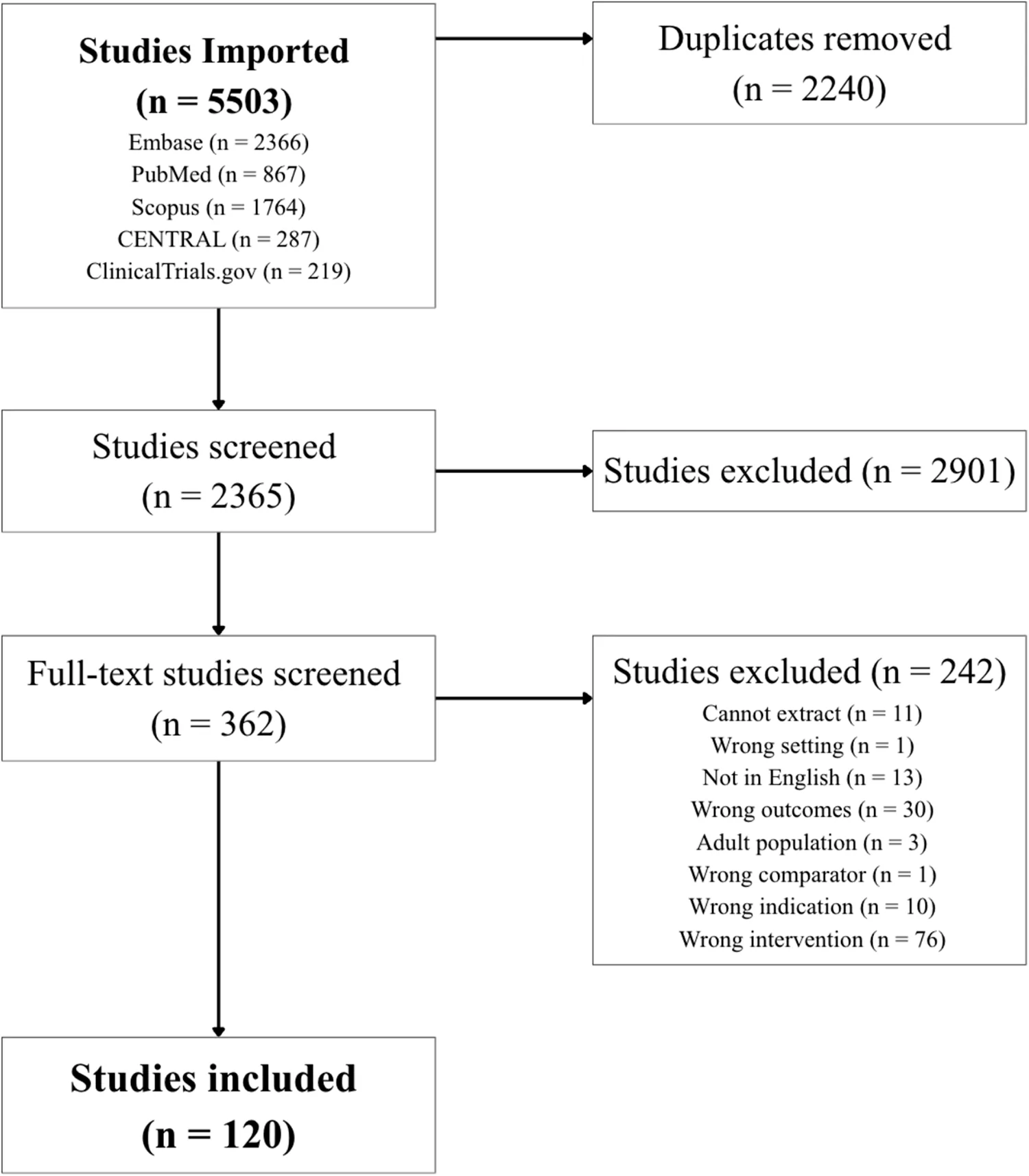
PRISMA flow Diagram.

**Table 4:** Respiratory outcomes after gastrostomy.

Author/Year	N (NI)	Design	Follow-up	Finding	Direction	Risk of bias
Zhang 2025 (27)	84 (84, 100%)	Randomised controlled trial (PEG vs. indwelling NG)	28 days	Aspiration pneumonia during the 28-day treatment period occurred in 1/42 (2.4%) of PEG patients versus 8/42 (19.0%) of NG controls (*p* = 0.030). Incidence during treatment, not a before-versus-after comparison.	Favourable (vs. NG comparator)	High
Grayeb 2025 (39)	372 (327, 88%)	Retrospective cohort	Median 64 months	62% (230/372) had recurrent hospitalisations (≥2/year), of which 52% were respiratory. Predictors of recurrent respiratory hospitalisation: PPI use HR: 1.88 (1.46–3.07, *p* = 0.033), inhaled steroids/bronchodilators HR: 1.45 (1.28–3.25, *p* = 0.003), seizure history HR: 1.50 (1.30–6.64, *p* = 0.014). No baseline comparison.	Not assessable (no before/after comparison; high residual burden)	Low
Clayton 2024 (88)	21 record/26 survey (100% NI, Dravet)	Retrospective record review + cross-sectional caregiver survey	Mean 3.4–6.1 years	Caregivers were asked whether admissions for pneumonia changed after gastrostomy; the reported direction was fewer admissions or no difference, but responses are presented only as a pie chart, so no numerators are extractable.	Favourable (qualitative; not quantifiable)	Moderate/High
Tarek 2024 (92)	34 (34, 100%; GMFCS 4–5)	Prospective observational (peptide vs. casein formula)	6 months	Aspiration pneumonia decreased significantly in both arms (*p* = 0.000 and *p* = 0.016); pre-gastrostomy aspiration-pneumonia admission rates were 70.6% and 64.7%.	Favourable	Serious
Jennuvat 2023 (49)	39 (39, 100%)	Cross-sectional + retrospective pre/post questionnaire	Median 33 months	Respiratory tract infections decreased in 84.6% of children; aspiration-pneumonia admissions fell from a median of 1 to 0.5 per year (*p* < 0.001); overall admission frequency decreased in 87.2%.	Favourable	Moderate/High
Jadi 2023 (71)	63 (NI subgroup)	Retrospective chart review	12 months	3 patients (4.8%) had post-operative pulmonary aspiration or pneumonia; no pre-operative comparison reported.	Not assessable (post-operative events only)	Serious
Dreznik 2022 (80)	345 (158, 45.7%)	Retrospective cohort	Median 5 years (1–10)	Recurrent aspiration pneumonia was used to define clinical improvement (>1 hospitalisation/year = lack of improvement), but event counts are not reported.	Not assessable (outcome defined but not quantified)	Serious
Lin 2021 (122)	2,490 (2,490, 100%)	Retrospective 1:2 propensity-matched cohort	180 days (sensitivity to 365/548 days)	Subsequent severe pneumonia hospitalisation occurred in 32% (789/2,490). New gastrostomy was associated with an increased risk of the composite outcome of death or severe pneumonia (HR: 1.21), although mortality alone was reduced (HR: 0.47).	Unfavourable (no reduction in pneumonia admissions)	Moderate
Ulman 2021 (101)	109 (109, 100%)	Retrospective cohort (routine vs. selective GER screening)	Median >2 years	Among patients who had a fundoplication and a history of recurrent pulmonary infection, 67% reported no pulmonary infections and 33% reported decreased frequency after surgery.	Favourable	Moderate
Zambaiti 2021 (100)	145 (145, 100%)	Retrospective cohort (gastrostomy only vs. gastrostomy + fundoplication)	Median 4 years (1–12)	Length of stay per respiratory admission fell from 10 days pre-operatively to 4 days post-operatively across all patients. The gastrostomy-only group had significantly fewer respiratory admissions per year than the fundoplication group (0.32 vs. 1.00; *p* = 0.004), but ED attendances for respiratory issues rose in the gastrostomy-only group (0.37 to 0.65). 3 patients died of proven aspiration.	Mixed	Serious
Demirbaş 2021 (44)	20 (20, 100%)	Prospective (gastrostomy vs. gastrostomy + Nissen)	Mean 16.5 months	Lung infections decreased in 6/8 (75%) of Group 1 and 7/8 (87.5%) of Group 2.	Favourable	Serious
Wadman 2021 (99)	146 (23 gastrostomy)	Retrospective chart review	Median 4 years (0–16)	15 of the 23 gastrostomy patients had recurrent chest infections before the procedure; 12 of those 15 (80%) reported no chest infections afterwards.	Favourable	Moderate/High
Suh 2020 (7)	18 (18, 100%)	Retrospective cohort	Median 50.4 months	No new aspiration events were quantified during the intervention period.	Not assessable	Moderate
Tavares de Sousa 2020 (31)	28 (28, 100% CP)	Cross-sectional	Not applicable	Aspiration pneumonia in 14% (*n* = 4), recurrent flu in 18% (*n* = 5) and recurrent infections in 7% (*n* = 2); cross-sectional design with no baseline.	Not assessable (no before/after comparison)	Moderate
Glasson 2020 (108)	21 caregivers (21, 100%)	Qualitative interview study	Median 2.5 years of gastrostomy use	Caregivers described prior recurrent chest infections, pneumonia and aspiration, with qualitative improvement and fewer pneumonia-related hospitalisations after gastrostomy. No quantitative data.	Favourable (qualitative)	Low
Jacoby 2020 (50)	673 (673, 100%)	Population-based retrospective cohort, self-controlled	Median 4.8–7.5 years	Acute lower respiratory tract infection admissions increased in the first post-operative year (IRR: 1.26) and remained elevated at ≥6 years (IRR: 1.42); gastrostomy was not protective. Adding a fundoplication reduced relative LRTI incidence by year 3 (IRR: 0.78 vs. 1.35). All-cause admissions declined substantially by 5 years. ^a^Same WA/NSW linked cohort as Downs 2019.	Mixed (unfavourable for LRTI; favourable for all-cause admissions)	Low
AlNamshan 2019 (55)	178 (178, 100%)	Retrospective cohort	Median 36–44 months	Recurrent aspiration was present in 127 (71.3%) pre-operatively. GER/aspiration admissions fell from 0.67 to 0.09 per person-month (*p* < 0.001) and all hospital admissions from 0.95 to 0.13 per person-month (*p* < 0.001); admissions for pneumonia and respiratory symptoms decreased significantly.	Favourable	Serious
Nelson 2019 (57)	948 (948, 100%)	Population-based exposure-crossover study (2 years before vs. 2 years after)	Up to 5 years	Reflux-related hospital days and acute health-care use were stable before and after tube placement. Median admissions fell from 2 (IQR: 0–3) to 1 (IQR: 0–3) with unchanged median length of stay; weekly rates of unplanned hospital days were stable (adjusted RR: 0.92, 95% CI: 0.57–1.48).	No difference	Low/Moderate
Downs 2019 (51)	673 (673, 100%)	Retrospective birth-cohort study (pre/post)	≥4 years post-gastrostomy	Admissions for acute respiratory tract infection increased in the WA cohort and remained similar in the combined cohort, while all-cause hospitalisations and epilepsy admissions declined. ^a^Same cohort as Jacoby 2020.	Mixed	Not appraised
Wadman NR (99)	47 (14 gastrostomy)	Retrospective chart review (pre/post)	Mean 6 years (1–15)	Improvement after gastrostomy in the 6 patients who had recurrent pulmonary infections.	Favourable (small subgroup)	Not appraised
Figueroa 2017 (29)	81 (81, 100%)	Prospective cohort	1 year	29 children (35.8%) required hospitalisation within the first year, half for less than 7 days and 9 more than once; the main cause was respiratory infection. No pre-gastrostomy comparison.	Not assessable (no baseline comparison)	Moderate
Koca 2015 (74)	34 (34, 100%)	Retrospective cohort	Mean 19.4 ± 10.3 months	One child with cerebral palsy developed aspiration pneumonia after PEG and subsequently underwent Nissen fundoplication.	Not assessable (isolated post-operative event)	Serious
Barnhart 2013 (104)	4,163 (4,163, 100%)	Retrospective propensity-matched cohort (fundoplication + GT vs. GT alone)	1 year post-NICU discharge	Aspiration pneumonia admissions averaged 0.08 in both arms; GERD hospitalisations 0.65 (fundoplication) vs. 0.53 (GT alone); mechanical ventilation 0.29 vs. 0.38. Concomitant fundoplication conferred no respiratory advantage.	No difference	Moderate
O'Loughlin 2013 (43)	122 (122, 100%)	Retrospective cohort + caregiver questionnaire	Median 77 months	Chest infections were reported as better or much better by 74 of 89 caregivers (83%); however, 13 patients died of pneumonia, mostly attributable to recurrent aspiration.	Mixed (caregiver-reported improvement; persistent aspiration mortality)	Moderate/High
Sharma 2012 (123)	41 (41, 100%)	Retrospective cross-sectional (early vs. late insertion)	12 months	No significant difference in admissions before versus after gastrostomy overall (*p* = 0.384); early-insertion group 6.00 to 4.38 mean admissions (*p* = 0.133), late group 2.12 to 2.94 (*p* = 0.241).	No difference	Moderate/High
Mahant 2011 (107)	16 caregivers (16, 100%)	Qualitative interview study	≥1 year post-procedure	Acute-care visits and admissions for respiratory illness including aspiration pneumonia were described narratively alongside feeding intolerance and tube problems.	Not assessable (qualitative)	Low
Toporowska-Kowalska 2011 (95)	15 (15, 100%)	Prospective cohort	6–8 months	By caregiver report, the rate of ambulatory-treated respiratory tract infections was decreased or unchanged in all children relative to the preceding 6 months; no numerical analysis was performed. ^b^Complication data from this study were found unreliable elsewhere in the review—treat with caution.	Favourable (qualitative, low certainty)	Moderate
Thomson 2011 (53)	10 (10, 100%)	Prospective before-after pH/impedance study	Median 55 days (12–384)	Parents reported no change in the frequency of chest aspiration or infection after PEG, but the proportion of reflux events reaching the pharyngeal space rose from 56% to 82% (median 9.0 to 27.0 events, *p* = 0.022), which the authors interpret as an increased risk of aspiration pneumonia.	Unfavourable (physiological); No difference (clinical)	Moderate/High
Kawahara 2010 (97)	54 (54, 100%)	Retrospective cohort	Median 4 years (2–7)	Post-operatively, one patient had repeated aspiration pneumonia and one had pneumonia once or twice per year; aspiration was documented in two patients in total.	Favourable (low residual respiratory morbidity)	Moderate
Vernon-Roberts 2010 (52)	14 (14, 100%)	Prospective longitudinal feasibility study (pre/post)	6 months	Chest infections requiring antibiotics decreased in 50% of participants and 25% had none before or after. For admissions: 6 of 8 followed children had none throughout, 1 had more and 1 had fewer.	Favourable	Not appraised
Viswanath 2010 (61)	137 (137, 100%)	Retrospective cohort across three management protocols	Median 3.1 years	Post-operative pneumonia occurred in 1/82 (1.2%) gastrostomy-alone patients and 3/60 (5.0%) fundoplication patients.	No difference (low post-operative pneumonia rate in both arms)	Serious
Sathesh-Kumar 2009 (70)	76 (≈76% NI)	Cohort study	Median 52 months	One patient died of aspiration pneumonia while awaiting antireflux surgery; respiratory outcomes not otherwise reported.	Not assessable	Moderate/High
Chao 2009 (46)	46 (39 completed, 100% NI)	Retrospective cohort (pre/post)	Mean 25 months (7 months–6 years)	Pneumonia-related admissions fell from 1.6 to 0.6 per patient (*p* < 0.001), duration of pneumonic admission from 83 to 40 days (*p* = 0.02), and ICU admissions from 0.45 to 0.13 (*p* = 0.006).	Favourable	Serious
Ramelli 2007 (56)	31 (31, 100%)	Longitudinal cohort	12 months–19 years (mean 5 years)	Pre-operatively 29 children had a positive history of chest infection (15 very frequent, 17 requiring hospitalisation); at review 26 had had no further chest infections, and hospitalisations for chest infection were significantly reduced.	Favourable	Serious
Vajsar 2006 (58)	12 (12, 100% SMA type 1)	Retrospective case series	30 days (by design)	Aspiration pneumonia in 5/12 (41.6%) and respiratory failure requiring PICU admission in 4/12 (33%) within 30 days of insertion; affected children were significantly older at insertion (*p* < 0.05).	Unfavourable	Not appraised
Sullivan 2006 (11)	57 (57, 100%)	Prospective longitudinal cohort (pre/post)	6 and 12 months	Mean number of chest infections requiring antibiotics fell from 1.8 at baseline to 0.9 at 12 months; hospital admissions for chest infection fell from a mean of 0.5 to 0.09 (*p* = 0.04), and by 12 months no child had been admitted for a chest infection. ^a^Same Oxford cohort as Sullivan 2005.	Favourable	Moderate/High
Petersen 2006 (65)	26 (26, 100%)	Descriptive semi-structured interview study	Mean 3 years 1 month	Caregivers subjectively reported fewer periods of hospitalisation and reliable medication administration during illness; no quantitative respiratory data.	Favourable (qualitative, low certainty)	Not appraised
Sullivan 2005 (33)	57 (57, 100%)	Prospective longitudinal cohort (pre/post)	12 months	The proportion of children with at least one chest infection requiring hospital admission fell from 11/43 (26%) at baseline to 3/43 (7%) at 12 months (*p* = 0.021). ^a^Same Oxford cohort as Sullivan 2006.	Favourable	Serious
Harrington 2004 (103)	102 (102, 100%)	Retrospective cohort (gastrostomy ± concurrent antireflux procedure)	2 years post-discharge	Readmission recorded only for subsequent antireflux operation; no respiratory admission or infection data reported.	Not assessable	Moderate
Wales 2002 (84)	111 (111, 100%)	Retrospective cohort (fundoplication + gastrostomy vs. gastrojejunal tube)	Mean 3.1 years (GJ)/5.7 years (FG)	Aspiration pneumonia recorded as a major complication in 31.3% (GJ) vs. 36.5% (FG), *p* = 0.68. Per the authors’ discussion response, pre-operative aspiration fell to approximately 15% (FG) and 17% (GJ) post-operatively, although these figures do not appear in the results text.	Mixed	Serious
Seguy 2002 (37)	12 (12, 100%)	Retrospective cohort	1 year	The 3 patients with severe gastro-oesophageal reflux developed at least 3 pneumonias each after gastrostomy.	Unfavourable (reflux subgroup)	Serious
Puntis 2000 (77)	29 (29, 100%)	Retrospective cohort	Median 2.6 years (0.4–4.9)	No clinical aspiration events were reported, and autopsies of children who died showed no evidence of feed aspiration; one child required readmission for intravenous antibiotics for peri-tube sepsis.	Favourable	
Smith 1999 (9)	40 interviewed (100% CP)	Population-based retrospective cohort + caregiver interview	Mean age at follow-up 8.5 years	Aspiration was documented pre-operatively in 22/40 (55%); of these, 8/22 (36.4%) improved after tube feeding (*p* = 0.02). ^b^The hospitalisation field for this study duplicates Wales 2002 figures verbatim and was disregarded.	Favourable (modest)	Moderate/High
Brant 1999 (13)	20 (20, 100%)	Prospective cohort	Mean 5.9 months	GERD-related pneumonia in 3 cases (15%); no pre-operative comparison reported.	Not assessable (post-operative events only)	Serious
Tawfik 1997 (89)	29 (29, 100%)	Retrospective cohort + structured caregiver questionnaire	1–5 years	Chest infections improved in 15/27, worsened in 2 and were unchanged in 6—not statistically significant overall or in either subgroup. Coughing/choking improved in 19/20 (*p* < 0.0001). For consultations, 4 required more, 9 no change and the remainder fewer.	Mixed (significant for choking; not significant for chest infections)	Moderate/High
Borowitz 1997 (72)	19 (19, 100%)	Prospective cohort (pre/post)	Mean 20.7 months (7–33)	Of 8 children with a pre-operative history of chest infection, 5/8 (62.5%) improved, 1/8 (12.5%) was unchanged and 2/8 (25%) worsened.	Mixed	Serious
Cameron 1995 (62)	63 (63, 100%)	Retrospective cohort (PEG vs. Stamm gastrostomy)	Mean 23 months (minimum 12)	Pre-operative aspiration/pneumonia in 13% (PEG) and 27% (Stamm); post-operative pneumonia or apnoea occurred in 0 PEG patients and 4 Stamm patients.	Favourable (PEG)	Serious
Heine 1995 (105)	30 (30, 100%)	Prospective before/after with caregiver questionnaire, pH study and endoscopy	3–9 months (questionnaire)	Chest infections were significantly reduced after PEG: increased in 2/27, unchanged in 16 and decreased in 9 (*p* < 0.05). Recurrent aspiration contributed to reflux exacerbation in 2 of 5 children requiring fundoplication, and chronic salivary aspiration was implicated in deaths.	Favourable	Moderate
Vergara 2011 (26)	55 (55, 100% CP)	Prospective comparative cohort (gastrostomy + Nissen vs. no surgery)	Not reported	Hospitalisation duration was shorter in the surgical group (18.8 vs. 42.7 days); causes of re-hospitalisation across the cohort included pneumonia, malnutrition and seizures.	Favourable	Not appraised
Park 2011 (78)	32 (32, 100%)	Retrospective cohort (pre/post)	Mean 12.2 months (SD 6.6)	No aspiration events recorded post-operatively.	Favourable	Serious
Kehar 2014 (54)	46 (≈74% NI)	Prospective cohort	≥1 year	Aspiration pneumonia in 4.3%; no pre-operative comparison reported.	Not assessable (post-operative events only)	Serious
Khattak 1998 (79)	120 (52, 43%)	Retrospective case series	Mean 26.6 months (3 months–6 years)	Chest infection was not quantified before versus after, and aspiration was not separable for the NI subgroup; 7 children required hospitalisation for stomal complications.	Not assessable (not NI-separable)	Moderate
Langer 1988 (83)	107 (107, 100%)	Retrospective cohort (gastrostomy ± antireflux procedure)	Mean ≈ 20 months (1–66)	Recurrent pneumonia was almost universal both before and after surgery and was not quantified; its high baseline prevalence prevented evaluation of any antireflux effect on pulmonary status. 2 of 4 deaths were aspiration-related.	Not assessable	Serious
Sanders 1990 (40)	51 (51, 100% CP)	Retrospective before-after cohort	6 months–5.5 years (mean 2.4)	Aspiration pneumonia was the cause of deaths, particularly after enteral feeds were stopped and oral feeding resumed; not quantified before versus after.	Not assessable	Moderate
Stringel 1989 (91)	90 (90, 100%)	Retrospective case series with pre/post data	2 months–3 years	Aspiration secondary to gastro-oesophageal reflux was the main pre-operative problem and repeated pre-operative pulmonary admissions were noted, but post-operative events were not quantified.	Not assessable	Serious
Wesley 1981 (90)	33 (22, 66.7%)	Retrospective case series	Mean 11 months (2 months–2.5 years)	Aspiration pneumonia was a presenting symptom in 5/22 and a cause of death during follow-up. Post-operatively, one patient had 4 recurrent bouts of aspiration pneumonia and one had recurrent pneumonia attributable to cleft palate rather than reflux recurrence.	Mixed	Moderate/High

Chest-infection frequency before versus after gastrostomy, respiratory and aspiration-related hospitalisations, and aspiration events. GT, gastrostomy(-fed); NG, nasogastric; GJ, gastrojejunal; LRTI, lower respiratory tract infection; IRR, incidence rate ratio; HR, hazard ratio; NS, not significant.

a Companion publications reporting the same cohort.

b Data-quality caution, as noted in the cell.

**Table 5:** Minor complications after gastrostomy.

Author/Year	N (NI)	Design	Follow-up	Finding	Direction	Risk of bias
Craig 2006 (124)	76 (76, 100%)	Prospective cohort	6 months post-op	Granulation tissue 47.3%, infection 39.2%, retching/vomiting 33.8% each, leakage 23.3%, excoriation 13.5%, gas-bloat 9.5%. 13 children (17%) had ≥1 major complication (internal fistula, migration, adhesions, haemorrhage, redo fundoplication).	Unfavourable	Moderate
Catto-Smith 2006 (35)	98 (100%)	Retrospective cohort	6–14 years	Fever 17%, cellulitis 7%, peritonism 4%, distension 3%, aspiration 2%, vomiting 2%, ileus 1%, bleeding 1%	Mixed	Serious
Dipasquale 2023 (8)	50 (50, 100%)	Retrospective cohort	Not reported	8/50 (16%) developed a minor complication—tract disruption, granuloma, or peristomal infection; no major complications.	Favourable	Moderate
Arnbjörnsson 1999 (125)	98	Retrospective cohort	Mean 39 months	Infection in 9 (8 medically treated, 1 device removal), granulation tissue in 31 (25 cauterised, 6 excised), peristomal pain in 8, leakage in 14 (10 self-resolved, 2 temporary removal, 1 redo, 1 permanent removal), transient feeding intolerance in 11, minor upper-GI bleed in 8. No majors.	Mixed	Serious
Fascetti-Leon 2012 (76)	239 (189, 79% NI)	Prospective, multi-centre	24 months (serial)	Granulation tissue rose 14.2% (1mo) → 34.3% (24mo); infection/phlegmon low throughout (2.5%–4.7%); leakage low (0.4%–4.7%). Majors (gastrocolic fistula, haemorrhage, displacement) infrequent (≤1.7% any timepoint).	Mixed	Moderate
Jadi 2023 (71)	63	Retrospective chart review	≥1 year	69.8% (44/63) had ≥1 complication; “functional tube problems” (dislodgement, clogging, leakage, pressure wounds) most frequent at 68.3%; infection (cellulitis/abscess) 6.3%; site bleeding 4.8%. 1 major (bowel perforation during GJ conversion, 1.6%).	Unfavourable	Serious
Ulman 2021 (101)	109 (100%)	Retrospective cohort	Median > 2 years	Fundoplication subgroup: gas-bloat 28.6% (self-resolving), new-onset drooling 8.6%. 2 patients (1.8% of total cohort) had major GER recurrence requiring reoperation.	Favourable	Moderate
Sakamoto 2021 (69)	89 (82 analysed)	Retrospective cohort	Short (<6mo) & long (>2yr)	Short-term: granulation 59.8%, skin trouble 14.6%, infection 12.2%, leakage 11.0%. Long-term: leakage 19.5%, granulation 18.3%, skin trouble 15.9%, infection 0%. Majors (ileus, peritonitis) rare, confined to short-term (1.4% each).	Mixed	Moderate
Zambaiti 2021 (100)	145	Retrospective cohort	Median 4 years	Short-term minor 30% (systemic infection, feeding difficulties/vomiting); long-term minor 24% (tube malfunction, local infection/granuloma). Majors 9% short-term, 15% long-term (respiratory distress, systemic infection, displacement).	Unfavourable	Serious
Franken 2020a^a^ (14)	150 (74% NI)	Cross-sectional	Median 4.0 years	Minor complications in 90.7% of whole cohort (not NI-isolated): hypergranulation 60.7%, infection 48.7%, dislodgement 43.3%, obstruction 23.3%. 4 pts (3.4%) converted to jejunal feeding.	Unfavourable	Moderate/High
Franken 2015 (34)	300 (75% NI)	Retrospective cohort	Median 2.63 years	Minor complications in 73.7% (408 events): hypergranulation 44%, stomal infection 24.7%, leakage 24%, dislodgement 21%, obstruction 6.3%. Majors uncommon (2.0%, mainly gastric-wall dehiscence).	Unfavourable	Moderate
Lefton-Greif 2011 (48)	28	Retrospective review	Mean 4.11 years	Wound leakage 36% (9/28), wound infection 4%, dislodgement 4%. 3 patients (11%) died within 30 days of placement (pneumonia, pneumothorax, respiratory failure)—reflects population severity, not a procedural complication *per se*.	Mixed	Moderate
Sathesh-Kumar 2009 (70)	76 (76% NI)	Cohort study	Median 52 months	Early (≤30d): peritubal infection (*n* = 6), leak (*n* = 1), pyrexia of unknown origin (*n* = 1), chest infection (*n* = 1). Late (>30d): peritubal infection/granulomata (*n* = 16), buried bumper (*n* = 9), migration (*n* = 4), gastrocutaneous fistula (*n* = 3). 1 major (gastric volvulus, laparotomy).	Mixed	Moderate
Sullivan 2005^a^ (33)	57	Longitudinal prospective, multi-centre	6 & 12 months	Site infection 59%, granulation tissue 42%, leakage 30%, tube blockage 19%, migration 7%, self-extubation 4%. 1 patient (2%) had severe leakage/peritonitis requiring stoma closure and delayed re-siting.	Unfavourable	Moderate
Puntis 2000 (77)	29 (27 PEG)	Retrospective cohort	Median 2.6 years	Among 27 successful PEG placements: over-granulation 37%, split tube 15%, infection 7%, tube blockage 7%. Majors: open conversion 6.9%, gastrocolic fistula 3.4%, need for post-op ventilatory support 10.3%.	Mixed	Serious
Ramelli 2007 (56)	31 (100%)	Longitudinal cohort	1–19 years (mean 5)	Local site infection in 9/31 (29%); majors limited to single cases of dislodgement and obstruction.	Mixed	Serious
Park 2011 (78)	32	Retrospective cohort	Mean 12.2 months	Wound infection/redness 13% (4/32) as a late complication; overall complication rate 47% including majors (transient pneumoperitoneum, paralytic ileus, atelectasis, gastrocolic fistula).	Mixed	Serious
Seguy 2002 (37)	12 (100%)	Retrospective cohort	1 year	Isolated single cases of abdominal wall abscess, skin ulceration, external granulation, intragastric pseudopolyp; peristomal redness in 2; device-failure peri-GT leaks in 6/12. No majors.	Mixed	Serious
Thorne 1998 (112)	62 (61, ∼100%)	Retrospective cohort	≥6 months	Highest minor-complication rates in this table: disconnection 83.9%, constipation 79.0%, vomiting/retching 72.6% each, leakage around device 74.2%, leakage through device 62.9%, RTI 61.3%, diarrhoea 56.0%, granulation 50.0%, skin irritation 48.4%, blockage 38.7%, distension 35.5%, dislodgement 32.3%, site infection 27.4%. No majors.	Unfavourable	Serious
Saleem 2023 (73)	40 (100%)	Prospective cohort	6 months	Wound infection 7.5%, irritability 2.5%, vomiting 2.5%, accidental removal 5.0%. No majors.	Favourable	Serious
Brant 1999 (13)	20	Prospective cohort	Mean 5.9 months	Granulation tissue 35% (7/20), stoma infection 20% (4/20), tube perforation 15% (3/20, plug replacement). 1 major (pneumoperitoneum, 5%).	Mixed	Serious
Tawfik 1997 (89)	29	Retrospective cohort + caregiver questionnaire	1–5 years	13/29 caregivers (45%) reported no GT-related problems. Among those who did: dislodgement (*n* = 4), infection/granulation mainly early post-op (*n* = 5), blocked/leaking tube (*n* = 4), diarrhoea requiring feed change (*n* = 1). Wound problems most-cited “worst aspect” (7/29). No majors.	Favourable	Moderate
Langer 1988 (83)	107 (100%)	Retrospective cohort (fundoplication ± anti-reflux procedure)	Mean ∼20 months	Fundoplication-alone (no anti-reflux procedure) group: wound infection in 5, dislodgement requiring reinsertion in 1 of 71 children. More serious complications (bowel obstruction, wrap herniation, gas-bloat) occurred only with an added anti-reflux procedure.	Favourable	Serious
Khattak 1998 (79)	120 (52, 43% NI)	Retrospective case series	Mean 26.6 months	Whole-cohort data (not NI-isolated): 43 minor complications in 27/120 (22.5%)—external leak (*n* = 10), infection (*n* = 9), granulation (*n* = 7), tube problems (*n* = 6), recurrent chest infection (*n* = 4). 51 majors in 21/120 (17.5%), incl. reflux requiring anti-reflux surgery (*n* = 17) and peritonitis (*n* = 8).	Mixed	Moderate
Stringel 1989 (91)	90 (100%)	Retrospective case series	3 months–3 years	Post-op retching in 12 (mostly self-limiting), delayed gastric emptying in 3 (resolved), GI bleed in 1, granulation in roughly half of de Pezzer tube patients, button-site cellulitis in 4. Majors: adhesion-related small-bowel obstruction in 3, wound infection in 3.	Mixed	Serious
Tarek 2024 (92)	34 (100% severe NI)	Prospective observational	6 months	Site infection 20.5% (7/34), granuloma 14.7% (5/34); 55.8% had no complications. 1 major (pneumoperitoneum, managed conservatively).	Favourable	Serious
Jennuvat 2023 (49)	39 (100%)	Cross-sectional + retrospective	Median PEG feeding 33 months	Early (≤14d): pneumonia 28.2%, infected wound 10.3%, hypersecretion 2.6%. Late: obstruction 23.1%, infected wound 20.5%, granulation 15.4%. 1 major (buried bumper, 2.6%).	Unfavourable	Moderate
Işık & Akbulut 2021 (68)	91 (70, 76.9% NI)	Retrospective, single-centre	6 months–5 years	Minor complications in 37.4% (34/91): hypergranulation 15.4%, tube blockage 6.6%, external leakage 6.6%, tube degradation 4.4%, dislodgement 4.4%. Majors 3.3% (intra-abdominal abscess, buried bumper, peritonitis; one each).	Mixed	Serious
Noviello 2021 (75)	148 (111, 75% NI)	Retrospective review	Variable (6-monthly review)	Minor complications in 41.9% (62/148): granuloma (*n* = 41), wound infection (*n* = 10), peristomal leakage (*n* = 11). Majors in 8.7% (13/148), predominantly infection with dislocation (*n* = 7) and buried bumper (*n* = 5).	Unfavourable	Moderate
Wong 2019 (86)	183 ED presentations (100% CP)	Retrospective population cohort (CP register–ED linkage)	5-year window	Dislodgement accounted for 70% of GT-related ED presentations (105/151), far exceeding leakage (10%), obstruction (3.3%), malfunction (2.7%), site infection (2%), granulation (2%); failed reinsertion 11.3%. Admission required in 9.3% overall, more often for GJ (40%) than GT (7%, *p* = 0.001).	Unfavourable	Moderate
Kaur 2019 (93)	95 (≈73, ≈77% NI)	Retrospective, single-centre	Not reported	Local infection 33% (32/95), accidental removal in 12. Majors: oesophageal perforation (*n* = 2, conservatively managed), gastric perforation with peritonitis (*n* = 1), duodenal ulceration from distal migration (*n* = 3).	Unfavourable	Not appraised
Lertudomphonwanit 2019 (59)	201 (168, 83.6% NI)	Retrospective cohort	30 days (complications)	≥1 early complication in 20% (40/201; 43 events), dominated by wound infection (17%, mostly antibiotics/wound care alone). Majors uncommon (1.5%): peritonitis (*n* = 2), infected VP shunt (*n* = 1).	Favourable	Serious
Romano 2016 (130)	56 (100%)	Prospective	6 months	Constipation 33.3%, RTI 37.5%. No majors.	Mixed	Not appraised
Wales 2002 (84)	111	Retrospective cohort (GJ vs. FG comparator)	GJ mean 3.11yr; FG mean 5.71yr	GJ vs. FG (fundoplication + GT): any minor complication trended higher with GJ (87% vs. 73%, *p* = 0.09, NS); dislodgement (66.7% vs. 36.5%, *p* = 0.002) and breakage (43.8% vs. 22.2%, *p* = 0.02) sig. more frequent with GJ; retching sig. more common with FG (36.5% vs. 16.7%, *p* = 0.03). Majors trended higher with FG (65.1% vs. 47.9%, *p* = 0.08, NS); FG-specific complications (wrap failure 14.3%; other majors 9.5%, *p* = 0.04) a significant added risk.	Mixed	Serious
Smith 1999 (9)	40 interviewed (100% CP)	Population-based retrospective cohort + caregiver interview	Mean age at FU 8.5 years	Minor complications in 95% (38/40): diarrhoea, constipation, tube obstruction, site infection, accidental dislodgement, leakage; button-valve problems in 84% of button users (sig. more than other tube types). Majors in 20% (8/40): volvulus, prolapse, bowel obstruction, ulceration, GI bleed, peritonitis.	Unfavourable	Moderate
Viswanath 2010 (61)	137 (100% NI)	Retrospective cohort (three sequential management eras)	Median 3.1 years	Wound infection/hypertrophic granulation: 9/82 (11.0%) gastrostomy-alone vs. 2/60 (3.3%) fundoplication group. Gas-bloat syndrome 5/60 (8.3%, fundoplication group only). Postoperative pneumonia 1/82 (1.2%) vs. 3/60 (5.0%).	Mixed	Serious
Clayton 2024^b^ (88)	21 record-cohort + 26 survey-cohort (100% NI, Dravet syndrome)	Retrospective record review (2 centres) + separate UK-wide caregiver survey	Mean 3.8y (paediatric record); 6.1y (survey)	Long-term complications: paediatric record subgroup 3/10 (30%); combined record cohort 7/21 (33%); survey cohort 19/26 (73%)—peristomal infection requiring antibiotics most common (6 record-cohort, 17 survey). One gastrostomy removed for peristomal pain/leaking.	Mixed	Moderate
AlNamshan 2019 (55)	178 (100% NI)	Retrospective review/cohort (1999–2014)	Early complications (timeframe not further specified)	Early GT/tube-related complications reported as 61% of the cohort overall; individual event rates as extracted (site infection 71.3%, leakage 36.5%, dislodgement 30.3%, granuloma 14.6%, bleeding 2.8%) sum higher than the stated 61% total—an internal inconsistency in the source that could not be resolved from the extraction alone.	Unfavourable	Serious
O'Loughlin 2013 (43)	122 (100% NI)	Retrospective cohort + cross-sectional caregiver questionnaire	Not specified	Mild-to-moderate complications in 36/122 (30%): slipped wrap/hiatus hernia, gagging/retching in 25 (20%), dumping in 4 (3%), gas-bloat in a further subset.	Mixed	Moderate
Cameron 1995 (62)	63 (100% NI)	Retrospective cohort (PEG vs. surgical gastrostomy comparator)	Not specified	PEG group: skin erosion/infection (*n* = 4), intraperitoneal leak (*n* = 2), vomiting (*n* = 1), early dislodgement (*n* = 1). Surgical-gastrostomy group: skin erosion/infection (*n* = 4), plus further events not fully itemised in extraction.	Mixed	Serious
Samuel 2016 (45)	32 (100% NI)	Retrospective study	3 months	Local cellulitis in 23%; 66% had no reported complications.	Favourable	High
Chao 2009 (46)	46 (39 completed; 100% NI)	Retrospective cohort	Not specified	Minor complications (superficial leakage, granulomata) in 33%.	Mixed	Serious
Breton 2016 (129)	164 NI (of 259 total cohort)	Retrospective cohort	Not specified	23 complications recorded across the whole cohort; the source specifies all 23 occurred among the 164 NI patients (none in the 95 non-NI patients), giving an NI-specific rate of approximately 14% (23/164). Complication types not itemised in the extraction.	Mixed	Not appraised

RTI, respiratory tract infection; GOR, gastro-oesophageal reflux; GT, gastrostomy(-fed); NG, nasogastric; NS, not significant.

a Franken 2020a is the larger, later cross-sectional analysis (*n* = 150) of an overlapping Netherlands cohort; the earlier interim report from the same group (Franken 2019, *n* = 50) is not shown separately for this outcome.

b Clayton 2024 reports two potentially overlapping caregiver/patient subgroups (a case-note record review and an anonymised UK-wide survey); the authors state some individuals may appear in both and the overlap cannot be quantified; the two counts should therefore not be treated as independent.

**Table 6:** Major complications after gastrostomy.

Author/Year	N (NI)	Design	Follow-up	Finding	Direction	Risk of bias
Zhang 2025 (27)	84 (84, 100%)	Randomised controlled trial (PEG vs. NG)	28 days	Gastrointestinal bleeding in 0/42 (0%) PEG versus 6/42 (14.3%) NG controls (*p* = 0.026). No deaths, perforations or reoperations described.	Favourable	High
Clayton 2024 (88)	21 record/26 survey (100% NI)	Retrospective record review + caregiver survey	Mean 3.4–6.1 years	Periprocedural complications in 0/10 paediatric, 3/21 (14%) combined and 6/26 (23%) survey respondents, including bleeding at the site, pneumonia requiring intravenous antibiotics, infection requiring a 10-day admission and difficulty with placement. Authors estimate a major-complication rate of approximately 9%; no deaths, perforations or reoperations.	Mixed	Moderate/High
Catto-Smith 2006 (35)	98 (100%)	Retrospective cohort	6–14 years	Esophageal tear 1%, gastrocolic fistula 1% (approx. 2% total)	Favorable	Serious
Tarek 2024 (92)	34 (34, 100%)	Prospective observational	6 months	Pneumoperitoneum in 1 (2.9%), managed conservatively.	Favourable (low rate)	Serious
Jennuvat 2023 (49)	39 (39, 100%)	Cross-sectional + retrospective pre/post	Median 33 months	Buried bumper syndrome in 1 (2.6%).	Favourable (low rate)	Moderate/High
Saleem 2023 (73)	40 (40, 100%)	Prospective cohort	6 months	No major complications reported.	Favourable	Serious
Jadi 2023 (71)	63 (NI subgroup)	Retrospective chart review	12 months	1 patient (1.6%) had a major complication involving organ injury—bowel perforation occurring during conversion of the gastrostomy to a gastrojejunal tube.	Favourable (low rate)	Serious
Dreznik 2022 (80)	345 (158, 45.7%)	Retrospective cohort	Median 5 years	Intra-abdominal collection in 1 (0.3%) and redo fundoplication for a slipped Nissen in 2 (0.6%). Of 93 deaths (26.9%), none were related to the surgical procedure.	Favourable (low rate)	Serious
Civan 2021 (120)	41 (NI cohort)	Retrospective observational (abstract only)	Not reported	No serious complications reported. ^b^Abstract only—full text unavailable, so absence of reporting cannot be distinguished from absence of events.	Favourable (low certainty)	Serious
Guillén Redondo 2021 (82)	207 (153, 73.9%)	Retrospective cohort	Mean 40.6 months (median 27)	Among 53 primary fundoplications, 5 (9%) had a major complication: gastric perforation 2, gastro-oesophageal junction stenosis 1, dumping syndrome 2.	Unfavourable (fundoplication-specific)	Serious
Ulman 2021 (101)	109 (109, 100%)	Retrospective cohort	Median > 2 years	2 patients (1.8%) had recurrent reflux after Nissen fundoplication—hiatal hernia in 1 and wrap disruption in 1—both requiring reoperation.	Favourable (low rate)	Moderate
Sakamoto 2021 (69)	89 (82 analysed, 100% NI)	Retrospective cohort	Median 1,770 days (long-term arm)	Major complications occurred only in the short term: ileus in 1 (1.4%) and peritonitis in 1 (1.4%).	Favourable (low rate)	Moderate
Wadman 2021 (99)	146 (23 gastrostomy)	Retrospective chart review	Median 4 years	1 of the 23 gastrostomy patients (4.3%) had post-operative sepsis. No gastrostomy was reversed.	Favourable	Moderate/High
Zambaiti 2021 (100)	145 (145, 100%)	Retrospective cohort	Median 4 years (1–12)	Short-term (<30 days) major complications in 13 (9%): respiratory distress 3, systemic infection/fever 2, bleeding 1, peritonitis/bowel obstruction 1, organ damage 1, tube malfunction or displacement 5. Long-term (>30 days) in 22 (15%): tube malfunction or displacement 11, respiratory distress 5, peritonitis/bowel obstruction 4, systemic infection 2.	Unfavourable (cumulative 24%)	Serious
Işık & Akbulut 2021 (68)	91 (70, 76.9%)	Retrospective single-centre	6 months–5 years (mean 2.34)	3 major complications (3.3%): intra-abdominal abscess 1, buried bumper syndrome 1, peritonitis 1.	Favourable (low rate)	Serious
Noviello 2021 (75)	148 (111, 75%)	Retrospective review	Not reported (6-monthly review)	13 major complications (8.7%): infection with gastrostomy dislocation 7, buried bumper syndrome 5, gastrocolic fistula 1.	Mixed	Moderate
Demirbaş 2021 (44)	20 (20, 100%)	Prospective study	Mean 16.5 months	No procedure-related complications reported.	Favourable	Serious
Suh 2020 (7)	18 (18, 100%)	Retrospective cohort	Median 50.4 months	Transverse colonic perforation in 1 patient (5.6%) after the pull-through method, requiring surgical re-insertion.	Unfavourable (colonic injury)	Moderate
Franken 2020a (47)	150 (≈74% NI)	Cross-sectional	Median 4.0 years	4 patients (3.4%) required conversion to a jejunal tube because they did not tolerate gastric feeding.	Favourable (low rate)	Moderate/High
Franken 2020b (47)	50 (36, 72%)	Prospective longitudinal cohort (pre/post)	Median 3.9 months	1 gastrostomy was removed for recurrent leakage complicated by persistent local infection.	Favourable	Serious
Wong 2019 (86)	183 ED presentations from 87 children (100% CP)	Retrospective population cohort (CP register–ED linkage)	5-year presentation window	17 of 183 presentations (9.3%) resulted in admission, all for tube complications requiring reinsertion or interventional radiology; 40% of gastrojejunal-tube presentations were admitted versus 7% of gastrostomy-tube presentations (*p* = 0.001). ^b^Denominator is presentations, not patients.	Unfavourable (gastrojejunal tubes)	Moderate
Kaur 2019 (93)	95 (≈73, ≈77%)	Retrospective single-centre	Not reported	Oesophageal perforation in 2 (intra-procedural, managed conservatively), stomach perforation with peritonitis in 1, and duodenal ulcer from distal migration in 3.	Mixed	Not appraised
Nelson 2019 (57)	948 (948, 100%)	Population-based exposure-crossover study	Up to 5 years	Feeding-tube revision required in 132 children (13.9%) within 5 years.	Mixed	Low/Moderate
AlNamshan 2019 (55)	178 (178, 100%)	Retrospective cohort	Median 36–44 months	Early: oesophageal perforation 2 (1.1%), intraperitoneal leakage 2 (1.1%), wound dehiscence 4 (2.2%), pleural effusion 1 (0.6%). Late: intestinal obstruction requiring laparotomy/adhesiolysis 3 (1.7%), wrap failure requiring redo fundoplication 3 (1.7%).	Mixed (individually low; ≈8% cumulative)	Serious
Lertudomphonwanit 2019 (59)	201 (168, 83.6%)	Retrospective cohort	30 days (complications)	3/201 (1.5%) major complications: peritonitis 2 (1%, one requiring surgical correction and one managed with antibiotics) and infected ventriculoperitoneal shunt 1 (0.5%, requiring shunt removal and intravenous antibiotics).	Favourable (low rate)	Serious
Samuel 2016 (45)	32 (32, 100%)	Retrospective study (pre/post)	3 months	1 patient was switched to a gastrojejunal tube because of vomiting and aspiration risk; no other major events.	Favourable	High
Stewart 2017 (85)	535 reviewed (29 with BBS; 26/29 with neurodevelopmental comorbidity)	Retrospective case-note review	Mean 1,025 ± 634 days to buried bumper syndrome	Buried bumper syndrome in 29/535 (5.4%), 31 episodes; 25/29 (86%) required laparotomy. By tube type the syndrome affected 16 PEG-J versus 13 PEG patients, implying approximately 27% of PEG-J versus 2.7% of PEG placements. Second or subsequent tube insertion was a significant risk factor (*p* < 0.0004 PEG, *p* < 0.0001 PEG-J). ^b^NI denominator unknown—not NI-separable.	Unfavourable (buried bumper, jejunal extension)	Moderate
Breton 2016 (129)	259 (164, 63%)	Retrospective cohort (pre/post)	Median 3.2 years	Field reports new-onset reflux in 25 (10%) and worsening in 28, with 81% of affected children being neurologically impaired—a gastro-oesophageal rather than a major surgical complication.	Not assessable (outcome mis-assigned)	Not appraised
Lertudomphonwanit 2014 (131)	133 (133, 100%)	Retrospective cohort	Not reported	6.8% required fundoplication for gastro-oesophageal reflux disease. ^a^Overlapping Thai cohort with Lertudomphonwanit 2019 (2001–2013).	Mixed (reoperation rate)	Not appraised
Franken 2015 (34)	300 (225, 75%)	Retrospective cohort	Median 2.63 years	Major complications in 6 patients (2.0%): post-operative dehiscence of the stomach wall 4 (1.3%), intra-operative bleeding 1 (0.3%), omental herniation 1 (0.3%). ^a^Identical figures to Franken 2013 (same institution, same 2004–2011 period)—counted once.	Favourable (low rate)	
Backman 2014 (122)	180 (61 with unambiguous CP)	Prospective consecutive series (before-after)	6 months	All 180 children tolerated the procedure; no major complications were named or quantified, and the mixed population is not separable.	Not assessable	Moderate
Åvitsland 2013 (81)	58 (39, 67%)	Prospective cohort	18 months	5 deaths during the study period, all attributable to underlying disease; none procedure-related.	Favourable (no procedure-related deaths)	Moderate
O'Loughlin 2013 (43)	122 (122, 100%)	Retrospective cohort + questionnaire	Median 77 months	Serious surgical complications in 12/122 (10%): redo fundoplication 3, gastropexy 1, oesophagojejunostomy with gastric disconnection 1, wound dehiscence 1, post-operative pneumonia 1, post-operative pain 1, perforated duodenum 1.	Unfavourable	Moderate/High
Fascetti-Leon 2012 (76)	239 (189, 79%)	Prospective cohort	24 months	Gastrocolic fistula in 4 (1.7%) at 1 month, 1 (0.4%) at 3 months and 1 (0.4%) at 6 months; haemorrhage 1 (0.4%) at 1 month; peritonitis 1 (0.4%) at 6 months; tube displacement 3 (1.2%) at 1 month and 1 (0.4%) at 3 months.	Favourable (low rate; notable gastrocolic fistula burden)	Moderate
Mahant 2011 (107)	16 caregivers (16, 100%)	Qualitative interview study	≥1 year	Gastrojejunal-tube intussusception, tube dislodgement and blockage described narratively without rates.	Not assessable (qualitative)	Low
Lefton-Greif 2011 (48)	28 (28, 100%)	Retrospective review	Mean 4.11 years (median 3.58)	3 patients (11%) died within 30 days of gastrostomy placement, from pneumonia, pneumothorax and respiratory failure.	Unfavourable (high 30-day mortality)	Moderate
Park 2011 (78)	32 (32, 100%)	Retrospective cohort	Mean 12.2 months	Transient pneumoperitoneum 3 (9.4%), paralytic ileus 3 (9.4%), pneumoperitoneum with pneumomediastinum 1 (3.1%), atelectasis 1 (3.1%), and gastrocolic fistula requiring laparotomy 1 (3.1%).	Mixed (frequent but largely self-limiting)	Serious
Kawahara 2010 (97)	54 (54, 100%)	Retrospective cohort	Median 4 years	1 patient (1.9%) required pharyngotracheal separation for respiratory distress and repeated aspiration pneumonia 15 months post-procedure; 1 (1.9%) required continuous gastrojejunal feeding for repeated emesis and intractable diarrhoea.	Favourable (low rate)	Moderate
Vernon-Roberts 2010 (52)	14 (14, 100%)	Prospective longitudinal study	6 months	3 participants (21.4%) died of underlying disease; no complications were directly attributed to the surgery.	Favourable (no procedure-related complications)	Not appraised
Viswanath 2010 (61)	137 (137, 100%)	Retrospective cohort across three protocols	Median 3.1 years	Complication rates were significantly higher after gastrostomy with fundoplication (18/60, 30%) than after gastrostomy alone (12/82, 14.6%), *p* = 0.036. Early: gastric perforation resulting in death 1/60 (1.7%), leakage with peritonitis 1/82 (1.2%), mediastinal collection 1/60 (1.7%). Late: adhesive intestinal obstruction 2/60 (3.3%), failed fundoplication requiring redo surgery 3/60 (5%), slipped catheter requiring exploration 1/82 (1.2%), food bolus obstruction 1/60 (1.7%).	Unfavourable (fundoplication)	
Sathesh-Kumar 2009 (70)	76 (≈76% NI)	Cohort study	Median 52 months	1 patient (1.3%) developed gastric volvulus requiring laparotomy within 30 days of the procedure.	Favourable (low rate)	Moderate/High
Chao 2009 (46)	46 (39 completed)	Retrospective cohort (pre/post)	Mean 25 months	8 patients required surgery for pyloric obstruction, adhesive intestinal obstruction, wound infection, gastrostomy leakage with peritonitis, or a broken or displaced tube.	Mixed	Serious
Toporowska-Kowalska 2008 (134)	14 (14, 100%)	Prospective single-arm cohort	Not reported	Unspecified technical complications prevented PEG insertion in 1 of 14; no complication types or rates reported.	Not assessable	Not appraised
Craig 2006 (124)	76 (76, 100%)	Prospective cohort	6 months post-operative	13 children (17%) had at least one major complication: internal fistula 10.3%, haemorrhage 8.1%, redo Nissen fundoplication 9%, migration 1.4%, adhesions 1.4%.	Unfavourable	Moderate
Sullivan 2006 (11)	57 (57, 100%)	Prospective longitudinal cohort	6 and 12 months	No major complications recorded. ^a^Same Oxford cohort as Sullivan 2005.	Favourable	Serious
Vajsar 2006 (58)	12 (12, 100% SMA type 1)	Retrospective case series	30 days (pre-specified)	Pre-specified major complications within 30 days: aspiration pneumonia 5/12 (41.6%), respiratory failure requiring PICU admission 4/12 (33%), death 2/12 (16.7%). Co-occurrence within individuals is not reported, so the number of children with at least one major complication cannot be derived.	Unfavourable (highest reported rate)	Not appraised
Sullivan 2005 (33)	57 (57, 100%)	Prospective longitudinal cohort	12 months	1 patient (2%) developed severe gastric leakage, peritonitis, skin erythema and ulceration requiring stoma closure, with a new gastrostomy placed 18 months later. ^a^Same Oxford cohort as Sullivan 2006.	Favourable (low rate)	Serious
Wales 2002 (84)	111 (111, 100%)	Retrospective cohort (fundoplication + gastrostomy vs. gastrojejunal tube)	Mean 3.1 years (GJ)/5.7 years (FG)	Any major complication: GJ 47.9% (23/48) versus FG 65.1% (41/63), *p* = 0.08. Intussusception/adhesive bowel obstruction GJ 20.8% vs. FG 7.9% (*p* = 0.06); perforation at insertion GJ 2.1%; bleeding GJ 4.2% vs. FG 3.2%; peritonitis GJ 0% vs. FG 3.2%; deep wound infection GJ 4.2% vs. FG 3.2%; wrap failure (FG only) 14.3%; other major FG-specific events 9.5% vs. 0% (*p* = 0.04), including antibiotic-associated colitis, line sepsis, stress ulceration, perforated duodenum and fascial dehiscence.	Unfavourable (both arms; broad definition)	Serious
Seguy 2002 (37)	12 (12, 100%)	Retrospective cohort	1 year	No major complications reported.	Favourable	Serious
Puntis 2000 (77)	29 (29, 100%)	Retrospective cohort	Median 2.6 years	Technical failure from colonic interposition in 2 (6.9%) necessitating open gastrostomy; asymptomatic gastrocolic fistula in 1 (3.4%) leading to tube removal and reversion to nasogastric feeding; post-operative ventilatory support for 2–5 days in 3 (10.3%).	Mixed (colonic injury and technical failure)	Serious
Smith 1999 (9)	40 interviewed (100% CP)	Population-based retrospective cohort + caregiver interview	Mean 8.5 years	Major complications in 8/40 (20%): volvulus, prolapse, bowel obstruction, ulceration, gastrointestinal bleeding and peritonitis (not individually quantified). No deaths were tube-feeding related.	Unfavourable	Moderate/High
Brant 1999 (13)	20 (20, 100%)	Prospective cohort	Mean 5.9 months	Pneumoperitoneum in 1 (5%).	Favourable (low rate)	Serious
Khattak 1998 (79)	120 (52, 43%)	Retrospective case series	Mean 26.6 months	51 major complications in 21/120 patients (17.5%): reflux requiring an antireflux procedure 17, stomal problems 9, peritonitis 8, failed attempt 5, gastrocolocutaneous fistula 4, intestinal obstruction 3, major haemorrhage 3, flange migration 1, and sepsis with death 1. ^b^Whole cohort; not NI-separable.	Unfavourable (not NI-separable)	Moderate
Borowitz 1997 (72)	19 (19, 100%)	Prospective cohort (pre/post)	Mean 20.7 months	No complications: 0 perioperative events, 0 perforations, 0 fistulas.	Favourable	Serious
Koca 2015 (74)	34 (34, 100%)	Retrospective cohort	Mean 19.4 months	Food escape into the peritoneal cavity in 1 (2.9%) and buried bumper syndrome in 1 (2.9%), the latter managed by traction without surgery and replaced with a button PEG.	Favourable (low rate)	Serious
Cameron 1995 (62)	63 (63, 100%)	Retrospective cohort (PEG vs. Stamm gastrostomy)	Mean 23 months	PEG: no major complications. Stamm gastrostomy: 3 events—reoperation for bleeding in 1 and 30-day mortality in 2.	Mixed (favours PEG)	Serious
Ramelli 2007 (56)	31 (31, 100%)	Longitudinal cohort	Mean 5 years	Tube dislodgement in 1 and tube obstruction in 1; no major surgical complications reported.	Favourable	Serious
Stringel 1989 (91)	90 (90, 100%)	Retrospective case series	2 months–3 years	Small bowel obstruction from adhesions in 3 (at 4 days, 5 days and 2 months post-Nissen; 2 lysis, 1 resection) and wound infection in 3. Intra-operative injuries: liver laceration 1, small bowel laceration 1, diaphragm laceration 1.	Unfavourable	Serious
Langer 1988 (83)	107 (107, 100%)	Retrospective cohort (gastrostomy ± antireflux procedure)	Mean ≈ 20 months	More significant complications—bowel obstruction, wrap herniation and gas bloat—occurred only after an antireflux procedure. Gastrostomy + antireflux (*n* = 36): wound infection 2, dehiscence 1, tube dislodgement 1, bowel obstruction 2, gas bloat 1, slipped wrap 2. Antireflux following gastrostomy alone (*n* = 21): bowel obstruction 2, gas bloat 2, slipped wrap 2, tube dislodgement 1.	Unfavourable (antireflux-specific)	Serious
Wesley 1981 (90)	33 (22, 66.7%)	Retrospective case series	Mean 11 months	Fundoplication technical failure requiring reoperation in 2/22 (9%) at 3 weeks and 3 months; small bowel obstruction from adhesions in 2/22 (9%) at 2 and 3 weeks, both resolving after adhesiolysis; post-operative dilatation required in 2/22 (9%).	Unfavourable	Moderate/High

Buried bumper syndrome, colonic or other visceral injury, gastrocolic fistula, peritonitis, major haemorrhage, unplanned reoperation and procedure-related death. GT, gastrostomy; PEG, percutaneous endoscopic gastrostomy; PEG-J, percutaneous endoscopic gastrostomy with jejunal extension; GJ, gastrojejunal; FG, fundoplication with gastrostomy; BBS, buried bumper syndrome; NI, neurological impairment.

a Companion publications reporting the same cohort.

b Data-quality caution, as noted in the cell.

#### 3.2.1 Nutritional outcomes

Weight and weight-based indices improved after gastrostomy in most cohorts reporting them, but the studies with a parallel comparator group did not agree. Fifty-six studies assessed weight, height, BMI, or micronutrient status before and after gastrostomy placement; six used a parallel oral-, nasogastric-, or alternative-feeding comparator instead of a pure pre/post design (24–28), though in one of these the surgical arm received gastrostomy together with Nissen fundoplication. We classified the direction of effect as favourable in 39 studies, mixed in eight, no significant change in five, and not assessable in three lacking a baseline measurement (29–31). No study reported nutritional deterioration. These counts give equal weight to 12-child small case series and to a 24,920-child cohort, and do not incorporate methodological quality: two studies reached low risk of bias, fourteen moderate, nine moderate-to-high, twenty-four serious and two high, with four not appraised. We therefore ordered the synthesis below by study design and risk of bias rather than by how often a favourable classification appeared.

The six comparator-controlled studies split in direction. Day et al. (25), an administrative cohort of 24,920 children and one of two studies at low risk of bias, found that gastrostomy-fed children gained more weight than orally-fed children across every age band studied, and that gastrostomy-fed children with severe cerebral palsy grew taller than orally-fed peers with the same diagnosis. Caselli et al. (24) found greater arm circumference, triceps skinfold and adipose arm area than in orally-fed peers, and no difference on other measures. Vergara et al. (26), a conference abstract, reported weight-percentile improvement in 29 children treated surgically and none in 26 treated non-surgically; gastrostomy and Nissen fundoplication were performed together, so the contribution of gastrostomy alone cannot be separated. Against these, Sullivan (32) found no difference in WAZ from an oral-fed comparator after adjustment for baseline status and GMFCS level (mean difference 0.002, *p* = 1.0), and Wu et al. (28) found a numerically larger weight gain with gastrostomy than with continued nasogastric feeding during inpatient rehabilitation for acquired brain injury that was not significant (5.1 kg vs. 2.9 kg, *p* = 0.2). Both were small, both carried serious risk of bias, and in Wu the groups differed at baseline in nutritional status.

Among the uncontrolled cohorts, the largest datasets pointed the same way. Sullivan et al. (33), a prospective multicentre cohort of 57 children, reported the largest single median WAZ change in this domain: −3.03 at baseline to −1.60 at 12 months (*p* < 0.0001), with catch-up growth in 91% of children. Franken et al. (34), the largest single dataset for this outcome (*n* = 300), reported a similar direction of effect (WLZ improved from −0.98 to −0.26, *p* < 0.0005); a quarter of that cohort had a non-NI diagnosis, including cystic fibrosis, and growth data were not stratified by diagnosis. Both were rated at serious and moderate risk of bias respectively. Catto-Smith (35) observed a mean increase in weight-for-age *z*-score of 1.05 after a median of 6.1 months in a cohort of 98 neurologically impaired children.

Motil et al. (36) found that gastrostomy feeding improved linear growth and lean body mass alongside a shift in body composition toward proportionally higher adiposity, a pattern also seen in Seguy et al. (37) and Franken et al. (34). Arrowsmith (38) similarly noted a shift in body composition, finding that while weight SDS improved significantly (−4.8 to −3.0, *p* < 0.05), percent body fat by skinfold also increased significantly from 10.7% to 16.3%, with no significant catch-up in height (38). Grayeb et al. (39), the longest follow-up in this domain (median 64 months) and the second study at low risk of bias, found persistent iron deficiency in 64% of tested children and iron-deficiency anaemia in 31% despite significant WAZ improvement. Sanders et al. (40) found a graded relationship between timing of gastrostomy and nutritional recovery: children treated earliest after their neurological insult had the fastest weight-gain velocity (3.3 g/kg/day) and the greatest reversal of malnutrition, against 1.5 g/kg/day in those treated latest. Gębora-Kowalska (41) reported a similarly steep trajectory: BMIZ rose from −4.83 at baseline to −2.39 at 12 months (*p* < 0.001), with cachectic children falling from 52% to 19%. Pinheiro et al. (42), the only study in a population defined by congenital Zika syndrome, found significant WAZ improvement but incomplete catch-up in 39% of children. Finbråtten et al. (30), the largest single-diagnosis registry in this review (661 children with cerebral palsy), reports no pre/post comparison but found growth retardation associated with both later gastrostomy insertion and greater GMFCS severity. Neither Sanders nor Finbråtten randomised the timing of placement, and children who received gastrostomy earlier may differ from those treated later in disease severity and clinical indication.

Several further cohorts reported the same direction of effect. O'Loughlin et al. (43), the second-largest single cohort in this domain (*n* = 122), found WAZ improved from −2.57 to −1.65 (*p* < 0.00005). Demirbaş et al. (44) compared gastrostomy alone with gastrostomy plus concurrent Nissen fundoplication; both arms showed significant WAZ and BMIZ improvement. Chae-ri Suh et al. (7) found significant improvement in BMIZ, HAZ, and WAZ within 6 months under a structured paediatric nutrition-team protocol. Samuel (*n* = 32) (45) found WAZ improved from −1.86 to −1.47 at 3 months. Chao et al. (46) found no significant overall change in weight percentile (15th to 17th, *p* = 0.64), though 20% of children caught up by more than 10 percentiles.

Zhang et al. is the only randomised evidence in this domain: 84 children with traumatic brain injury and dysphagia were randomised to PEG or continued nasogastric feeding, and weight, serum protein, albumin, and haemoglobin all rose more in the PEG group by 28 days (*p* < 0.05 for each)**,** although within the control group the weight change itself was not significant (*p* = 0.089). Follow-up was 28 days; nutritional values were presented graphically only, the study was not prospectively registered, and it was rated at high risk of bias. The authors note their findings require confirmation in larger-scale studies.

Six studies found no significant nutritional change; two of these, Sullivan (32) and Wu (28), are the comparator-controlled studies described above. Franken (47) followed patients for four months or less. Lefton-Greif (48) assessed a paired subsample of ten patients. Jennuvat (49), followed to three years, attributed the absence of catch-up growth to low household income. None of these studies had the power to exclude a modest effect, and a null result here does not establish that gastrostomy has none.

Gastrostomy feeding is associated with improvement in weight and weight-based nutritional indices across the majority of included studies. Height gain and micronutrient correction lag behind weight gain in a minority of cohorts. The one comparator-controlled study that adjusted for baseline nutritional status and motor severity found no difference from oral feeding at 12 months, and both comparisons against nasogastric feeding ran for 28 to 43 days only.

#### 3.2.2 Respiratory outcomes

Fifty-six of the included studies reported a respiratory outcome; chest-infection frequency before vs. after gastrostomy, respiratory or aspiration-related hospitalisations, or discrete aspiration events. These records represent approximately fifty-four independent cohorts: Sullivan 2005 and Sullivan (11, 33) report complementary respiratory data from the same 57-child Oxford Feeding Study, and Jacoby and Downs (50, 51) both analyse the same linked Western Australian and New South Wales birth cohort (*n* = 673), so neither pair should be counted twice in any overall count; two further duplicate Covidence records (52, 53) and a duplicated Kehar (54) record were resolved to a single entry each. The overall direction is favourable but considerably less consistent than for nutritional outcomes: twenty-four studies reported a reduction in chest infections, respiratory admissions, or aspiration events after gastrostomy, four found no significant change, eight described a mixed picture, and four reported an unfavourable signal, while sixteen mentioned respiratory outcomes without a baseline comparison, a denominator, or a numerator sufficient to judge direction. Where improvement was quantified it was often substantial: Sullivan et al. (11, 33), which were assessed as having a moderate-to-high risk of bias and serious risk of bias, found that the mean number of chest infections requiring antibiotics fell from 1.8 to 0.9 at twelve months, that admissions for chest infection fell from a mean of 0.5 to 0.09 (*p* = 0.04), and that the proportion of children admitted at least once with a chest infection fell from 26% to 7% (*p* = 0.021), with no child admitted for a chest infection by twelve months. Comparable magnitudes were reported by Chao (46) (moderate risk of bias), AlNamshan (55) (serious risk of bias), Ramelli (56) (serious risk of bias), and Jennuvat (49) (moderate/high risk of bias; pneumonia-related admissions 1.6 to 0.6 per patient, *p* < 0.001; ICU admissions 0.45 to 0.13, *p* = 0.006; reflux- and aspiration-related admissions 0.67 to 0.09 per person-month, *p* < 0.001; 26 of 29 children with a pre-operative history of chest infection had none at review; aspiration-pneumonia admissions median 1 to 0.5 per year, *p* < 0.001).

The countervailing evidence comes from the largest and most methodologically rigorous datasets, all of which are population-linked and comparator-controlled. Lin et al. (12), a lower-risk study with a moderate risk of bias, a 1:2 propensity-score-matched cohort of 2,490 children with neurological impairment and a prior pneumonia admission, found subsequent severe pneumonia hospitalisation in 32% and an increased risk of the composite outcome of death or severe pneumonia after new gastrostomy placement (HR: 1.21), even though mortality alone fell (HR: 0.47); Jacoby et al. (50), assessed at low risk of bias, and Downs et al. (51), assessed at serious risk of bias due to reporting from an abstract, analysing 673 Australian children against their own pre-gastrostomy year, found that lower-respiratory-tract-infection admissions rose in the first post-operative year (IRR: 1.26) and remained elevated at six years or more (IRR: 1.42) despite an overall decline in all-cause admissions; and Nelson et al. (57), also among the higher-quality evidence with a low/moderate risk of bias, an exposure-crossover analysis of 948 children, found reflux-related hospital days and acute health-care use unchanged before and after tube placement (adjusted RR: 0.92, 95% CI: 0.57–1.48). This split is systematic rather than random: the favourable studies are predominantly small, single-centre, at serious or moderate/high risk of bias, and dependent on caregiver recall or clinician-recorded infection counts, whereas the null and unfavourable studies are large, administratively linked, comparator-controlled, and generally carry a lower risk of bias (low to moderate), which suggests that part of the apparent respiratory benefit reflects recall and selection effects rather than a true treatment effect. Mechanistic and high-risk-population data point the same way: Thomson et al. (53), judged to have moderate/high risk of bias, showed that the proportion of reflux events reaching the pharyngeal space rose from 56% to 82% after PEG (*p* = 0.022) with no clinical change in chest-infection frequency, and Vajsar et al. (58) recorded aspiration pneumonia in 5 of 12 and respiratory failure requiring PICU admission in 4 of 12 children with SMA type 1 within thirty days of insertion. In summary, gastrostomy is associated with fewer chest infections and fewer respiratory admissions in most small before-and-after cohorts, though confidence in these findings is limited by widespread moderate to serious risk of bias; conversely, the methodologically stronger studies with lower risk of bias show no reduction—and in some analyses an increase—in pneumonia-related hospitalisation, indicating that gastrostomy should not be expected to abolish aspiration risk, particularly where salivary aspiration or uncontrolled reflux is the dominant mechanism.

#### 3.2.3 Minor complications

Minor complications after gastrostomy were common but rarely severe. Forty-two studies reported infection, granulation tissue, leakage, or dislodgement in sufficient detail for this table. All were observational and none was rated at low risk of bias: 16 carried moderate risk of bias, two moderate/high, 21 serious and one high; two conference abstracts were not formally appraised. We classified the direction of effect as mixed in 21 studies, unfavourable in 13, and favourable in eight. These counts give equal weight to a 239-child prospective multicentre series and to a twenty-child retrospective case series; the synthesis below is therefore ordered by study design and risk of bias rather than by the frequency of any single classification. Reported rates for any minor complication ranged from 20% (complications tracked only to 30 days) (59) to 95% (caregiver interview) (9), a range corresponding to differences in ascertainment method more closely than to differences between populations. AlNamshan et al. (55) reported one of the highest rates in this table (*n* = 178): 71.3% site infection alone, with leakage (36.5%) and dislodgement (30.3%) also frequent, although the cohort-level total reported in that study (61%) is lower than the sum of its individual event rates. Franken et al. (34), the largest cohort in this domain (*n* = 300), reported a comparable overall rate of 73.7% alongside a low major-complication rate of 2.0%. Catto-Smith (35) reported fever in 17%, cellulitis in 7%, and peritonism in 4% of patients. Across studies reporting both categories, minor events exceeded major events by approximately an order of magnitude.

Wong et al. (60) offers a distinct signal drawn from emergency-department data rather than surgical follow-up: tube dislodgement accounted for 70% of gastrostomy-related presentations, and gastrojejunostomy tubes required hospital admission far more often than gastrostomy tubes (40% vs. 7%, *p* = 0.001). Viswanath et al. (61) adds a further nuance to the fundoplication pattern discussed elsewhere in this review: wound infection was more frequent with gastrostomy alone (11.0% vs. 3.3%), while gas-bloat syndrome occurred more often with concurrent fundoplication. Langer et al. reported that bowel obstruction, wrap herniation and gas-bloat occurred only where an additional anti-reflux procedure had been performed, and Cameron et al. (62) found skin erosion or infection in four children in each of the percutaneous endoscopic and surgical groups. Rates drawn from cohorts undergoing concurrent fundoplication or gastrojejunal extension are therefore not directly comparable with those from gastrostomy alone. Minor complications after paediatric gastrostomy are frequent but low acuity overall, and tube type, concurrent anti-reflux surgery, complication definition, ascertainment method, and follow-up duration account for most of the variability in rates reported across studies.

Study-level data for this domain are presented in Table 5.

#### 3.2.4 Major complications

Fifty-nine of the included studies reported major gastrostomy- or fundoplication-related complications; peritonitis, visceral or colonic injury, gastrocolic fistula, buried bumper syndrome, major haemorrhage, reoperation, or procedure-related death. Eight further studies were excluded from this table after a data-quality check: Grayeb et al. (39) (low risk of bias) and Aumar et al. (63) (low/moderate risk of bias), whose authors explicitly did not report complication data; Benoit (64) (serious risk of bias), in which no complications of any kind were recorded; and Petersen (65), Kehar (54) (serious risk of bias) (54), Nakao (66) and Arnbjörnsson (67) (serious risk of bias) (65, 66), whose major-complication fields contain only minor events, such as granulation tissue, peristomal erythema, leakage and tube dislodgement, that belong in the minor-complication synthesis.

Across the 59 usable studies, the dominant pattern is that serious events are uncommon but not negligible: twenty-nine studies reported a low or nil major-complication rate, sixteen reported a burden high enough to be classified as unfavourable, ten described a mixed picture, and four reported major complications only qualitatively, in a mis-assigned field, or in a form that could not be separated from a mixed population. Among studies confined to gastrostomy alone, rates cluster tightly between 0% and 3.5%: Franken (34) (*n* = 300) 2.0% (moderate risk of bias), Lertudomphonwanit (59) (*n* = 201) 1.5% at 30 days (serious risk of bias), Işık and Akbulut (68) (*n* = 91) 3.3% (serious risk of bias), Sakamoto (69) 2.8% (moderate risk of bias), Sathesh-Kumar (70) 1.3% (moderate/high risk of bias), Jadi (71) 1.6% (serious risk of bias), and 0% in Borowitz (72) (serious risk of bias), Seguy (37) (serious risk of bias), Saleem (73) (serious risk of bias), Demirbaş (44) (serious risk of bias) and Sullivan (32) (serious risk of bias).

The specific events named in the review question are individually rare across observational literature. Catto-Smith (35) recorded isolated cases of esophageal tear (1%) and late gastrocolic fistula (1%). Buried bumper syndrome was reported by Koca (74) (1/34, 2.9%, managed without surgery, serious risk of bias), Jennuvat (49) (1/39, 2.6%, moderate/high risk of bias), Işık and Akbulut (68) (1/91, serious risk of bias) and Noviello (75)(5/148, 3.4%, moderate risk of bias); colonic injury or gastrocolic fistula by Suh (7) (transverse colonic perforation 1/18, 5.6%, requiring surgical re-insertion, moderate risk of bias), Fascetti-Leon (76) (6/239 cumulative, 2.5%, moderate risk of bias), Puntis (77) (colonic interposition preventing PEG placement in 2/29 and asymptomatic gastrocolic fistula in 1/29, serious risk of bias), Park (78) (1/32, 3.1%, requiring laparotomy, serious risk of bias), Noviello (75) (1/148, moderate risk of bias) and Khattak (79) (4/120, moderate risk of bias); peritonitis by Lertudomphonwanit (59) (2/201, 1%, serious risk of bias), Sakamoto (69) (1.4%, moderate risk of bias), Işık and Akbulut (68) (1/91, serious risk of bias), Sullivan (33) (1/57, 2%, moderate risk of bias), Viswanath (61) (1/82, 1.2%, serious risk of bias) and Khattak (79) (8/120, moderate risk of bias); and procedure-related death by Viswanath (61) (one gastric perforation, serious risk of bias), Cameron (62) (two 30-day deaths, both in the Stamm gastrostomy arm, serious risk of bias) and Khattak (79) (one sepsis-related death, moderate risk of bias). Meanwhile, several large cohorts—Grayeb (39) (low risk of bias, none of 94 childhood deaths attributed to the procedure), Dreznik (80) (serious risk of bias, 93 deaths, none procedure-related), Åvitsland (81) (moderate risk of bias) and Smith (9) (moderate/high risk of bias) which explicitly documented no procedure-attributable mortality.

The clearest and most consistent signal in this domain is that excess serious morbidity is driven by concurrent antireflux surgery and by jejunal extension rather than by gastrostomy itself. Viswanath et al. (61) (serious risk of bias) found post-operative complication rates significantly higher after gastrostomy with fundoplication than after gastrostomy alone (18/60, 30% vs. 12/82, 14.6%; *p* = 0.036), including the series' only procedure-related death; Guillén Redondo et al. (82), a comparative study at serious risk of bias, reported gastric perforation, gastro-oesophageal junction stenosis or dumping in 5 of 53 primary fundoplications (9%); Langer (83) (serious risk of bias) observed that bowel obstruction, wrap herniation and gas bloat occurred exclusively after an antireflux procedure; and Wales et al. (84), a comparatively detailed dataset despite serious risk of bias, recorded any major complication in 65.1% of fundoplication-plus-gastrostomy vs. 47.9% of gastrojejunal patients (*p* = 0.08), with fundoplication-specific events (wrap failure 14.3%; other major events 9.5% vs. 0%, *p* = 0.04) accounting for the difference while gastrojejunal tubes carried their own excess of intussusception and adhesive obstruction (20.8% vs. 7.9%, *p* = 0.06).

The same device effect appears in Stewart et al. (85) (moderate risk of bias), the largest dedicated buried-bumper series (29/535, 5.4%; 86% requiring laparotomy; mean 1,025 days to presentation), in which the syndrome affected approximately 27% of PEG-J vs. 2.7% of PEG placements, and in Wong et al. (86, 87) (low/moderate risk of bias), where 40% of gastrojejunal-tube emergency presentations required admission vs. 7% for gastrostomy tubes (*p* = 0.001). The highest reported rates belong to specific high-risk populations and broad pre-specified definitions rather than to technique: Vajsar et al. (58) recorded aspiration pneumonia in 5 of 12, respiratory failure requiring PICU admission in 4 of 12 and death in 2 of 12 children with SMA type 1 within thirty days, and Lefton-Greif et al. (48) (moderate risk of bias) reported 30-day mortality of 11% (3/28) from pneumonia, pneumothorax and respiratory failure, which are figures that reflect the fragility of the children selected for the procedure more than the procedure itself.

In summary, major complications after paediatric gastrostomy for neurological impairment are uncommon, affecting roughly 0%–3% of children when the procedure is performed alone, with buried bumper syndrome, colonic injury and peritonitis each reported in fewer than 1 in 20. Although confidence in precise rates is limited by the predominance of studies at serious or moderate risk of bias, the finding that serious-morbidity burden rises several-fold with concurrent antireflux procedures or jejunal extensions is consistent across study types, and the highest mortality figures come from cohorts of profoundly fragile children in whom deaths were consistently attributed to underlying disease rather than to the gastrostomy.

Study-level data for this domain are presented in Table 6.

#### 3.2.5 Neurodevelopmental outcomes

The primary outcome domain of this review is lacking, where only eleven studies reported anything at all bearing on neurodevelopmental function, and of these only one applied a standardised instrument. We classified the direction of effect as favourable in three studies, all of which describe development qualitatively and none of which measured it; as no difference in three; and as not assessable in five, in which the domain was either not measured or reported in a form from which no numerator could be extracted. No study reported an unfavourable neurodevelopmental effect, and none reported cognition, gross or fine motor function, communication, adaptive behaviour or school participation using a validated developmental instrument before and after gastrostomy. For every other included study, the neurodevelopmental field of the extraction form was completed as not reported or left blank. Importantly, none of the studies contributing to this domain was at low risk of bias. Among the studies with the lowest risk-of-bias ratings, Sanders et al. (40)was rated moderate, while Clayton et al. (88), Tawfik et al. (89), and Wesley et al. (90) were rated moderate/high. Even within this comparatively stronger subset, neurodevelopmental outcomes were not assessed using validated instruments.

The single exception is instructive. Wu et al. applied the WeeFIM Cognition scale at admission and discharge to 74 children undergoing inpatient rehabilitation after acquired brain injury, comparing those who received a new gastrostomy with those who continued nasogastric feeding. Cognitive function improved substantially across the whole cohort, from 12.2 ± 6.5 to 19.4 ± 7.9 (*p* < 0.001), but the improvement was unrelated to the feeding route: tube type had no effect on the subtotal at either admission or discharge (*p* > 0.8), both arms improved within the nil-by-mouth subgroup, and every between-group comparison was non-significant. Eating function, measured on the IDDSI Functional Diet Scale, improved from a median of 0 to 8 (*p* < 0.001) with no difference between arms at discharge. The study is small, non-randomised, confined to a single inpatient rehabilitation admission of mean 43 days, and reports its cognition test statistic inconsistently between the Results and the Abstract, and was rated at serious risk of bias. Accordingly, although cognitive recovery occurred during follow-up, the study did not demonstrate a difference in recovery attributable to gastrostomy compared with continued nasogastric feeding.

The remaining ten studies measured proxies. Four reported seizure control, which is the most frequently recorded neurological outcome in this literature and the least consistently measured. Clayton et al. (88) documented improved seizure control in 11 of 21 gastrostomy recipients (52%) from case-note review of a Dravet syndrome cohort, without a seizure diary, and separately asked caregivers to report perceived change in convulsive seizures, cognition and walking; the survey proportions appear only as pie charts and are not numerically extractable. The study was rated at moderate/high risk of bias. Samuel (45) found seizure frequency unchanged in 76% of 21 children with epilepsy, improved in three and worsened in two and was rated at high risk of bias. Tawfik et al. (89) found epilepsy control improved in three, worse in three and unchanged in 14, not statistically significant; this study was rated at moderate/high risk of bias. Stringel (91) reported the most favourable seizure result in the review, with 73% of 35 children under neurology follow-up showing a significant reduction in seizure activity and 90% a reduced anticonvulsant dose, but the procedure in that series was a Nissen fundoplication and no standardised measure was applied. It was also rated at serious risk of bias. Sanders et al. (40) and Wesley et al. (90) describe developmental progress qualitatively, in the language of good outcomes and improved neurologic growth, without measurement. Sanders et al. was rated at moderate risk of bias, the lowest rating among studies in this domain, while Wesley et al. was rated moderate/high; thus, even the comparatively stronger evidence for favourable developmental change relied on qualitative, non-standardised assessment. Zhang et al. (27), Tarek et al. (92) and Kaur et al. (93) measured swallowing function or recovery of oral feeding, which are outcomes of the feeding route rather than of development. Benoit et al. (64) recorded developmental delay in 93% of a subgroup as a baseline characteristic, with no outcome applied.

Overall, studies relying on narrative or caregiver-reported assessments tended to describe neurodevelopmental improvement, whereas the only study using a standardised cognitive measure found no improvement attributable specifically to gastrostomy compared with continued nasogastric feeding. Given the small number of studies, absence of low-risk evidence, and lack of standardised neurodevelopmental assessment, the available evidence is insufficient to determine whether gastrostomy has a favourable, unfavourable, or neutral effect on neurodevelopment.

The question this review set out to answer, and the question families most often ask when a gastrostomy is proposed, therefore cannot be answered from the existing literature. This is the clearest finding of the review.

Study-level data for this domain are presented in Table 7.

**Table 7:** Neurodevelopmental outcomes after gastrostomy.

Author/Year	N (NI)	Design	Follow-up	Instrument used	Finding	Direction	Risk of bias
Wu 2026 (28)	74 (74, 100% acquired brain injury)	Retrospective cohort (new gastrostomy vs. continued NG at inpatient rehabilitation admission)	Inpatient stay only, mean 43.2 days	WeeFIM Cognition (normalised subtotal, 5–35) and the IDDSI Functional Diet Scale (0–8)	WeeFIM Cognition improved across the whole cohort from 12.2 ± 6.5 at admission to 19.4 ± 7.9 at discharge (*p* < 0.001), but tube type had no effect on the subtotal at either admission or discharge (*p* > 0.8). Within the nil-by-mouth subgroup both arms improved (NG 9.7 to 11.7, *p* = 0.002; gastrostomy 16.5 to 19.4, *p* < 0.001), while every between-group comparison was non-significant. Eating function improved from a median of 0 to 8 across the cohort (*p* < 0.001), with no difference between arms at discharge. The test statistic for the cognition result is reported inconsistently between the Results (*t* = 11.5) and the Abstract (*t* = 9.25).	No difference (attributable to feeding route)	Serious
Clayton 2024 (88)	21 record cohort/26 survey cohort (100% NI, Dravet syndrome)	Retrospective record review (2 centres) + separate UK-wide caregiver survey	Mean 3.4–6.1 years	None validated; case-note review of seizure control, plus caregiver-reported perception	Seizure control improved in 11/21 (52%) of gastrostomy recipients on case-note review, children and adults combined, with no formal seizure diary. Survey caregivers reported change in convulsive seizures, cognition and walking after gastrostomy, mostly improvement or no difference, but the proportions appear only in pie charts and are not numerically extractable.	Not assessable (no validated instrument; figures not extractable)	Moderate/High
Samuel 2016 (45)	32 (32, 100%)	Retrospective study	3 months	None validated; seizure frequency from records	Among 21 children with epilepsy, seizure frequency was unchanged in 76%, improved in 3 and worsened in 2.	No difference	High
Tawfik 1997 (89)	29 (29, 100%)	Retrospective cohort + structured caregiver questionnaire	1–5 years	None validated; caregiver-rated epilepsy control	Epilepsy control was improved in 3, worse in 3 and unchanged in 14; not statistically significant overall or in either subgroup.	No difference	Moderate/High
Stringel 1989 (91)	90 (90, 100%)	Retrospective case series	3 months–3 years	None validated; neurology follow-up records and caregiver-reported milestones	Among 35 children with neurology follow-up, 73% had a significant reduction in seizure activity after Nissen fundoplication and 90% had their anticonvulsant dose reduced. Developmental milestones were described qualitatively as generally improved and in some cases spectacular, with no standardised instrument.	Favourable (uncontrolled, concurrent fundoplication, no measurement)	Serious
Sanders 1990 (40)	51 (51, 100% CP)	Retrospective before-after cohort	Mean 2.4 years	None; caregiver report	Significant developmental progress described qualitatively; no standardised measure applied.	Favourable (qualitative, low certainty)	Moderate
Wesley 1981 (90)	33 (22, 66.7%)	Retrospective case series	Mean 11 months	None	Improved neurologic growth and development described in many of the children classified as having a good outcome; not measured or quantified.	Favourable (qualitative, low certainty)	Moderate/High
Zhang 2025 (27)	84 (84, 100%)	Randomised controlled trial (PEG vs. indwelling NG)	28 days	Water Swallow Test; Dysphagia Disorders Survey	Swallowing function was measured and reported, but no cognitive or neurodevelopmental outcome was assessed.	Not assessable (domain not measured)	High
Tarek 2024 (92)	34 (34, 100%, GMFCS 4–5)	Prospective observational	6 months	None validated; clinician-rated swallowing	Swallowing ability improved significantly in the peptide-formula arm (*p* = 0.002). No cognitive or developmental outcome was assessed.	Not assessable (domain not measured)	Serious
Kaur 2019 (93)	95 (≈73, ≈77%)	Retrospective single-centre	Not reported	None; tube removal as a proxy for recovery	33 tubes were removed as the child's condition improved and oral intake resumed, with a further 6 removed at parental request. Recovery of oral feeding is reported rather than any developmental outcome.	Not assessable (domain not measured)	*Not appraised*
Benoit 2001 (64)	325 (49 CP, 15%)	Retrospective chart review	≥12 months in 62%	None	Developmental delay was recorded in 100/107 (93%) of those with data in the ≥12-month subgroup, but as a baseline characteristic of a mixed population; no measure was applied and no outcome assessed.	Not assessable (baseline characteristic only)	Serious

Cognition, gross and fine motor function, communication, adaptive behaviour, school participation and seizure control, by any instrument. Only Wu 2026 applied a standardised instrument. WeeFIM, Functional Independence Measure for Children; IDDSI, International Dysphagia Diet Standardisation Initiative; NI, neurological impairment. Swallowing function and recovery of oral feeding are outcomes of the feeding route rather than of neurodevelopment and are classified as not assessable for this domain.

#### 3.2.6 Gastrointestinal outcomes

Gastro-oesophageal reflux after gastrostomy is the one outcome domain in this review where the direction of effect depends less on study design than on the child's reflux status before the operation. Only 12 of the 56 studies (21%) explicitly stratified patients according to the presence or absence of reflux before gastrostomy, allowing postoperative outcomes to be interpreted in relation to baseline reflux status (26, 34, 56–58, 75, 79–81). A further four studies provided partial subgroup or symptom-based tracking, whereas the remaining 40 studies (71%) did not stratify outcomes by pre-gastrostomy reflux status. Consequently, direct assessment of *de novo* reflux among patients without pre-existing disease and changes in reflux among those with established disease was possible in only a minority of studies.

Fifty-nine studies reported new or worsened reflux, physiological reflux measurement before and after placement, or the rate of subsequent (non-concurrent) antireflux surgery. We classified the direction of effect as favourable in 19 studies, unfavourable in 14, mixed in 15, no difference in three, and not assessable in eight, which either reported baseline reflux prevalence only, did not separate the neurologically impaired subgroup, or did not delineate reflux from a composite gastrointestinal outcome.

The physiological studies are the most informative and they agree with one another. Thomson et al. (53) measured pH and impedance before and after PEG in ten children and found total reflux events rose from 183 to 355 (*p* = 0.047), acid events from 27 to 173 (*p* = 0.028), and the median distal reflux index from 0.25% to 2.95% (*p* = 0.032); no child had an abnormal reflux index before PEG and three of ten did afterwards. Machado et al. (94) found a significant increase in weakly acid reflux episodes after PEG (mean difference +17.4, 95% CI: 1.6–33.5, *p* = 0.04), and Demirbaş et al. (44) found the total bolus exposure index rose in the gastrostomy-alone arm from 4.01 to 5.67 (*p* = 0.041). Toporowska-Kowalska et al. (95) add a qualification rather than a contradiction: the number of children with pathological reflux fell from 6/15 to 2/15, but the refluxate became significantly more acidic (*p* < 0.001). This study was judged to have moderate risk of bias.

Kawahara et al. (96, 97), both at moderate risk of bias, provide the mechanism that reconciles the apparently conflicting clinical literature. In both of their cohorts the effect of gastrostomy on reflux ran in opposite directions depending on baseline status. Among children without pre-operative pathological reflux the median reflux index rose significantly (2.1% to 4.5%, *p* = 0.004) and roughly a third crossed into the abnormal range, while among children who already had pathological reflux the index fell significantly (11.2% to 9.8%, *p* = 0.04) and a third normalised. Park et al. (78) found the same pattern clinically, with aggravated reflux in 6.3% overall but less severe disease in 13 of 15 children who had histological GERD before the operation, although this study was at serious risk of bias. Taken together, the moderate-risk studies suggest that gastrostomy may worsen reflux in children who did not have it and improve it in some children who did, although this pattern should be interpreted cautiously given the observational evidence base. Franken et al. (34), the largest cohort in this domain (*n* = 300) and judged to have moderate risk of bias, illustrates the consequence: overall GERD symptom prevalence was essentially unchanged (57.8% to 54.2%, *p* = 0.824), a null result that conceals *de novo* reflux in 34% of children who had none beforehand and resolution in 28% of those who did.

*De novo* reflux after gastrostomy alone is common. Langer (1988) (58) reported the highest rate in this table, with 22 of 50 surviving children (44%) who had no demonstrable reflux pre-operatively developing symptomatic reflux after gastrostomy alone, at a mean of nine months, however, this study was at serious risk of bias. Puntis et al. (77) found symptomatic reflux in 15 of 27 children (55.5%) after PEG, also in a study at serious risk of bias, Saadah et al. (98) in 16 of 25 (64%), and Heine et al. (1995) (83), at moderate risk of bias, found the clinical severity of reflux increased in 8 of 30 (*p* < 0.005), exacerbating disease in roughly a quarter of children with previously no or mild reflux. Aumar et al. (63), the largest prospective dataset in this domain and one of the lower-risk studies (low/moderate risk of bias), found *de novo* GERD in 12% and worsening in 25% of those with pre-existing disease. Against these, several cohorts report much lower *de novo* rates, most of them retrospective and symptom-based rather than physiological: Khattak et al. (79) 4% of neurologically impaired children, Viswanath et al. (61) 3.8%, and Sakamoto et al. (69) 2.4%.

Subsequent antireflux surgery is the outcome with the most direct bearing on practice, and its rate is consistently modest across studies, although confidence in individual estimates varied with risk of bias. Across the studies reporting it, the proportion of children who went on to require a fundoplication after gastrostomy alone clustered between 0% and 17%, most commonly between 4% and 8%: Franken (34) 0.7%, Wadman (99) 4.3%, Åvitsland (81) 4%, Zambaiti (100) 6.3%, Guillén Redondo (82) 7.2%, Ulman (101) 7.5%, Puntis (77) 7.4%, Dreznik (80) 10.1%, and Williams (102) 3.3% at five years alongside a further 10.2% converted to a gastrojejunal tube. These estimates were drawn predominantly from observational studies with varying risk of bias, although the broadly similar rates across studies provide some consistency to the finding. Of note, Nelson et al. (57), a study with low/moderate risk of bias, reported subsequent antireflux surgery in 5.3% at five years, mirroring the findings of the higher-risk studies. The outliers are informative rather than anomalous. Viswanath et al. (61) reported 17.1% in the era when their unit performed gastrostomy alone and reserved fundoplication for medical failure, which is the expected consequence of that policy rather than evidence of harm, although the observational design limits causal interpretation. Harrington et al. (103) reported 23% within two years and identified the reason: a seizure disorder raised the risk of requiring subsequent antireflux surgery more than fourfold (*p* = 0.03), with a negative predictive value of 87%, however, this association arose from a study at serious risk of bias and should therefore be interpreted cautiously. Dreznik et al. (80) found the converse in the same population, with 88% of neurologically impaired children with refractory seizures having recurrent reflux symptoms or aspiration pneumonia even after fundoplication. As for Catto-Smith (35), they reported that 14% of their cohort developed clinically significant reflux with aspiration post-gastrostomy, all of whom required subsequent fundoplication, placing their reoperation rate at the higher end of the reported 0%–17% cluster (35).

Two controlled comparisons address whether a concurrent antireflux procedure prevents this. Redondo et al. (82) found that among children with pre-existing reflux, symptoms worsened in 36% after gastrostomy alone against 15% after gastrostomy with a concurrent antireflux technique. Barnhart et al. (104), a propensity-matched cohort of 4,163 infants, found no difference between matched pairs in reflux-related hospitalisation (difference −0.05 per year, 95% CI: −0.20 to 0.15), so the available controlled evidence does not demonstrate that adding a fundoplication translates into fewer reflux-related admissions. Although these comparative data are methodologically stronger than the uncontrolled series, residual confounding remains inherent to the non-randomised designs. Set against the major-complication findings reported in section 3.3.4, where concurrent antireflux surgery accounted for most of the excess serious morbidity, these findings illustrate the potential trade-off between reflux control and procedural morbidity, although the evidence is insufficient to establish the optimal approach.

Overall, despite the predominantly observational evidence and variable risk of bias, reflux after gastrostomy is common, is frequently new in children who did not have it before, and improves in a substantial minority of those who did. Across studies, most cases did not progress to antireflux surgery, with subsequent fundoplication generally reported in fewer than 10% of children, and studies with low/moderate risk of bias observed broadly similar estimates. Seizure disorder was identified as a potential predictor of subsequent antireflux surgery in one study, but this finding requires cautious interpretation given its serious risk of bias.

Study-level data for this domain are presented in Table 8.

**Table 8:** Gastro-oesophageal reflux and subsequent antireflux surgery after gastrostomy.

Author/Year	N (NI)	Design	Follow-up	Stratified by Reflux	Finding	Direction	Risk of Bias
Zhang 2025 (23)	84 (84, 100%)	Randomised controlled trial (PEG vs. indwelling NG)	28 days	Yes	Reflux oesophagitis during the treatment period in 2/42 (4.8%) PEG versus 9/42 (21.4%) NG controls (*p* = 0.049). No pH, impedance or endoscopic assessment and no baseline reflux status, so prevalent and new disease cannot be distinguished.	Favourable (vs. NG comparator)	High
Catto-Smith 2006 (35)	98 (100%)	Retrospective cohort	6–14 years		14% new GERD w/aspiration, 100% of those required fundoplication	Unfavourable	*Serious*
Grayeb 2025 (39)	372 (327, 88%)	Retrospective cohort	Median 64 months		45/372 (12%) underwent fundoplication during follow-up, of whom 9/45 (20%) required a second antireflux procedure. PPI use (HR: 1.95, 95% CI: 1.66–3.76, *p* < 0.001) and inhaled steroids/bronchodilators (HR: 1.25, 95% CI: 1.1–3.05, *p* = 0.041) predicted fundoplication.	Mixed (moderate reoperation burden)	Low
Tarek 2024 (80)	34 (34, 100%)	Prospective observational (peptide vs. casein formula)	6 months		Reflux decreased significantly in the peptide arm (*p* = 0.034); no significant change in the casein arm.	Favourable (peptide arm only)	Serious
Jadi 2023 (65)	63 (NI subgroup)	Retrospective chart review	12 months		76.1% had GERD at baseline. Post-operatively 14/63 (22%) had “functional bowel problems” combining GERD with diarrhoea and constipation; the reflux-specific numerator is not separable.	Not assessable (outcome not delineated)	Serious
Williams 2023 (92)	6,118 (4,092 NI)	Retrospective cohort	Median 3.78 years		Among NI children who had an isolated gastrostomy, 3.3% required a delayed fundoplication by 5 years and 10.2% required conversion to a gastrojejunal tube; 12.6% required either.	Mixed (low fundoplication rate, substantial jejunal conversion)	Low/Moderate
Dreznik 2022 (80)	345 (158, 45.7%)	Retrospective cohort	Median 5 years		Late fundoplication in 16 NI patients (10.1% of the NI group). Among NI patients with refractory seizures who underwent fundoplication, 88% had recurrent GERD symptoms or aspiration pneumonia.	Unfavourable (high recurrence in the refractory-seizure subgroup)	Serious
Masui 2022 (119)	35 (35, 100%)	Retrospective cohort	∼1 year		No new or worsened gastro-oesophageal reflux recorded.	Favourable	Moderate
Guillén Redondo 2021 (82)	207 (153, 73.9%)	Retrospective cohort	Mean 40.6 months		Among children with pre-existing GER, reflux worsened in 20/55 (36%) after gastrostomy alone versus 6/41 (15%) after gastrostomy with a concurrent antireflux technique. Among children without pre-existing GER, new reflux developed in 18/111 (16%). 12/166 (7.2%) without a concurrent fundoplication later required one.	Unfavourable (gastrostomy alone; risk halved by a concurrent antireflux procedure)	Serious
Ulman 2021 (101)	109 (109, 100%)	Retrospective cohort (routine vs. selective GER screening)	Median >2 years		New-onset GER symptoms in 8/109 overall (11.7% routine-screening group, 8.7% selective-screening group). 6/80 (7.5%) of those who had gastrostomy alone required subsequent fundoplication.	Mixed (low rate, but consistently present)	Moderate
Sakamoto 2021 (63)	89 (82 analysed, 100%)	Retrospective cohort	Median 1,770 days (long-term arm)		2 patients (2.4%) had long-term vomiting and were subsequently diagnosed with GERD.	Favourable (low rate)	Moderate
Zambaiti 2021 (90)	145 (145, 100%)	Retrospective cohort	Median 4 years		8 of the 127 children who had gastrostomy alone (6.3%) required subsequent fundoplication for therapy-resistant GER symptoms.	Favourable (low rate)	Serious
Wadman 2021 (99)	146 (23 gastrostomy)	Retrospective chart review	Median 4 years		1 of the 23 gastrostomy patients (4.3%) had worsening reflux, and that patient required a subsequent Nissen fundoplication.	Favourable (low rate)	Moderate/High
Demirbaş 2021 (44)	20 (20, 100%)	Prospective (gastrostomy vs. gastrostomy + Nissen)	Mean 16.5 months		Total bolus exposure index rose in the gastrostomy-alone arm from 4.01 to 5.67 (*p* = 0.041); the pH index change was not significant (*p* = 0.061).	Unfavourable (physiological, gastrostomy alone)	Serious
Noviello 2021 (75)	148 (111, 75%)	Retrospective review	Variable (6-monthly review)		GERD present in 49/148 (33%) pre-operatively; 2 NI patients had post-operative recurrence, neither treated surgically. 5 gastrojejunal tubes were placed pending fundoplication.	Favourable (low recurrence)	Moderate
Franken 2020 (37)	50 (36, 72%)	Prospective longitudinal cohort with MII-pH	Median 3.9 months		Pathological gastro-oesophageal reflux was unchanged at 72% before and 72% after gastrostomy (*p* = 1.00), with 4 new cases and 4 resolutions; acid exposure time 6.2% to 6.1% (NS). No child required antireflux surgery.	No difference	Moderate/High
Suh 2020 (7)	18 (18, 100%)	Retrospective cohort	Median 50.4 months		Reflux outcome not separately reported.	Not assessable	Moderate
Tavares de Sousa 2020 (31)	28 (28, 100% CP)	Cross-sectional	Not applicable		GERD reported in 11/28 (39%); cross-sectional design with no baseline.	Not assessable (no before/after comparison)	Moderate
Lertudomphonwanit 2019 (59)	201 (168, 83.6%)	Retrospective cohort	1 year		GERD in 17/201 (8.5%) at one-year follow-up, described by the authors as mostly neurologically impaired patients; the NI-specific numerator is not given.	Favourable (low rate; not NI-isolated)	Serious
AlNamshan 2019 (55)	178 (178, 100%)	Retrospective cohort	Median 36–44 months		Wrap failure with recurrence of GERD symptoms in 3 patients (1.7%), all requiring redo fundoplication.	Favourable (low recurrence)	Serious
Nelson 2019 (44)	948 (948, 100%)	Population-based exposure-crossover study	Up to 5 years		Reflux-related hospitalisations rose from 526 in the two years before tube placement to 694 in the two years after, against unchanged overall acute health-care use. 50 children (5.3%) underwent subsequent antireflux surgery within 5 years.	Mixed (more reflux-related admissions; low subsequent reoperation rate)	Low/Moderate
Åvitsland 2013 (81)	58 (39, 67%)	Prospective cohort	18 months		2 children (4%) underwent antireflux surgery between 6 and 18 months.	Favourable (low rate)	Moderate
Aumar 2018 (54)	326 analysed (208, 56%)	Prospective and retrospective observational	Median 3.5 years		*De novo* GERD appeared in 38/326 (12%), and GERD worsened in 25% of those with pre-existing disease. 53 patients (16%) required antireflux surgery after PEG, 22 of them within the first year.	Unfavourable	Low/Moderate
Ercoli 2017 (128)	224 (81.4% NI)	Cohort	Not reported		27 patients (12%) underwent antireflux surgery.	Mixed (moderate reoperation rate)	Serious
Machado 2016 (94)	12 (12, 100%)	Longitudinal, MII-pH	5 months		Weakly acid reflux episodes increased significantly after PEG (mean difference +17.4, 95% CI: 1.6–33.5, *p* = 0.04).	Unfavourable (physiological)	Serious
Breton 2016 (129)	259 (164, 63%)	Retrospective cohort	Median 3.2 years		New-onset reflux in 25 (10%) and worsening in 28; 81% of affected children were neurologically impaired.	Unfavourable	*Not appraised*
Koca 2015 (68)	34 (22 neurological, 64.7%)	Retrospective cohort	Mean 19.4 months		Antireflux medication use rose from 16/34 (47.1%) to 18/34 (52.9%) after PEG (*p* = 0.62, NS).	No difference	Serious
Franken 2015 (26)	300 (225, 75%)	Retrospective cohort	Median 2.63 years		GERD symptom prevalence was essentially unchanged overall (57.8% pre-operatively to 54.2% post-operatively, *p* = 0.824), but this masked substantial two-way movement: *de novo* reflux symptoms developed in 34% of children without pre-operative GERD, while GERD resolved in 28% of those who had it. 2 patients (0.7%) required secondary antireflux surgery.	Mixed (no net change; high *de novo* incidence)	Moderate
Kehar 2014 (48)	46 (≈34, ≈74%)	Prospective cohort	Up to 12 months		3 patients had worsened reflux, managed by conversion to a jejunal extension rather than surgery; aspiration pneumonia secondary to reflux in 2.	Unfavourable (small numbers)	Serious
Lertudomphonwanit 2014 (131)	133 (133, 100%)	Retrospective cohort	Not reported		GERD present in 45.8% at baseline; post-gastrostomy change not reported. 6.8% required fundoplication for GERD.	Not assessable (baseline prevalence only)	*Not appraised*
Kawahara 2014 (96)	26 (26, 100%)	Retrospective cohort, stratified by pre-operative reflux index	Median 46 months		Physiological reflux worsened in 13/26 (50%), confined entirely to the subgroup without pre-operative pathological reflux, in whom both the reflux index and the total number of reflux episodes rose significantly. No child (0%) required surgery for clinical deterioration. The subgroup with pre-existing pathological reflux showed a significant reduction in reflux episodes and improved gastric emptying.	Mixed (physiological deterioration confined to the reflux-negative subgroup; no clinical consequence)	Moderate
Toporowska-Kowalska 2013 (122)	348 (285/343, 81.9%)	Retrospective multicentre questionnaire	12 months		81/87 (93.1%) of all patients who underwent fundoplication in the cohort were neurologically impaired; the whole-cohort fundoplication rate was 87/336 (25.9%). Reflux status before and after gastrostomy is not separable by neurological status.	Not assessable (not NI-separable)	Moderate
O'Loughlin 2013 (43)	122 (122, 100%)	Retrospective cohort + caregiver questionnaire	Median 77 months		Slipped fundoplication wrap with hiatus hernia and symptom recurrence in 3 children; redo fundoplication in 3/122 (2.5%).	Favourable (low recurrence)	Moderate/High
Barnhart 2013 (93)	4,163 (4,163, 100%)	Retrospective propensity-matched cohort (fundoplication + GT vs. GT alone)	1 year post-NICU discharge		No difference between matched pairs in reflux-related hospitalisation rates (difference −0.05 per year, 95% CI: −0.20 to 0.15). Concomitant fundoplication conferred no reflux-related advantage.	No difference	Moderate
Park 2011 (72)	32 (32, 100%)	Retrospective cohort	Mean 12.2 months		Aggravated reflux in 2 patients (6.3%), one of whom (3.1%) required a Nissen fundoplication 10 months after PEG. Conversely, of 15 patients with pre-operative histological evidence of GERD, 13 (87%) had less severe disease afterwards.	Mixed (favourable where reflux pre-existed)	Serious
Thomson 2011 (53)	10 (10, 100%)	Prospective before-after pH/impedance study	Median 55 days		Total reflux events rose from 183 to 355 (*p* = 0.047) and acid reflux events from 27 to 173 (*p* = 0.028); the median distal reflux index rose from 0.25% to 2.95% (*p* = 0.032). No child had an abnormal reflux index before PEG; 3/10 (30%) did afterwards.	Unfavourable (physiological)	Moderate/High
Toporowska-Kowalska 2011 (95)	15 (15, 100%)	Prospective cohort with MII-pH	6–8 months		The number of children with pathological reflux fell from 6/15 (40%) to 2/15 (13.3%), but the composition of the refluxate shifted: highly acidic events rose significantly and weakly acidic events fell (*p* < 0.001).	Mixed (fewer children affected; more acidic refluxate)	Moderate
Kawahara 2010 (97)	54 (54, 100%)	Retrospective cohort, stratified by pre-operative reflux index	Median 4 years		In children without pre-operative pathological reflux the median reflux index rose from 2.1% to 4.5% (*p* = 0.004), and 8/22 (36%) tested crossed into the abnormal range. In children with pre-operative pathological reflux the index fell from 11.2% to 9.8% (*p* = 0.04) and 6/18 (33%) normalised. 1 patient (1.9%) underwent fundoplication 5 months after gastrostomy.	Mixed (direction depends on baseline reflux status)	Moderate
Viswanath 2010 (53)	137 (137, 100%)	Retrospective cohort across three management protocols	Median 3.1 years		New symptomatic reflux in 1/10 (10%) of the selectively screened group and 2/53 (3.8%) of the gastrostomy-alone group with no pre-operative pH-proven reflux. Subsequent fundoplication was required in 14/82 (17.1%) of the gastrostomy-alone era for reflux failing maximal medical management.	Mixed (low new-onset rate; substantial subsequent fundoplication)	Serious
Sathesh-Kumar 2009 (70)	76 (≈76% NI)	Cohort study	Median 52 months		Worsening reflux in 4 patients (5.3%), of whom 3 (3.9%) underwent subsequent fundoplication.	Favourable (low rate)	Moderate/High
Saadah 2009 (98)	25 (25, 100%)	Retrospective cohort	6 months		Clinical reflux, in the form of persistent vomiting, in 16/25 (64%); 12 were controlled with proton pump inhibitor and prokinetic therapy and 4 (16%) required fundoplication, performed 5 to 60 months after PEG.	Unfavourable (high symptomatic rate)	Serious
Motil 2009 (27)	95 (95, 100%)	Retrospective cohort	Mean 4.5 years		Children without a fundoplication were more likely to be receiving proton pump inhibitor (60% vs. 26%, *p* < 0.01) and prokinetic (37% vs. 12%, *p* < 0.01) medication than those with one; no before-and-after reflux comparison.	Not assessable (no baseline comparison)	Serious
Ramelli 2007 (56)	31 (31, 100%)	Longitudinal cohort	Mean 5 years		3 patients developed reflux after gastrostomy, of whom 1 required fundoplication 15 months later.	Favourable (low rate)	Serious
Craig 2006 (124)	76 (76, 100%)	Prospective cohort	6 months		3 children (4%) required revision of their original antireflux procedure. New or worsened reflux is not reported separately from the antireflux-procedure denominator.	Not assessable	Moderate
Sullivan 2006 (11)	57 (57, 100%)	Prospective longitudinal cohort	6 and 12 months		Minor symptoms suggestive of reflux developed or persisted in some children, all managed successfully with proton pump inhibitor therapy. No fundoplication was required. ^a^Same Oxford cohort as Sullivan 2005.	Favourable	Moderate/High
Sullivan 2005 (33)	57 (57, 100%)	Prospective longitudinal cohort	12 months		No fundoplication was required for post-operative GERD. ^a^Same Oxford cohort as Sullivan 2006.	Favourable	Moderate
Harrington 2004 (88)	102 (102, 100%)	Retrospective cohort (gastrostomy ± concurrent antireflux procedure)	2 years post-discharge		Among children who had gastrostomy alone with complete follow-up, 7/30 (23%) developed GERD requiring antireflux surgery within 2 years. A seizure disorder raised that risk more than fourfold (present in 4/7 of those requiring surgery versus 3/23 of those not, *p* = 0.03; positive predictive value 57%, negative predictive value 87%).	Unfavourable (high subsequent surgery rate; seizure disorder as predictor)	Moderate
Wales 2002 (56)	111 (111, 100%)	Retrospective cohort (fundoplication + gastrostomy vs. gastrojejunal tube)	Mean 3.1 years (GJ)/5.7 years (FG)		Continued antireflux medication in 70.8% of gastrojejunal versus 23.8% of fundoplication patients (*p* < 0.05), as expected since a gastrojejunal tube bypasses rather than treats reflux. Wrap failure occurred in 14.3% of fundoplication patients, of whom 11.1% had a repeat fundoplication; 8.3% of gastrojejunal patients went on to require fundoplication.	Mixed (device-dependent)	Serious
Puntis 2000 (77)	29 (27 PEG, 100%)	Retrospective cohort	Median 2.6 years		Symptomatic reflux developed after PEG in 15/27 (55.5%), most managed medically; 2/27 (7.4%) required a Nissen fundoplication.	Unfavourable (high symptomatic rate)	Serious
Arnbjörnsson 1999 (107)	98 (≈75%)	Retrospective cohort	Mean 39 months		3 patients underwent subsequent fundoplication.	Favourable (low rate)	Serious
Smith 1999 (9)	40 interviewed (100% CP)	Population-based retrospective cohort + caregiver interview	Mean 8.5 years		27/40 (67.5%) had pre-existing GERD and all underwent fundoplication; new or worsened reflux after gastrostomy is not reported.	Not assessable (baseline prevalence only)	Moderate/High
Khattak 1998 (73)	120 (52, 43%)	Retrospective case series	Mean 26.6 months		NI-separable: reflux developed in 5 previously asymptomatic NI children (4% of the 52 NI patients). Among NI children with pre-PEG reflux (*n* = 18), 38% required an antireflux procedure; 9/52 NI children (19%) had a Nissen fundoplication overall.	Mixed (low *de novo* rate; high reoperation where reflux pre-existed)	Moderate (JBI)
Borowitz 1997 (66)	19 (19, 100%)	Prospective cohort	Mean 20.7 months		New or worsening vomiting in 1 of 9 children with pre-operative evidence of GERD and 1 of 10 without. No fundoplication was required.	Favourable	Serious
Heine 1995 (105)	30 (30, 100%)	Prospective before/after with caregiver questionnaire, pH study and endoscopy	3–9 months		Clinical severity of reflux increased after PEG in 8/30 (*p* < 0.005), with recurrent vomiting and pulmonary aspiration; PEG exacerbated reflux in roughly a quarter of children who previously had no or mild disease. 5/30 (17%) underwent subsequent Nissen fundoplication.	Unfavourable	Moderate
Cameron 1995 (74)	63 (63, 100%)	Retrospective cohort (PEG vs. Stamm gastrostomy)	Mean 23 months		Subsequent reflux occurred in 60% of both groups, but severe reflux requiring fundoplication was far less frequent after PEG than after Stamm gastrostomy (10% vs. 39%, *p* < 0.025).	Mixed (high overall incidence; favours PEG for severity)	Serious
Sanders 1990 (40)	51 (51, 100% CP)	Retrospective before-after cohort	Mean 2.4 years		10% had continuing reflux symptoms or complications.	Favourable (low rate)	Moderate
Stringel 1989 (91)	90 (90, 100%)	Retrospective case series	3 months–3 years		No clinical evidence of reflux recurrence at three-year follow-up; a Nissen fundoplication was performed as the primary antireflux procedure in this series.	Favourable (concurrent fundoplication)	Serious
Langer 1988 (55)	107 (107, 100%)	Retrospective cohort (gastrostomy ± antireflux procedure)	Mean ∼20 months		Of 54 children with no demonstrable reflux before surgery, 22 of the 50 survivors (44%) developed symptomatic reflux after gastrostomy alone, at a mean of 9 months (range 0–24). Of 11 children with pre-operative reflux who had gastrostomy alone, 7 developed symptomatic reflux. Approximately 21 gastrostomy-alone patients went on to have an antireflux procedure.	Unfavourable (highest *de novo* rate in this table)	Serious
Shapiro 1986 (94)	19 (19, 100%)	Retrospective before/after	Mean 23.1 months		Persistent vomiting in 5 children at follow-up; 2/19 required a fundoplication in addition to the 5 performed concurrently.	Mixed	Moderate/High
Wesley 1981 (90)	33 (22, 66.7%)	Retrospective case series	Mean 11 months		Recurrent reflux with an intact wrap in 2/22 (9%) at 6 and 9 months, demonstrated on barium study through the gastrostomy tube; both were being assessed for reoperation at publication. A further 2/22 (9%) required reoperation for early technical failure of the wrap.	Unfavourable	Moderate/High

New or worsened reflux, physiological reflux measurement before and after placement, and rate of subsequent (non-concurrent) antireflux surgery. GER, gastro-oesophageal reflux; GERD, gastro-oesophageal reflux disease; MII-pH, multichannel intraluminal impedance with pH monitoring; PPI, proton pump inhibitor; GJ, gastrojejunal; NS, not significant. Concurrent antireflux procedures performed at the same operation are not counted as subsequent surgery.

a Companion publications reporting the same cohort.

#### 3.2.7 Mortality

The evidence was mostly observational and methodologically limited, with most studies assessed at moderate to serious risk of bias and only a small number contributing lower-risk evidence. Accordingly, mortality rates from uncontrolled cohorts were interpreted primarily as descriptive estimates, while greater weight was given to comparator-controlled and population-linked analyses.

Mortality in this population is high, is dominated by the underlying neurological disease, and is only rarely attributable to the gastrostomy itself. Sixty-seven studies reported mortality. We classified the direction of effect as favourable in 40 studies, mixed in 14, unfavourable in six, no difference in one, and not assessable in six. The favourable classification here denotes the absence of procedure-attributable death rather than a survival benefit, since only three studies in the review compared survival against a non-gastrostomy comparator, and those three do not agree.

All-cause mortality during follow-up ranged from 0% in the short-follow-up prospective cohorts to 43% in Lefton-Greif et al. over a mean of 4.11 years, and clustered between 8% and 30% in cohorts followed for two years or longer: Åvitsland (81) 8.6%, Vergara (26) 10.1%, Stringel (91) 10%, Kawahara (97) 13.0%, Ramelli (56) 15.6%, O'Loughlin (43) 16%, Tawfik (89) 17%, Franken (47) 17.3%, Puntis (77) 17.2%, Guillén Redondo (82) 19.3%, Sathesh-Kumar (70) 22.4%, Heine (105) 23%, Grayeb (39) 26%, Smith (9) 26.2%, Koca (74) 26.4%, Dreznik (80) 26.9%, Aumar (63) 29%, Zambaiti (100) 33% and AlNamshan (55) 35%. These figures describe the fragility of the population selected for the procedure and should not be read as an outcome of it. Among the more informative longitudinal datasets, Nelson et al. (57), assessed at low/moderate risk of bias, found two-year survival of 87.4% (95% CI: 85.2–89.4) and five-year survival of 75.8% (95% CI: 72.8–78.4) in 948 children, and Smith et al. (9), although at higher risk of bias, reported the longest survival curve in this review, falling from 91% at one year to 55% at ten. Catto-Smith (35) reported progressive long-term mortality of 11% at 1 year, 21% at 2 years, 27% at 3 years, and 39% at 13 years, with only one early death (cardiac failure) at 7 days.

Procedure-attributable death is rare and, where it occurs, is concentrated in the same places as major complications. Wesley et al. (90) recorded one death directly attributable to the operation, on the eleventh post-operative day following sepsis and wound dehiscence. Viswanath et al. (61) recorded one, on the fourth post-operative day after re-exploration for a gastric perforation sustained during laparoscopic fundoplication, with no deaths in the gastrostomy-alone group. Aumar et al. (63) attributed two of 94 deaths to early procedural complications. Wales et al. (84) recorded two device-attributable deaths in the fundoplication arm, from small-bowel obstruction after fundoplication and from peritonitis following a gastrostomy tube change, and Langer (83) one reflux-attributable death after gastrostomy alone. Against these, a large number of cohorts examined the question explicitly and found nothing: Grayeb (39) (none of 94 deaths), Dreznik (80) (none of 93), Guillén Redondo (82) (none of 40), Franken (34) and Franken (14) (none of 26 in either), Sathesh-Kumar (70), Ramelli (56), Koca (74), Kaur (93), Smith (9), Stringel (91), Park (78) and Åvitsland (81) all state that no death was related to the procedure.

Thirty-day mortality, where reported separately, was 0% to 3% in most cohorts: AlNamshan et al. (55) 3% (5/178), Nelson et al. (57) 1.8% (17/948), and zero in Sathesh-Kumar 2009 (70), Chao (46), Khattak (79), Cameron (62) (PEG arm) and O'Loughlin (43). The two exceptions are the two smallest and most fragile cohorts in the review, and both are informative about patient selection rather than technique: Vajsar et al. (58) recorded 2 deaths among 12 infants with spinal muscular atrophy type 1 within 30 days (16.7%), and Lefton-Greif et al. (48) 3 among 28 (11%), from pneumonia, pneumothorax and respiratory failure.

Three studies compared mortality against children who did not receive a gastrostomy, and their findings conflict significantly. Lin et al. (12), a 1:2 propensity-matched cohort, found that new gastrostomy was associated with a reduced risk of death (HR: 0.47). Downs et al. (60) found survival rates similar to children without a gastrostomy. Crucially, Wong et al. (86), a propensity-score-matched cohort of 212 children with severe intellectual disability (assessed at low/moderate risk of bias), found a significantly increased risk of death in the gastrostomy group (HR: 2.87, 95% CI: 1.13–7.26) over a median of 6.25 years compared to matched controls fed orally. Given that Wong et al. and Lin et al. are the highest-quality comparative studies in this review and point in opposite directions, the evidence does not definitively support a survival benefit and suggests long-term mortality risk may be elevated in some gastrostomy-fed populations (12).

Two further observations bear on timing, although both derive from small studies at serious risk of bias. Sanders et al. (40) found mortality rising steeply with delay to intervention, from 7% in the earliest-treated group to 40% in the latest, and Heine et al. (105) found that a falling weight-for-age *z*-score after PEG identified children at risk of death, with four of the eight children whose *z*-score fell subsequently dying. Both were uncontrolled and susceptible to confounding by the severity that prompted delayed intervention; these findings should therefore be considered hypothesis-generating rather than evidence that earlier gastrostomy reduces mortality.

Overall, children receiving gastrostomy for neurological impairment had substantial all-cause mortality, but deaths were predominantly attributed to underlying disease rather than the procedure itself. Procedure-attributable mortality was rare, and 30-day mortality was generally 0% to 3% outside the most fragile diagnostic groups. Although confidence in precise mortality estimates is limited by the predominance of observational studies at moderate to serious risk of bias, the lower-risk evidence similarly suggests that gastrostomy itself is rarely a cause of death. Evidence that gastrostomy improves survival, however, remains limited, with only one moderate-risk propensity-matched study demonstrating a reduction in short-term mortality.

Study-level data for this domain are presented in Table 9.

**Table 9:** Mortality after gastrostomy.

Author/Year	N (NI)	Design	Follow-up	Finding	Direction	Risk of bias
Lin 2021 (41)	2,490 (2,490, 100%)	Retrospective 1:2 propensity-matched cohort	180 days (sensitivity to 365/548 days)	219 children (9%) died in the eligible cohort. New gastrostomy was associated with a reduced risk of death (HR: 0.47), even though the composite of death or severe pneumonia was increased (HR: 1.21). This is the only comparator-controlled mortality estimate in the review.	Favourable (vs. no-gastrostomy comparator)	Moderate
Catto-Smith 2006 (35)	98 (100%)	Retrospective cohort	6–14 years	11%/21%/27%/39% at 1/2/3/13 yrs; 1 death (cardiac, day 7) not clearly procedure attributable)	Unfavourable	Serious
Downs 2019 (severe ID cohort) (135)	1,011 (1,011, 100%)	Retrospective population cohort	Up to 23 years	50% survival to 23 years among children with a gastrostomy. Compared with no gastrostomy, mortality was similar for both females and males.	No difference (vs. no-gastrostomy comparator)	Not appraised
Nelson 2019 (44)	948 (948, 100%)	Population-based exposure-crossover study	Up to 5 years	2-year survival 87.4% (95% CI: 85.2–89.4), with 121 deaths within 2 years including 17 (1.8%) within 28 days of tube placement; 5-year survival 75.8% (95% CI: 72.8–78.4), 222 deaths.	Mixed (high background mortality; 1.8% early mortality)	Low/Moderate
Grayeb 2025 (39)	372 (327, 88%)	Retrospective cohort	Median 64 months	94/372 (26%) died during childhood follow-up, at a median of 26 months (IQR: 12–67) after insertion. None of the deaths were attributed to the insertion procedure.	Favourable (no procedure-attributable deaths)	Low
Pinheiro 2025 (32)	18 (18, 100% congenital Zika syndrome)	Prospective cohort, before-after	∼1 year	1 child died within the first year and was excluded from the outcome analysis; cause and relationship to the procedure not stated.	Not assessable	Serious
Dreznik 2022 (80)	345 (158, 45.7%)	Retrospective cohort	Median 5 years	93 deaths (26.9%) overall, of whom 47 were NI patients (29.7% of the NI group). None were related to the surgical procedure.	Favourable (no procedure-attributable deaths)	Serious)
Guillén Redondo 2021 (82)	207 (153, 73.9%)	Retrospective cohort	Mean 40.6 months	40/207 (19.3%) died, all attributed to the underlying condition rather than the gastrostomy.	Favourable (no procedure-attributable deaths)	Serious
Zambaiti 2021 (90)	145 (145, 100%)	Retrospective cohort	Median 4 years	48 patients (33%) died at a median of 2.67 years after surgery, from disease progression (26), respiratory infection (14) and other causes. No significant difference in mortality between the gastrostomy-alone and fundoplication groups.	Mixed (high background mortality; no excess with fundoplication)	Serious
Ulman 2021 (101)	109 (109, 100%)	Retrospective cohort	Median >2 years	7 patients died during follow-up for reasons unrelated to the surgery; these 7 were excluded from the analysed cohort of 109.	Favourable (no procedure-attributable deaths)	Moderate
Sakamoto 2021 (63)	89 (82 analysed, 100%)	Retrospective cohort	Median 1,770 days (long-term arm)	2/89 (2.2%) died within 2 years, from underlying disease and unrelated to the surgery or to aspiration.	Favourable	Moderate
Işık & Akbulut 2021 (108)	91 (70, 76.9%)	Retrospective single-centre	6 months–5 years	4 deaths (4.4%), none gastrostomy-related.	Favourable	Serious
Noviello 2021 (75)	148 (111, 75%)	Retrospective review	Variable	1 death, from truncus arteriosus with Simpson-Golabi-Behmel syndrome, in a non-NI patient.	Favourable	Moderate
Franken 2020a (14)	150 (≈74% NI)	Cross-sectional	Median 4.0 years	26/150 (17.3%) died during follow-up, from deterioration of neurological disease (23), cystic fibrosis (2) and advanced cardiac disease (1). None related to the gastrostomy.	Favourable (no procedure-attributable deaths)	Moderate/High
Franken 2020b (37)	50 (36, 72%)	Prospective longitudinal cohort	Median 3.9 months	No deaths during follow-up.	Favourable	Serious
Suh 2020 (7)	18 (18, 100%)	Retrospective cohort	Median 50.4 months	1 patient died of the underlying disease; timeframe not specified.	Favourable	Moderate
Jacoby 2020 (42)	673 (673, 100%)	Population-based retrospective cohort	Median 4.8–7.5 years	113 deaths (34.8%) in the older cohort; 21 (19.6%) in the Western Australian and 37 (10.6%) in the New South Wales recent cohorts. Causes not specified. ^a^Same linked cohort as Downs 2019.	Not assessable (causes not specified)	Low
AlNamshan 2019 (55)	178 (178, 100%)	Retrospective cohort	Median 36–44 months	35% mortality across the 15-year study period; causes were aspiration pneumonia (43%), septic shock (26%), undetermined (22%), renal failure (6%) and cardiac (3%). 5 deaths (3%) occurred within the first 30 post-operative days. Male sex (OR: 2.2, *p* = 0.027) and do-not-resuscitate status (OR 5.2, *p* < 0.001) predicted death.	Unfavourable (highest cohort mortality; 3% 30-day mortality)	Serious
Kaur 2019 (93)	95 (≈73, ≈77%)	Retrospective single-centre	Not reported	8 patients died with the PEG *in situ*, from their primary illness; no PEG-related mortality.	Favourable	Not appraised
Wong 2020 (87)	1,011	Retrospective propensity score matched cohort study	Median 6 years 2 months (IQR: 3 years 7 months to 8 years 9 months)	HR: 2.87 (95% CI: 1.12–7.26), significantly increased death risk vs. matched controls	Unfavourable	Not appraised
Aumar 2018 (54)	326 analysed (208, 56%)	Prospective and retrospective observational	Median 3.5 years	94 children (29%) had died at last follow-up; 2 deaths were attributed to early procedural complications and the remainder to progression of the underlying disease.	Mixed (2 procedure-attributable deaths)	Low/Moderate
Stewart 2017 (85)	535 reviewed (29 with buried bumper syndrome)	Retrospective case-note review	Mean 1,025 days to buried bumper syndrome	No post-operative deaths among those undergoing surgical correction of buried bumper syndrome. Overall cohort mortality not reported.	Favourable (no deaths from the corrective procedure)	Moderate
Figueroa 2017 (24)	81 (81, 100%)	Prospective cohort	1 year	4 patients (4.9%) died of respiratory infection during the first year, at ages 5, 5, 8 and 18 years.	Mixed (respiratory deaths; not procedure-attributed)	Moderate
Franken 2015 (26)	300 (225, 75%)	Retrospective cohort	Median 2.63 years	26 patients (8.7%) died, from deterioration of neurological disease (23), cystic fibrosis (2) and advanced cardiac disease (1). None related to the gastrostomy. ^a^Identical figures to Franken 2013 from the same institution and period; counted once.	Favourable (no procedure-attributable deaths)	Moderate
Koca 2015 (68)	34 (22 neurological, 64.7%)	Retrospective cohort	Mean 19.4 months	9 patients (26.4%) died from factors related to their underlying disease; no death was directly related to the procedure.	Favourable	Serious
Kehar 2014 (48)	46 (≈34, ≈74%)	Prospective cohort	Up to 12 months	Mortality is recorded inconsistently across two extraction records of the same Covidence entry: one states that 21.8% of NI patients died during follow-up, the other that there were no deaths. The discrepancy could not be resolved from the extraction alone and the study is therefore not counted in either direction. ^b^Data-quality flag.	Not assessable (internally inconsistent)	Serious
Åvitsland 2013 (81)	58 (39, 67%)	Prospective cohort	18 months	5 children (8.6%) died from progression of the underlying disease; none procedure-related.	Favourable	Moderate
O'Loughlin 2013 (43)	122 (122, 100%)	Retrospective cohort + questionnaire	Median 77 months	20 patients (16%) died, from pneumonia or aspiration (13), central hypoventilation (1), seizures (3) and unknown causes (3). No death was directly attributable to surgery within one month.	Mixed (substantial aspiration mortality; none procedure-attributable)	Moderate/High
Lefton-Greif 2011 (38)	28 (28, 100%)	Retrospective review	Mean 4.11 years	12 patients (43%) died. Early mortality within 30 days was 3 (11%), from pneumonia, pneumothorax and respiratory failure. Late deaths (9, 32%) occurred at a mean age of 19.5 years from cancer or lymphoma (5), congestive heart failure (2) and lung complications or recurrent aspiration (2).	Unfavourable (highest 30-day mortality)	Moderate
Park 2011 (72)	32 (32, 100%)	Retrospective cohort	Mean 12.2 months	1 patient (3.1%) died of the underlying disease; no mortality associated with the PEG procedure.	Favourable	Serious
Vergara 2011 (22)	55 (55, 100% CP)	Prospective comparative cohort	Not reported	Overall mortality 10.1%; the group-specific breakdown is not given in the abstract.	Not assessable	Not appraised
Kawahara 2010 (97)	54 (54, 100%)	Retrospective cohort	Median 4 years	7 patients (13.0%) died during follow-up: 2 suddenly at home 14 and 15 months after the procedure, and 5 at a median of 21 months, from advanced cachexia (1), sudden death at home (2) and progressive deterioration (2).	Mixed (no procedure attribution stated)	Moderate
Vernon-Roberts 2010 (52)	14 (14, 100%)	Prospective longitudinal feasibility study	6 months	3 participants (21.4%) died of respiratory infection within 6 months, attributed to complications of the underlying condition; no complication was directly attributed to the surgery.	Mixed (high short-term mortality; not procedure-attributed)	Not appraised
Viswanath 2010 (53)	137 (137, 100%)	Retrospective cohort across three protocols	Median 3.1 years	1 death (0.73% of the cohort; 1.7% of the 60 fundoplication patients) on the fourth post-operative day, from pulmonary complications after re-exploration for a gastric perforation sustained during laparoscopic fundoplication. No mortality in the gastrostomy-alone group.	Unfavourable (procedure-related death, fundoplication arm)	Serious
Sathesh-Kumar 2009 (70)	76 (≈76% NI)	Cohort study	Median 52 months	No procedure-related deaths within 30 days. 17 children (22.4%) died of their underlying condition at a median of 24 months after insertion; one died of aspiration pneumonia while awaiting fundoplication.	Favourable (no procedure-attributable deaths)	Moderate
Chao 2009 (46)	46 (39 completed, 100%)	Retrospective cohort	Mean 25 months	5 hospital deaths (12.8%), none within the 30-day post-operative period.	Favourable	Serious
Ramelli 2007 (56)	31 (31, 100%)	Longitudinal cohort	Mean 5 years	5 patients (15.6%) died of respiratory failure between 2 and 18 years after gastrostomy. None of the deaths were related to gastrostomy complications.	Favourable (no procedure-attributable deaths)	Moderate
Vajsar 2006 (46)	12 (12, 100% SMA type 1)	Retrospective case series	30 days (pre-specified)	2/12 (16.7%) died within 30 days of insertion. Causes of death were not reported and the relationship to the procedure was not assessed.	Unfavourable (highest 30-day mortality; attribution unassessed)	Not appraised
Sullivan 2006 (11)	57 (57, 100%)	Prospective longitudinal cohort	6 and 12 months	4/57 (7%) died during the study, one before gastrostomy insertion. The authors note the rate is comparable with other severely disabled populations. ^a^Same Oxford cohort as Sullivan 2005.	Favourable	Moderate/High
Craig 2006 (124)	76 (76, 100%)	Prospective cohort	6 months	3 deaths: one before consent, one from unrelated causes during follow-up and one from unrelated causes after the follow-up period.	Favourable	Moderate
Sullivan 2005 (33)	57 (57, 100%)	Prospective longitudinal cohort	12 months	2 patients died during the study, one before gastrostomy placement and one during follow-up; causes not stated. ^a^Same Oxford cohort as Sullivan 2006.	Favourable	Moderate
Harrington 2004 (88)	102 (102, 100%)	Retrospective cohort	2 years post-discharge	1/30 (3%) of the gastrostomy-alone subgroup died within 2 years; cause not specified.	Favourable	Moderate
Wales 2002 (56)	111 (111, 100%)	Retrospective cohort (fundoplication + gastrostomy vs. gastrojejunal tube)	Mean 3.1 years (GJ)/5.7 years (FG)	Mortality 12.5% (6/48) with gastrojejunal tubes versus 17.5% (11/63) with fundoplication plus gastrostomy (*p* = 0.60, NS). Reflux-related deaths accounted for 1/6 and 5/11 respectively, and included one small-bowel obstruction after fundoplication and one peritonitis following a gastrostomy tube change.	Mixed (two device-attributable deaths in the fundoplication arm)	Serious
Seguy 2002 (37)	12 (12, 100%)	Retrospective cohort	1 year	3 deaths. Two children with severe reflux died of respiratory insufficiency 10 weeks after PEG placement, aged 6 months and 3 years; a third died at 21 years from progression of motor neurone disease after 14 months of follow-up.	Unfavourable (small cohort; early respiratory deaths in the reflux subgroup)	Serious
Benoit 2001 (58)	325 (49 CP, 15%)	Retrospective chart review	≥12 months in 62%	Mortality 5/49 (10%) in the cerebral palsy subgroup against 15% for the whole cohort; the between-diagnosis difference was significant (*p* < 0.001), with the highest mortality in the cancer group. Causes and timeframe not reported.	Favourable (relative to other diagnostic groups)	Serious
Puntis 2000 (77)	29 (29, 100%)	Retrospective cohort	Median 2.6 years	5 children (17.2%) died at a mean of 8 months after PEG, from bronchopneumonia (4) and cytomegalovirus pneumonitis (1).	Mixed (respiratory deaths; not procedure-attributed)	Serious
Smith 1999 (9)	61 identified, 40 interviewed (100% CP)	Population-based retrospective cohort with vital statistics linkage	Kaplan–Meier to 10 years	16/61 (26.2%) died at a mean age of 9 years; no death was tube-feeding related. Survival was 91% at 1 year, 83% at 2, 76% at 4, 75% at 7 and 55% at 10 years.	Favourable (no procedure-attributable deaths); the longest survival curve in the review	Moderate
Arnbjörnsson 1999 (107)	98 (≈75%)	Retrospective cohort	Mean 39 months	9 patients died of their underlying disease between 2 and 16 months post-operatively.	Favourable	Serious
Tawfik 1997 (89)	29 analysed of 46 (100%)	Retrospective cohort + caregiver questionnaire	1–5 years	8/46 (17%) died within 5 years from causes not directly related to the operation.	Favourable	Moderate/High
Heine 1995 (105)	30 (30, 100%)	Prospective before/after	3–9 months (questionnaire)	7/30 (23%) died after PEG at a mean of 9.7 months (range 2–26), from chronic suppurative lung disease (6) and metastatic osteosarcoma (1). Deaths were apparently unrelated to reflux, but a falling weight-for-age *z*-score was associated with death: 4 of the 8 children whose *z*-score fell died.	Mixed (nutritional decline as a mortality marker)	Moderate
Cameron 1995 (74)	63 (63, 100%)	Retrospective cohort (PEG vs. Stamm gastrostomy)	Mean 23 months	30-day mortality 0 after PEG and 2 after Stamm gastrostomy, neither directly attributable to the gastrostomy. One-year mortality 2 after PEG and 4 after Stamm gastrostomy.	Mixed (favours PEG)	Serious
Sanders 1990 (40)	51 (51, 100% CP)	Retrospective before-after cohort	Mean 2.4 years	Mortality rose steeply with delay to intervention: 1/14 (7%) in the earliest-intervention group, 2/27 (7%) in the middle group and 4/10 (40%) in the latest group. Causes were aspiration pneumonia and surgery or infection.	Mixed (mortality rose steeply with delay to intervention)	Moderate
Stringel 1989 (91)	90 (90, 100%)	Retrospective case series	2 months–3 years	9/90 (10%) died from their neurological disorder or associated congenital anomalies between 2 months and 3 years post-operatively. No surgery-related deaths.	Favourable	Serious
Langer 1988 (55)	107 (107, 100%)	Retrospective cohort (gastrostomy ± antireflux procedure)	Mean ∼20 months	4 post-operative deaths: respiratory arrest 2 weeks post-operatively without vomiting or aspiration, narcotic overdose 6 months after a Nissen fundoplication, aspiration approximately 2 weeks post-operatively, and a blocked ventriculoperitoneal shunt 5 days post-operatively. Only 1 death was caused by reflux following gastrostomy alone.	Mixed (1 reflux-attributable death)	Serious
Khattak 1998 (79)	120 (52, 43%)	Retrospective case series	Mean 26.6 months	1 death 54 days after PEG from intra-abdominal sepsis, in an immunosuppressed post-renal-transplant patient not clearly neurologically impaired. No perioperative mortality; 6 further patients died of unrelated causes more than 6 months after PEG. ^b^Whole cohort; not NI-separable.	Mixed (1 sepsis-related death; not NI-separable)	Moderate
Wesley 1981 (90)	33 (22, 66.7%)	Retrospective case series	Mean 11 months	4/22 (18.2%) died. One early death (4.5%) on post-operative day 11 was directly procedure-related: sepsis and wound dehiscence at day 7 followed by cardiopulmonary arrest. The 3 late deaths (13.6%) were from pneumonia and were deemed unrelated to the operation.	Unfavourable (1 directly procedure-related death)	Moderate/High
Zhang 2025 (23)	84 (84, 100%)	Randomised controlled trial (PEG vs. NG)	28 days	No deaths reported; mortality was not stated as an outcome.	Not assessable	High
Wu 2026 (28)	74 (74, 100%)	Retrospective cohort (GT vs. continued NG)	Mean 43.2 days	No deaths reported; mortality was not an outcome.	Not assessable	Serious
Franken 2019 (36)	50 (39, 78%)	Prospective longitudinal cohort	3 months	No deaths during follow-up.	Favourable	Moderate
Demirbaş 2021 (44)	20 (20, 100%)	Prospective study	Mean 16.5 months	No deaths during follow-up.	Favourable	Serious
Saleem 2023 (73)	40 (40, 100%)	Prospective cohort	6 months	No deaths.	Favourable	Serious
Saadah 2009 (98)	25 (25, 100%)	Retrospective cohort	6 months	No deaths.	Favourable	Serious
Brant 1999 (13)	20 (20, 100%)	Prospective cohort	Mean 5.9 months	No deaths.	Favourable	Serious
Borowitz 1997 (66)	19 (19, 100%)	Prospective cohort	Mean 20.7 months	No deaths reported during follow-up.	Favourable	Serious
Nakao 1999 (60)	19 (16 followed, 100%)	Prospective cohort	Median 6.4 months	No deaths.	Favourable	Not appraised
Issacs 1995 (136)	22 (22, 100%)	Pre-post cohort	10 months–4 years	No deaths.	Favourable	Serious
Valletta 2004 (116)	11 (11, 100%)	Longitudinal cohort	Median 15 months	No deaths.	Favourable	Not appraised
Petersen 2006 (59)	26 (26, 100%)	Descriptive interview study	Mean 3 years 1 month	No deaths.	Favourable	Not appraised
Samuel 2016 (45)	32 (32, 100%)	Retrospective study	3 months	No child had died at the time of the study.	Favourable	High

All-cause and procedure-attributable mortality. A favourable direction denotes the absence of procedure-attributable death rather than a demonstrated survival benefit; only Lin 2021, Downs 2019, and Wong 2020 compared survival against a non-gastrostomy comparator. Attribution of cause is as stated by the original authors and was not independently adjudicated. GT, gastrostomy; PEG, percutaneous endoscopic gastrostomy; SG, Stamm gastrostomy; GJ, gastrojejunal; HR, hazard ratio; ID, intellectual disability; NI, neurological impairment.

a Companion publications reporting the same cohort.

b Data-quality flag, as noted in the cell.

#### 3.2.8 Quality of life & caregiver-reported outcomes

Quality of life is the outcome domain in which the volume of favourable reporting is least matched by the strength of the measurement behind it, and the synthesis therefore required an additional classification step before the direction of effect could be judged.

Twenty-seven studies contained a statement about quality of life. Three were set aside before classification: Sullivan et al. (33), which is not itself a quality-of-life report and refers readers to its companion paper for that outcome; Stringel (91), in which every child also had a fundoplication, so any reported change cannot be attributed to gastrostomy rather than to the combined procedure; and Barnhart et al. (104), which never measured quality of life. The remaining 24 were classified by the source of the quality-of-life claim. Seventeen (70.8%) drew on a questionnaire put to the patient or caregiver, which we designate Category A. Four (16.7%) reported a narrative account from the patient or caregiver with no questionnaire behind it, designated Category B (40, 106–108). Three (12.5%) rested on the study authors' own inference, with the claim never passing through the patient or family, designated Category C. Category C was excluded from further analysis, because treating an author's interpretation of an unrelated outcome such as weight gain as evidence of a quality-of-life benefit would inflate the apparent consistency of the reported association. The analysis set for this domain is therefore the 21 studies in Categories A and B, which between them contribute 16 child-focused results (Table 10) and 17 caregiver-focused results (Table 11).

**Table 10:** Quality of life after gastrostomy—child outcomes.

Author/Year	N (n with NI)	Design	Claim category and instrument	Finding	Direction	Risk of Bias
Franken 2020 (14)	150 (111 NI, 74%)	Cross-sectional	A—Questionnaire-based. PedsQL 4.0 Generic Core Scales	NI subgroup, post-gastrostomy only: mean total HRQoL 45.8 (SD 18.1); physical health 27.5 (SD 27.6); psychosocial health 56.8 (SD 17.2). NI children compared against neurologically normal gastrostomy children and healthy reference group	Not assessable (no pre-operative baseline)	Moderate/High
Franken 2019 (36)	50 (39 NI, 78%)	Prospective longitudinal cohort (pre- vs. post-operative)	A—Questionnaire-based. PedsQL 4.0 Generic Core Scales	Change scores: NR—mixed population, not separated [whole cohort: total HRQoL not significantly increased *p* = 0.30; psychosocial health +5.4 points *p* = 0.03, driven by social scale +10.1 points *p* = 0.04]. NI-SPECIFIC: NI children had significantly lower baseline total HRQoL than non-NI children (*p* < 0.0005); presence of NI did not influence the amount of QoL improvement gained (*p* = 0.66)	Mixed (whole-cohort; inferred—total score unchanged, psychosocial improved)	Moderate
Lefton-Greif 2011 (38)	28 (28 NI, 100%)	Retrospective review with pre/post caregiver survey	A—Questionnaire-based. Study-specific caregiver survey (no instrument named)	Caregivers reported patients had significantly more energy to participate in daily activities after GT placement (*p* < 0.0001); mealtimes reported as easier and more enjoyable for the patients	Favourable	Moderate
Tawfik 1997 (89)	29 (29 NI, 100%)	Retrospective cohort + structured caregiver questionnaire (35 items;…	A—Questionnaire-based. Study-specific 35-item caregiver questionnaire	“Child's happiness” improved in 24/29, worse in 1, no change in 4 (*p* = 0.0001 overall; *p* < 0.02 gastrostomy alone; *p* = 0.0003 gastrostomy + fundoplication). Best things about the operation (open-ended, *n* = 29, multiple answers): ease of feeding (12), abandonment of NG tube (7), weight gain (5), disappearance of aspiration (3), child's happier mood (2)	Favourable	Moderate/High
Heine 1995 (105)	30 (30 NI, 100%)	Prospective before/after (pre vs. post PEG), single-arm	A—Questionnaire-based. 42-item caregiver questionnaire	18/27 children accepted gastrostomy feeding better than previous oral/NG feeding; 3 liked it less; 6 no preference (*p* < 0.001)	Favourable	Moderate
Shapiro 1986 (94)	19 (19 NI, 100%)	Retrospective before/after (pre/post-gastrostomy growth review)	B—Narrative BY caregiver. None—narrative parent/caretaker report	Not formally measured; parents/caretakers reported that gastrostomy feeding improves the child's quality of life, with growth seen as a sign of progress	Favourable (very low certainty—narrative only)	Moderate/High
Sanders 1990 (40)	51 (51 NI, 100% CP)	Retrospective cohort, before-after (3 groups by time since CNS insult)	B—Narrative BY caregiver. None—qualitative caregiver interviews	No formal QoL measure. Caregivers reported children were more alert and less irritable after enteral feeding (qualitative)	Favourable (very low certainty—qualitative, and route not separable)	Moderate
Jennuvat 2023 (49)	109 PEG performed; 60 followed up; 39 completed…	Cross-sectional descriptive + retrospective pre/post questionnaire	A—Questionnaire-based. Study-specific non-validated Likert questionnaire	95% of parents reported improved overall quality of life for the child (non-validated Likert scale, no statistical testing)	Favourable	Moderate/High
Frediani 2015 (111)	60 (60 NI, 100% CP)	Survey (gastrostomy group A vs. oral-fed group B)	A—Questionnaire-based. CP-QOL-Child (Cerebral Palsy Quality of Life Questionnaire for Children)	Quality of life of patients in group A (gastrostomy-fed) was significantly higher than in group B (oral-fed), *p* < 0.002. Respondent (child self-report vs. parent-proxy) not stated	Favourable (between-group; not a within-child change)	*Not appraised*
Smith 1999 (9)	61 identified, 45 eligible, 40 interviewed (100% NI, all CP)	Population-based retrospective cohort + semi-structured caregiver…	A—Questionnaire-based. Semi-structured caregiver interview (not a validated instrument)	Caregiver-reported: 86% rated tube feeding as having a positive impact on the child's/family's life; 22% reported improved child mood	Favourable	Moderate/High
Clayton 2024 (88)	Record cohort 124 (124 NI, 21 with gastrostomy); survey…	Retrospective observational record review (2 centres) +…	A—Questionnaire-based. No named instrument. Survey developed by the authors with Dravet Syndrome UK (multiple…	Survey (*n* = 26 with gastrostomy): >90% of caregivers agreed their child's QoL had improved since the gastrostomy, and >90% agreed overall health was better. Record cohort: QoL improvement was one component of the composite ‘positive overall outcome’ documented in 18/21 (86%); 11/21 (52%) had improved seizure control; 15/18 in whom oral intake was permitted still took some food orally	Favourable	Moderate/High
Åvitsland 2013 (81)	58 (39 NI, 67%)	Prospective cohort	A—Questionnaire-based. Researcher-designed self-report questionnaire using 1–10 numeric rating scales, built…	Child satisfaction (parent-proxy impression of the child's well-being and happiness) improved from mean 4.97 pre-operatively to 2.81 at 18 months (*P* < .001), on a 1–10 scale where 1 = best	Favourable	Moderate
Mahant 2011 (107)	16 parents (16 children, 100% severe NI)	Qualitative, in-depth individual interviews	B—Narrative BY caregiver	Themes: physical health, emotional well-being and pain reduction, social well-being and mobility	Favourable	Low
Glasson 2020 (108)	21 caregivers (21 children, 100% intellectual/developmental…	Qualitative, semi-structured interviews with directed content analysis	B—Narrative BY caregiver. Quality of Life Inventory-Disability (QI-Disability)	Physical health domain predominated (improved general health, energy, alertness); positive emotions and independence also improved	Favourable	Low
Russell 2017 (110)	36 survey respondents (6 interviewed); 36 NI, 100% cerebral…	Mixed methods (online survey plus semi-structured phone interviews)	A—Questionnaire-based. None—qualitative (child reaction to food, emotional well-being)	Evaluated qualitatively only—child reaction to food and emotional well-being	Not assessable (no baseline)	Moderate/High (interviews) Low/Moderate (survey)
Borowitz 1997 (66)	19	Prospective cohort (pre-post)	A—Questionnaire-based. Study-specific parent rating	Parents rated overall QoL: 95% would elect PEG again, 100% would recommend it	Favourable	Serious

Claim category: A, the claim rests on a questionnaire put to the patient or caregiver; B, the claim is a narrative account from the patient or caregiver with no questionnaire behind it. Category C studies, in which the claim rests on the authors’ own inference and never passes through the patient or family, were excluded from the analysis set. PedsQL, Pediatric Quality of Life Inventory; CP-QOL-Child, Cerebral Palsy Quality of Life Questionnaire for Children; SAGA-8, Satisfaction Questionnaire with Gastrostomy Feeding; QI-Disability, Quality of Life Inventory-Disability; HRQoL, health-related quality of life; NI, neurological impairment.

**Table 11:** Quality of life after gastrostomy—caregiver outcomes.

Author/Year	N (n with NI)	Design	Claim category and instrument	Finding	Direction	Risk of bias
Lefton-Greif 2011 (38)	28 (28 NI, 100%)	Retrospective review with pre/post caregiver survey	A—Questionnaire-based. Study-specific caregiver survey (no instrument named)	Significant improvement in mealtime satisfaction; caregivers viewed tube feedings as “usually easy” to administer and were “almost always satisfied” with having the GT placed (*p* < 0.0001)	Favourable	Moderate
Sullivan 2004 (98)	57 (57 NI, 100%)	Prospective longitudinal (baseline vs. 6 and 12 months)	A—Questionnaire-based. SF-36 version II, plus carer-reported concern over nutritional status	Significant, sustained improvement across most domains at 12 months vs. baseline: Social functioning +13.51 (95% CI: 1.55 to 25.48; *p* = 0.028) Mental health +9.88 (95% CI: 2.03 to 17.72; *p* = 0.015) Energy/vitality +9.94 (95% CI: 2.29 to 17.58; *p* = 0.012) General health perception +6.35 (95% CI: 0.15 to 12.56; *p* = 0.045) Mental Component Summary +7.06 (95% CI: 0.90 to 13.22; *p* = 0.026) Most rises occurred in the first 6 months and were sustained. At 12 months physical functioning, social functioning, mental health, energy/vitality and general health perception were no longer significantly below UK population norms (Oxford Healthy Life Survey III); role limitations due to emotional problems remained…	Favourable	Moderate
Thorne 1998 (112)	62 (61 NI, ∼98%)	Retrospective cohort, single-arm	A—Questionnaire-based. Vertical visual analogue scale for device satisfaction, plus qualitative caregiver…	Overall satisfaction with the gastrostomy device consistently high (mean >80% on vertical VAS) at all follow-up points, and not associated with complication frequency. However, caregivers reported long-term gastrostomy management remained challenging—integrating feeding schedules and health routines into normal family life required adaptive coping strategies over time	Mixed (high device satisfaction alongside sustained management burden)	Serious
Tawfik 1997 (89)	29 (29 NI, 100%)	Retrospective cohort + structured caregiver questionnaire (35 items;…	A—Questionnaire-based. Study-specific 35-item caregiver questionnaire	Time to devote to other children improved in 19/26, worse in 1, no change in 6 (*p* < 0.005 overall; not significant for gastrostomy alone; *p* = 0.007 for gastrostomy + fundoplication). 24/29 wished the operation had been done earlier; 3 thought timing was right; 1 mixed; only 1 regretted it. 14/29 families were happy with support/follow-up (more support wanted from surgeon 7, nurse specialist 6, GP 6, dietician 3, neurologist 2, community nurse 1)	Favourable	Moderate/High
Heine 1995 (105)	30 (30 NI, 100%)	Prospective before/after (pre vs. post PEG), single-arm	A—Questionnaire-based. 42-item caregiver questionnaire	Family stress reduced in 9/27 families (*p* < 0.05), but increased in 3 families; 23/27 caregivers would have the PEG inserted again; main dissatisfaction was an increase in gastro-oesophageal reflux in some children	Favourable (with unfavorable elements: stress increased in 3 families, GOR dissatisfaction)	Moderate
Shapiro 1986 (94)	19 (19 NI, 100%)	Retrospective before/after (pre/post-gastrostomy growth review)	B—Narrative BY caregiver. None—narrative parent/caretaker report	Qualitative only; parents/caretakers reported improved family quality of life, and that irritability commonly decreases post-gastrostomy. No validated scale	Favourable (very low certainty—narrative only)	Moderate/High
Jennuvat 2023 (49)	109 PEG performed; 60 followed up; 39 completed…	Cross-sectional descriptive + retrospective pre/post questionnaire	A—Questionnaire-based. Study-specific non-validated Likert questionnaire	Family satisfaction 94.9%; caregivers reported improved caregiver quality of life (no instrument, no statistics)	Favourable	Moderate/High
Smith 1999 (9)	61 identified, 45 eligible, 40 interviewed (100% NI, all CP)	Population-based retrospective cohort + semi-structured caregiver…	A—Questionnaire-based. Semi-structured caregiver interview (not a validated instrument)	28% (11/40) reported family stress or functioning problems attributed to tube feeding (difficulty arranging respite care, restricted mobility, changed relationship with the child, child missing the taste of food); 86% said they would have started tube feeding earlier had they known the outcome	Mixed (majority would start earlier, but 28% reported tube-attributable family stress)	Moderate/High
Clayton 2024 (88)	Record cohort 124 (124 NI, 21 with gastrostomy); survey…	Retrospective observational record review (2 centres) +…	A—Questionnaire-based. No named instrument—same bespoke Dravet Syndrome UK survey	Survey (*n* = 26): PRE-gastrostomy 88% of caregivers were worried, with >50% endorsing concerns about the tube being pulled out, difficulty of use, infection, procedural complications, following the feeding regime, and the child stopping oral eating. POST-gastrostomy 88% were happy their child had it, concerns fell across almost all domains, and most agreed meals were less stressful and the regime easy to follow. >90% agreed it ensured medication compliance. Unmet need: 14/26 had no information beforehand, 21/26 wanted more (20/26 specifically Dravet-related). 15/26 free-text comments mostly positive; negatives concerned lack of professional support and awareness	Favourable	Moderate/High
Åvitsland 2013 (81)	58 (39 NI, 67%)	Prospective cohort	A—Questionnaire-based. Same instrument—four explicitly defined constructs: parental stress (1 = low to 10 = high),…	Parent satisfaction 5.40 to 3.50 (*P* < .001); parental stress 5.10 to 3.77 (*P* = .015); parent-child communication during meals 4.57 to 2.57 (*P* = .001), all at 18 months. MEALTIME MEASURES: meal duration did NOT decrease significantly after gastrostomy—a direct contradiction of Heine 1995, where feeding time fell substantially. Oral intake INCREASED in a notable minority (34% at 6 months, 49% at 18 months) and decreased in none.	Favourable	Moderate
O'Loughlin 2013 (43)	122 (122 NI, 100%)	Retrospective cohort with cross-sectional questionnaire survey	A—Questionnaire-based. Study-specific caregiver survey	Caregivers expressed high levels of satisfaction; only 2 of 89 (2%) regretted consenting to surgery	Favourable	Moderate/High
Mahant 2011 (107)	16 parents (16 children, 100% severe NI)	Qualitative, in-depth individual interviews	B—Narrative BY caregiver	Themes: intense caregiving needs, sleep deprivation, social isolation, financial strain, finding meaning	Mixed (benefits described alongside sustained caregiving burden)	Low
Demirbaş 2021 (44)	20 (20 NI, 100%)	Prospective study	A—Questionnaire-based (weak). Study-specific family satisfaction question	Family satisfaction 10/11 (90.9%) in Group 1 and 8/9 (88.8%) in Group 2	Favourable	Serious
Glasson 2020 (108)	21 caregivers (21 children, 100% intellectual/developmental…	Qualitative, semi-structured interviews with directed content analysis	B—Narrative BY caregiver. Beach Center Family Quality of Life Scale	Improved family interaction, parenting, emotional well-being, and resources/supports	Favourable	Low
Russell 2017 (110)	36 survey respondents (6 interviewed); 36 NI, 100% cerebral…	Mixed methods (online survey plus semi-structured phone interviews)	A—Questionnaire-based. SAGA-8	SAGA-8: 50% expressed a high degree of satisfaction with gastrostomy feeding; 31% reported improvement in family life and 42% indicated significant improvement	Favourable	Low/Moderate (interviews) Moderate/High (Survey)
Koca 2015 (68)	34 children (47 PEG procedures); NI predominant -…	Retrospective study	A—Questionnaire-based. Study-specific parent questionnaire	All parents of the 20 children still followed with a PEG said they would choose PEG placement again	Favourable	Serious
Borowitz 1997 (66)	19	Prospective cohort (pre-post)	A—Questionnaire-based. Study-specific parent report	90% (17/19) of families felt the PEG made their lives less stressful	Favourable	Serious

Categories as for Table 10. SF-36, 36-Item Short Form Health Survey; VAS, visual analogue scale. Sullivan 2004 and Sullivan 2005 report the same Oxford Feeding Study cohort, and the record and survey subgroups of Clayton 2024 may overlap by the authors’ own statement, so the 21 studies in the analysis set are not 21 independent cohorts. Three studies appear in both Tables 10, 11 with different findings, since they report a child and a caregiver outcome separately.

#### 3.2.9 Child quality of life

Among the 16 studies reporting a child outcome we classified the reported direction of change as favourable in 13, mixed in one (109), and not assessable in two for want of a baseline (14, 110). No study reported an unfavourable direction of change in either group. Because no included study was randomised or controlled, these are reported directions of change and not estimates of an effect of gastrostomy, and the count cannot be read at face value. Only one record is robust, scoring 11 of 12 because it alone combined a validated instrument, a prospective pre-operative baseline and a directly measured quality-of-life endpoint (109), and its graded direction is mixed rather than favourable. Of the three records of intermediate strength, meaning they satisfy most of the rubric but fail at least one of those three requirements, the highest-scoring is cross-sectional and therefore not estimable for want of a baseline (11 of 12) (14). The remaining twelve rows are weaker still: six of limited strength, typically with no validated instrument and a pre-operative state reconstructed from memory; three anecdotal, with no instrument, no numbers and no statistical testing; two qualitative and therefore ungraded; and one available only as a conference abstract, whose methods cannot be appraised at all. It is these weaker records that carry almost all of the favourable signal, in most cases with the pre-operative state reconstructed by the same respondent who supplied the post-operative rating.

The instruments behind these results are weak. Within the 17 Category-A studies only four (23.5%) used a validated instrument, three of them for the child outcome: the parent-proxy PedsQL 4.0 (14, 109) and CP-QOL-Child [Frediani (111), available only as an abstract]. For the child, only two studies applied a validated instrument, only one of which had a baseline against which change could be measured, and that study found no significant change in total health-related quality of life (*p* = 0.30), with improvement confined to the psychosocial summary (+5.4 points, *p* = 0.03) and driven by the social scale (+10.1 points, *p* = 0.04); the improvement was not modified by neurological impairment status (*p* = 0.66), but the change scores are reported for the whole mixed cohort rather than the impaired subgroup. Frediani (111) found significantly higher CP-QOL-Child scores in gastrostomy-fed than orally fed children (*p* < 0.002), but this is a between-group comparison in an abstract-only report rather than a within-child change. No study obtained child self-report.

Weighting therefore reverses the impression given by counting: the only record capable of supporting a moderate-certainty statement is mixed, so the child outcome is best described as no demonstrated change in measured health-related quality of life, alongside consistent caregiver-reported improvement in the child's well-being at low to very low certainty. At outcome level, certainty that gastrostomy is associated with a measured change in the child's own health-related quality of life is very low: the body of evidence is inconsistent between its single interpretable record and the remainder, imprecise, indirect in that the endpoint is proxy-reported throughout, and incompletely appraised for risk of bias.

#### 3.2.10 Caregiver quality of life

Among the 17 reporting a caregiver outcome we classified 14 as favourable and three as mixed (9, 107, 112). Here the weighted picture is more favourable, because the single robust record falls on the favourable side: Sullivan (113) scores 12 of 12, the only full-mark record in either table, on a validated SF-36 version 2 administered prospectively with a pre-operative baseline. Of the three records of intermediate strength, each prospectively measured but relying on a purpose-built instrument or a proxy endpoint rather than a validated quality-of-life measure, one is favourable [Avitsland (81), 9 of 12] and two are graded mixed [Heine (105), 8 of 12; Thorne (112), 7 of 12]. The remaining thirteen rows are weaker: seven of limited strength, generally lacking a validated instrument and reconstructing the pre-operative state by recall; four anecdotal, with no instrument and no statistical testing; and two qualitative and therefore ungraded.

The fourth validated administration is the caregiver's own SF-36 version 2 (113). The remaining 13 (76.5%) of the Category-A instruments were purpose-built for the study in question, most commonly a researcher-developed multi-item questionnaire (49, 72, 89, 105), and otherwise a Likert-agreement survey (Clayton 2024), numeric rating scales (81), a visual analogue scale (112), or a named satisfaction instrument, SAGA-8 (110), which measures gastrostomy-specific satisfaction rather than general quality of life. Two further studies used validated frameworks (QI-Disability and the Beach Center Family Quality of Life Scale) to code qualitative interview transcripts rather than administering them as scored questionnaires (108), which strengthens the analysis but produces no scores, baselines or statistics. The domains tested are correspondingly narrow. Caregiver satisfaction was the commonest domain, appearing in 12 of the 21 studies, against only three studies that tested the validated physical, social, emotional and pain constructs that make up a generic health-related quality-of-life instrument.

For the caregiver, the strongest quantitative result in the whole review is available: Sullivan et al. (113) recorded significant and sustained SF-36 gains at 12 months in social functioning (+13.51, 95% CI: 1.55 to 25.48, *p* = 0.028), mental health (+9.88, 95% CI: 2.03 to 17.72, *p* = 0.015), energy and vitality (+9.94, 95% CI: 2.29 to 17.58, *p* = 0.012), general health perception (+6.35, 95% CI: 0.15 to 12.56, *p* = 0.045) and the mental component summary (+7.06, 95% CI: 0.90 to 13.22, *p* = 0.026), with most of the gain occurring in the first six months and sustained thereafter. By 12 months most domains were no longer significantly below UK population norms, and caregiver worry about the child's nutritional state fell from 78% before gastrostomy to 15% at 12 months (*p* < 0.0001). Åvitsland et al. (81) provides the next-best-quantified dataset, with prospective measurement at baseline, 6 and 18 months showing improvement in parent satisfaction (5.40 to 3.50, *p* < 0.001), parental stress (5.10 to 3.77, *p* = 0.015), parent-child communication during meals (4.57 to 2.57, *p* = 0.001) and proxy-rated child well-being (4.97 to 2.81, *p* < 0.001), on scales where a lower score is better.

Two qualifications run through the caregiver evidence. The first is that satisfaction measures acceptability rather than quality of life. When Borowitz et al. (72) report that 95% of parents would elect gastrostomy again and 100% would recommend it, or Koca et al. (74) that all 20 responding families would choose PEG again, that is a statement about whether families were glad they did it, not a measurement of what their quality of life became. The second is that where burden was measured rather than satisfaction, the picture is less uniform. Thorne et al. (112) found device satisfaction consistently above 80% on a visual analogue scale and unrelated to complication frequency, yet caregivers described long-term management as a continuing challenge requiring adaptive coping. Smith et al. (9) found that 86% of caregivers rated tube feeding as having a positive impact on the child's and family's life but that 28% reported tube-attributable family stress including difficulty arranging respite, restricted mobility and a changed relationship with the child. Mahant et al. (107) describes intense caregiving needs, sleep deprivation, social isolation and financial strain alongside the benefits. Avitsland et al. (81) found that meal duration did not fall significantly after gastrostomy, which directly contradicts Heine et al. (105), and that oral intake increased in a notable minority (34% at 6 months, 49% at 18 months) and decreased in none. A third qualification follows from the appraisal: where a published instrument replaced a bespoke one the favourable signal attenuated sharply, Russell (110) recording high satisfaction in only 50% of caregivers on SAGA-8 against 90 to 100% on the older bespoke surveys, and the most credible estimate of the unfavourable minority comes from the strongest sampling frame in the review, Smith's (9) population-based 28%.

Because one robust favourable record outweighs a larger number of limited and anecdotal records, the caregiver outcome is graded favourable at moderate certainty, with an unfavourable minority that is consistently present and consistently small. At outcome level, certainty that gastrostomy is associated with improved caregiver-reported quality of life is moderate, and is held below high by the absence of any controlled comparison, by the graded estimate resting on a single record, and by incomplete risk-of-bias appraisal across the remainder; the available evidence supports an association and not a demonstrated effect.

Limitations of the weighted result. Three features of the weighting limit what these results can be taken to show. First, the weight is concentrated rather than distributed: one record per outcome carries the only moderate-certainty statement available, so both conclusions rest on a single study each rather than on the 33 rows counted, and revision of either record's strength category would change the reported direction. Second, the weighting axis is the interpretability of each study's quality-of-life measurement rather than a complete appraisal of internal validity, because risk of bias is documented for only two of the records in these tables; a record can therefore be weighted highly here and remain at unappraised risk of bias. Third, the strength categories and certainty grades involved reviewer judgement at the margins, were performed by a single reviewer against extracted evidence, and await second-reviewer verification. The direction reported for each outcome is accordingly a weighted and partly subjective judgement about a body of uncontrolled evidence: it can suggest an association between gastrostomy and reported quality of life, but it cannot establish that gastrostomy caused the change.

Study-level data for this domain are presented in Table 10 (child outcomes) and Table 11 (caregiver outcomes).

### 3.3 Assessment of risk of bias

One hundred and two of the included studies were formally appraised. The remaining eighteen were conference abstracts, excluded from formal critical appraisal as they do not provide sufficient methodological detail to be reliably scored. Study-level judgements for every domain of every tool are given in Tables 12a–d, split by the instrument applied: RoB 2 for the single randomised trial (Table 12a), ROBINS-I for the non-randomised comparative and cohort studies (Table 12b), the JBI critical appraisal checklists for cross-sectional, survey and questionnaire-based studies (Table 12c), and the CASP qualitative checklist for the interview studies (Table 12d). Conference abstracts (*n* = 16) were excluded from formal critical appraisal, as they do not provide sufficient methodological detail to be reliably scored.

**Table 12a:** Risk of bias assessment of the included studies: RoB 2—randomised trial.

Study	D1: Randomization	D2: Deviations	D3: Missing data	D4: Measurement	D5: Reported result	Overall risk
Zhang, 2025 (27)	High	High	Low	Some Concerns	Some Concerns	High

**Table 12b:** Risk of bias assessment of the included studies: ROBINS-I—non-randomised comparative and cohort studies.

Study	D1: Confounding	D2: Selection	D3: Classification	D4: Deviations	D5: Missing data	D6: Measurement	D7: Reported result	Overall risk
Guillén Redondo, 2021 (82)	Serious	Moderate	Low	Low	Moderate	Moderate	Low	Serious
Ulman, 2021 (101)	Moderate	Moderate	Low	Low	Low	Low	Low	Moderate
Zambaiti, 2021 (100)	Serious	Moderate	Low	Low	Low	Low	Low	Serious
Wong, 2020 (87)	Low/Moderate	Low	Low	Low	Low	Low	Low	Low/Moderate
Viswanath, 2010 (61)	Serious	Moderate	Low	Low	Low	Low	Low	Serious
Sullivan, 2006 (Comp) (32)	Serious	Moderate	Low	Low	Moderate	Low	Low	Serious
Wales, 2002 (84)	Serious	Moderate	Low	Low	Low	Low	Low	Serious
Langer, 1988 (83)	Serious	Moderate	Low	Low	Moderate	Low	Low	Serious
Nelson, 2019 (57)	Moderate	Low	Low	Low	Low	Low	Low	Low/Moderate
Lin, 2021 (121)	Moderate	Low	Low	Low	Low	Moderate	Low	Moderate
Barnhart, 2013 (104)	Moderate	Low	Low	Low	Moderate	Moderate	Low	Moderate
Fascetti-Leon, 2012 (76)	Moderate	Low	Low	Low	Moderate	Moderate	Low	Moderate
Dreznik, 2022 (80)	Serious	Moderate	Low	Low	Moderate	Moderate	Low	Serious
AlNamshan, 2019 (55)	Serious	Moderate	Low	Low	Moderate	Low	Low	Serious
Koca, 2015 (74)	Serious	Moderate	Low	Low	Serious	Low	Moderate	Serious
Arrowsmith, 2010 (38)	Serious	Moderate	Low	Low	Moderate	Low	Low	Serious
Catto-Smith, 2006 (35)	Serious	Moderate	Low	Low	Moderate	Low	Low	Serious
Arnbjörnsson, 1999 (125)	Serious	Moderate	Low	Low	Moderate	Moderate	Moderate	Serious
Brant, 1999 (13)	Serious	Moderate	Low	Low	Low	Low	Moderate	Serious
Borowitz, 1997 (72)	Serious	Moderate	Low	Low	Low	Serious	Moderate	Serious
Cameron, 1995 (62)	Serious	Moderate	Low	Low	Moderate	Moderate	Low	Serious
Stringel, 1989 (91)	Serious	Moderate	Serious	Low	Serious	Moderate	Moderate	Serious
Işık, 2021 (68)	Serious	Moderate	Low	Low	Moderate	Moderate	Low	Serious
Lertudomphonwanit, 2019 (59)	Serious	Moderate	Low	Low	Moderate	Low/Moderate	Low	Serious
Kehar, 2014 (54)	Serious	Serious	Low	Low	Moderate	Low	Moderate	Serious
Saadah, 2009 (98)	Serious	Moderate	Low	Low	Serious	Moderate	Moderate	Serious
Motil, 2009 (36)	Serious	Moderate	Low	Low	Moderate	Low	Low	Serious
Arnbjörnsson, 2006 (67)	Serious	Moderate	Low	Low	Moderate	Low	Low	Serious
Seguy, 2002 (37)	Serious	Moderate	Low	Low	Moderate	Low	Moderate	Serious
Benoit, 2001 (64)	Serious	Moderate	Moderate	Low	Serious	Moderate	Moderate	Serious
Saleem, 2023 (73)	Serious	Moderate	Low	Low	Low	Low	Low	Serious
Park, 2011 (78)	Serious	Moderate	Low	Low	Moderate	Moderate	Low	Serious
Ramelli, 2007 (56)	Serious	Moderate	Low	Low	Serious	Moderate	Moderate	Serious
Issacs, 1995 (136)	Serious	Serious	Low	Low	Moderate	Low/Moderate	Moderate	Serious
Puntis, 2000 (77)	Serious	Serious	Low	Low	Moderate	Moderate	Low	Serious
Ercoli, 2017 (128)	Serious	Moderate	Low	Low	Moderate	Moderate	Moderate	Serious
Jadi, 2023 (71)	Serious	Moderate	Low	Moderate	Serious	Moderate	Moderate	Serious
Demirbaş, 2021 (44)	Serious	Moderate	Low	Low	Low	Low	Moderate	Serious
Machado, 2016 (94)	Serious	Serious	Low	Moderate	Moderate	Low	Moderate	Serious
Wu 2026 (28)	Serious	Moderate	Low	Moderate	Moderate	Moderate	Moderate	Serious
Tarek 2024 (92)	Serious	Moderate	Low	Moderate	Low	Moderate	Moderate	Serious
Martínez-Costa 2019 (6)	Serious	Moderate	Low	Low	Moderate	Low	Low	Serious
Chao 2009 (46)	Serious	Moderate	Low	Moderate	Moderate	Low	Low	Serious
Wong 2019 (86)	Moderate	Moderate	Low	Low	Moderate	Low	Low	Moderate
Thorne 1998 (112)	Moderate	Moderate	Low	Moderate	Serious	Moderate	Low	Serious
Sullivan 2005 (33)	Serious	Moderate	Low	Low	Moderate	Low	Low	Serious
Civan 2021 (120)	Serious	Moderate	Low	Low	Moderate	Low	Low	Serious
Pinheiro 2025 (42)	Serious	Moderate	Low	Low	Serious	Moderate	Low	Serious
Franken 2020b (47)	Serious	Moderate	Low	Low	Serious	Moderate	Low	Serious

**Table 12c:** Risk of bias assessment of the included studies: JBI critical appraisal—cross-sectional, survey and questionnaire-based studies.

Study	Sample selection & similarity	Exposure measurement	Confounding control	Outcome measurement	Follow-up adequacy	Overall risk
Grayeb, 2025 (39)	Low	Low	Low	Low	Low	Low
Clayton, 2024 (88)	Low	Moderate	Moderate	High	Moderate	Moderate/High
Dipasquale, 2023 (8)	Low	Low	Moderate	Moderate	Low	Moderate
Marpole, 2022 (118)	Low	Low	High	Low	Moderate	Moderate
Wadman, 2021 (99)	High	Low	Moderate	Moderate	Moderate	Moderate/High
Sakamoto, 2021 (69)	Low	Low	Moderate	Moderate	Moderate	Moderate
Masui, 2022 (119)	Moderate	Low	Moderate	Low	Low	Moderate
Williams, 2023 (102)	Low	Moderate	Low	Low	Low	Low/Moderate
Figueiredo, 2020 (115)	High	Low	High	Low	N/A	Moderate/High
Suh, 2020 (7)	Moderate	Low	Moderate	Low	Low	Moderate
Jacoby, 2020 (50)	Low	Low	Low	Low	Low	Low
Franken, 2019 (109)	Moderate	Low	Moderate	Low/Moderate	Moderate	Moderate
Franken, 2020a (14)	Moderate/High	Low	Moderate	Low/Moderate	N/A	Moderate/High
Aumar, 2018 (63)	Low	Low	Low	Moderate	Low	Low/Moderate
Figueroa, 2017 (29)	Moderate	Low	High	Low	Low	Moderate
Åvitsland, 2013 (81)	Moderate	Low	Moderate	Moderate	Moderate	Moderate
Franken, 2015 (34)	Low	Low	Moderate	Moderate	Low	Moderate
Sharma, 2012 (123)	Low	Low	High	Moderate	Moderate	Moderate/High
Kawahara, 2014 (96)	Moderate	Low	Moderate	Low	Low	Moderate
Caselli, 2017 (24)	Moderate	Low	High	Moderate	N/A	Moderate/High
Toporowska-Kowalska, 2011 (95)	Moderate	Low	Moderate	Low	Low	Moderate
Lefton-Greif, 2011 (48)	Low	Low	Moderate	Low	Low	Moderate
Thomson, 2011 (53)	Moderate	Low	High	Low	Low	Moderate/High
Kawahara, 2010 (97)	Moderate	Low	Moderate	Low	Moderate	Moderate
Dahlseng, 2012 (114)	Low	Low	Low	Low	N/A	Low
O'Loughlin, 2013 (43)	Moderate	Low	High	High	Moderate	Moderate/High
Day, 2007 (25)	Low	Low	Low	Low	Low	Low
Craig, 2006 (124)	Moderate	Low	Moderate	Moderate	Moderate	Moderate
Sullivan, 2004 (113)	Moderate	Low	Moderate	Low	Moderate	Moderate
Sathesh-Kumar, 2009 (70)	Moderate	Low	High	Low	Low	Moderate/High
Sullivan, 2006 (Resp) (111)	Moderate	Low	High	Moderate	Moderate	Moderate/High
Harrington, 2004 (103)	Moderate	Low	Moderate	Low	Moderate	Moderate
Smith, 1999 (9)	Low	Low	High	Moderate/High	Moderate	Moderate/High
Khattak, 1998 (79)	Moderate	Low	High	Low	Moderate	Moderate
Tawfik, 1997 (89)	Moderate	Low	High	High	N/A	Moderate/High
Heine, 1995 (105)	Moderate	Low	High	Moderate	Moderate	Moderate
Sanders, 1990 (40)	Moderate	Low	Moderate	Low	Moderate	Moderate
Noviello, 2021 (75)	Moderate	Low	High	Low	Moderate	Moderate
Shapiro, 1986 (106)	High	Low	High	Low	Moderate	Moderate/High
Wesley, 1981 (90)	High	Low	High	Low	Moderate	Moderate/High
Stewart, 2017 (85)	Low	Low	High	Low	Low	Moderate
Finbråten, 2011 (30)	Low	Moderate	Moderate	Low	N/A	Moderate
Toporowska-Kowalska, 2013 (132)	Moderate	Low	Moderate	Moderate	Moderate	Moderate
Russell, 2017 (survey) (110)	Moderate	Low	High	Moderate	N/A	Moderate/High
Jennuvat, 2023 (49)	High	Low	High	Moderate	Moderate	Moderate/High
Samuel 2016 (45)	Moderate	Low	High	Moderate	Moderate	High
Tavares de Sousa 2020 (31)	Moderate	Moderate	High	Low	N/A	Moderate
Backman et al., 2014 (122)	Low	Low	Moderate/High	Moderate	Low	Moderate
Toporowska-Kowalska et al., 2011 (95)	Moderate	Low	Moderate	Low	Low	Moderate
Petersen et al., 2006 (65)	Moderate	Low	High	High	N/A	Moderate/High

**Table 12d:** Risk of bias assessment of the included studies: CASP qualitative checklist—interview studies.

Study	Aims/Method	Recruitment	Data collection	Researcher Rel.	Data analysis	Overall risk
Mahant, 2011 (107)	Low	Low	Low	Low	Low	Low
Glasson, 2020 (108)	Low	Low	Low	Moderate	Low	Low
Russell, 2017 (interviews) (110)	Low	Moderate	Moderate	Low	Low	Low/Moderate

D1 to D7 are the domains of the tool named in each sub-table heading. RoB 2 domains: D1 randomisation process, D2 deviations from intended intervention, D3 missing outcome data, D4 measurement of the outcome, D5 selection of the reported result. ROBINS-I domains: D1 confounding, D2 selection of participants, D3 classification of interventions, D4 deviations from intended interventions, D5 missing data, D6 measurement of outcomes, D7 selection of the reported result. Where a study contributed to more than one outcome domain and was appraised separately for each, the outcome is given in parentheses after the study name.

Overall methodological quality was low, and the weakness was systematic rather than idiosyncratic. Of the 49 studies appraised with ROBINS-I, 38 (86.4%) were rated at serious overall risk of bias, four at moderate and two at low to moderate. Not one reached an overall low rating. The domain responsible is almost always the same: confounding was rated serious in the great majority of these studies, and this is intrinsic to the field rather than a failure of individual investigators. Children are selected for gastrostomy because their feeding is already failing and their impairment is most severe, so any comparison against children who did not receive one is confounded by the reason the decision was made. Selection bias was rated moderate in nearly every ROBINS-I study for the related reason that cohorts were assembled from those who had already undergone the procedure. By contrast, the domains under the investigators' direct control performed well: classification of the intervention and deviations from intended intervention were rated low in almost every study, since gastrostomy placement is an unambiguous, well-documented exposure.

The two studies with the lowest ROBINS-I risk are the two population-linked administrative analyses, Nelson et al. and Wong et al. (57, 86) both rated low to moderate, and the next best are Lin et al. (12), Barnhart et al. (104), Ulman et al. (101) and Fascetti-Leon et al. (76) at moderate. This matters for interpretation rather than as a bookkeeping observation, because these are the same studies that most often disagree with the aggregate direction of effect in the respiratory and mortality domains. Where the highest-quality evidence and the majority vote diverge, the divergence tracks study quality.

Among the 50 studies appraised with the JBI checklists, four were rated low risk overall (25, 39, 50, 114), two low to moderate, 27 moderate and 17 moderate to high. The recurrent weaknesses here were confounding control and outcome measurement rather than sampling. Studies rated moderate to high were characteristically those relying on retrospective caregiver recall of the pre-gastrostomy state collected at the same sitting as the post-gastrostomy opinion (9, 43, 49, 89, 110), those reporting only cross-sectional status with no baseline (14, 24, 115), and the abstract-only reports in which the methods could not be checked against the results (111).

The single randomised trial, Zhang et al. (27), was rated at high overall risk of bias on RoB 2, with high risk in both the randomisation and deviations domains and some concerns for outcome measurement and selective reporting. This is consequential out of proportion to the study's size, because it is the only randomised evidence in the entire review and it is cited in the nutritional, respiratory, reflux and major-complication domains. Its own journal labels it an observational study despite a described random-number-table allocation, which is consistent with the randomisation-domain judgement. Its results should be treated as supportive rather than confirmatory.

The three qualitative studies performed best of any group against their own instrument. Nelson et al. (57) and Glasson et al. (108) were rated low risk on the CASP checklist and Russell et al. (110) low to moderate, with the only concerns being researcher reflexivity and recruitment. This does not make their findings quantitatively poolable, and they are not used that way in this review, but it does mean the caregiver-burden narrative they contribute is methodologically sound on its own terms.

Several cross-cutting reporting weaknesses affect the appraisal irrespective of tool. Functional severity was rarely classified with a standardised instrument such as the GMFCS, so residual confounding by impairment severity cannot be excluded even where confounding was otherwise addressed. Many studies grouped heterogeneous neurological diagnoses into a single composite category without disaggregating by aetiology, and several reported complication, growth, reflux and mortality outcomes for whole mixed cohorts rather than stratified by neurological-impairment status, which dilutes the estimates specific to this population. The studies drawing on administrative or claims data (50, 102) trade clinical granularity for scale and carry the misclassification risk inherent in diagnostic and procedural coding. Attrition was a specific concern in three cohorts: Martínez-Costa et al. (6) excluded 33 of 98 recruited children, mainly for missing anthropometric data; Pinheiro et al. (42) excluded 5 of 23 operated children including one death; and Kaur et al. (93) lost 16 children to follow-up without a stated follow-up duration. Where attrition is concentrated among sicker children, as is plausible in this population, the reported outcomes describe a healthier surviving subset and are likely to overstate benefit. Martínez-Costa's exclusions are documented and reasoned, which makes Kaur's unexplained loss the more confidence-reducing of the two.

Cohort size compounds these issues in a subset of studies. Shapiro (*n* = 19) (106), Pinheiro et al. (*n* = 18) (42), Brant (*n* = 20) (13), Thomson et al. (*n* = 10) (53), Toporowska-Kowalska et al. (*n* = 15) (95) and Valletta et al. (*n* = 11) (116) each enrolled fewer than 25 children. In cohorts this small a single atypical case moves a reported percentage substantially, so their point estimates are less stable than those from the larger cohorts, and the synthesis weights them accordingly.

The overall picture is an evidence base in which the exposure is well defined and well documented, the outcomes are variably defined and often retrospectively ascertained, and confounding by indication is pervasive and largely unaddressed. Eighty-six per cent of the comparative studies carry a serious risk of bias, no comparative study reaches low risk, and the only randomised trial is at high risk. Every directional conclusion in this review should be read against that background.

## 4 Discussion

### 4.1 Summary of main findings

This systematic review of 120 independent studies reveals a stark contrast between the well-documented nutritional and caregiver benefits of gastrostomy, and its unexpectedly unproven effects on respiratory health and child neurodevelopment. By synthesizing data spanning over four decades, it becomes clear that while gastrostomy is consistently associated with resolving the narrow endpoint of malnutrition, its broader systemic impacts require a highly nuanced clinical approach.

The most definitive benefit of gastrostomy placement is rapid nutritional recovery, supported by moderate certainty evidence. The majority of included cohorts demonstrated highly significant improvements in weight-for-age and body mass index (BMI) *z*-scores following tube placement. For example, prospective data highlighted a median weight-for-age *z*-score improvement from −3.03 to −1.60 within 12 months, resulting in robust catch-up growth. Crucially, the literature demonstrates a distinct dose-response relationship between the timing of the intervention and the speed of recovery; children treated earliest after neurological insult experience weight-gain velocities more than double those treated later (3.3 g/kg/day vs. 1.5 g/kg/day), alongside higher rates of full nutritional normalization. This aligns closely with broader international consensus, including the 2019 ESPGHAN guidelines and the Cochrane review of gastrostomy in cerebral palsy (16), which similarly identified weight gain as the most consistent benefit across literature. While these findings suggest that earlier placement, before prolonged undernutrition occurs, is associated with more robust catch-up growth, confounding by indication and severity cannot be excluded. The evidence supports offering gastrostomy without undue delay, though it does not establish a causal effect of timing alone.

Although gastrostomy is often used in practice to help prevent aspiration, the highest-quality data provides moderate-certainty evidence that it does not actually offer respiratory protection. While smaller, uncontrolled caregiver-reported cohorts frequently cite reductions in chest infections, large population-linked and propensity-matched datasets repeatedly contradict this. Population analyses have demonstrated that lower-respiratory-tract infection admissions often rise or remain unchanged post-gastrostomy (with incidence rate ratios remaining elevated up to six years post-placement), and propensity-matched data even indicated an increased hazard ratio (HR: 1.21) for severe pneumonia hospitalizations. This difference between study types is likely due to confounding by indication. In practice, the sickest children with the highest risk of aspiration are the ones most likely to receive a gastrostomy, which biases observational studies to make the procedure look harmful for respiratory health. When higher-quality studies adjust for this baseline severity using propensity matching, the apparent respiratory benefits disappear. This is consistent with the Cochrane review by Gantasala et al. (16), which also found conflicting evidence on respiratory illness. Ultimately, while gastrostomy bypasses oral feeding issues, it cannot prevent respiratory problems caused by salivary aspiration or poor airway clearance.

While the review includes various insertion techniques (e.g., percutaneous endoscopic, laparoscopic, and open surgical gastrostomy), comparative data isolating the specific effect of insertion technique on long-term outcomes remain sparse. Surgically, gastrostomy placement alone appears to be relatively safe (supported by low-to-moderate-certainty evidence), with major complications (such as peritonitis or colonic injury) and procedure-attributable mortality occurring in roughly 0% to 3% of cases. However, serious morbidity rises several-fold when a concurrent antireflux procedure (fundoplication) or a jejunal extension is added. Interestingly, broader recent literature supports a conservative approach to concurrent surgeries; a 2021 retrospective cohort study published in MDPI found that neurologically impaired children receiving gastrostomy alone actually experienced significantly fewer long-term reflux-related hospitalizations per year than those receiving a concurrent fundoplication, although the observational design limits causal interpretation. Within this review, physiological studies offer a possible explanation for the heterogeneous reflux outcomes observed across studies: gastrostomy was associated with *de novo* reflux in children who previously had none, while improvement in reflux indices was observed in a proportion of those with pre-existing disease. Although these findings were broadly consistent across several studies, confidence is limited by the predominantly observational evidence and moderate-to-serious risk of bias. Given that subsequent antireflux surgery was generally required in fewer than 10% of children, the available evidence does not support routine concurrent fundoplication in the absence of a clear clinical indication. The American Academy of Pediatrics (AAP) notes that adding a G-J tube or fundoplication increases the risk of surgery-related issues like bleeding and infection, as well as long-term risks like overfeeding and worsening reflux (117). Because of these risks, concurrent procedures should be reserved for children with severe vomiting, persistent aspiration, or highly symptomatic GERD. This cautious approach is also supported by recent Cochrane reviews on managing GERD in children with neurological impairments (17).

For families, gastrostomy significantly reduces caregiver burden (moderate-certainty evidence). Utilizing validated tools like the SF-36, studies have documented sustained improvements in caregiver mental health, vitality, and social functioning, alongside a massive drop in nutrition-related anxiety (falling from 78% pre-operatively to 15% at 12 months). However, this clear benefit for caregivers contrasts sharply with the lack of data on the child's neurodevelopment, where we found very low-certainty to no evidence. Across 120 studies, only one applied a standardized cognitive instrument (the WeeFIM scale), finding that developmental improvements were tied to natural rehabilitation rather than the feeding route itself. No robust evidence exists proving gastrostomy directly improves cognition, gross motor function, or communication.

Ultimately, synthesizing these discrete clinical trade-offs will be highly useful to help physicians and parents make a shared, evidence-based decision about whether gastrostomy will be beneficial for a child. By replacing generalized assumptions of respiratory protection with concrete data on nutritional timing and surgical safety, clinicians can guide families through a much more transparent and accurate consent process.

### 4.2 Limitations

The limitations of this review fall into three groups: limitations of the evidence, limitations of the review process, and limitations of the synthesis method.

The evidence itself is the principal constraint, and the formal appraisal quantifies it: 86% of the comparative studies assessed with ROBINS-I carry a serious overall risk of bias, no comparative study reaches low risk, and the only randomised trial is at high risk. Only one randomised comparison exists, its design classification is disputed by its own journal, and its follow-up is 28 days. The remainder is observational, and most of it is uncontrolled. Confounding by indication is unavoidable and largely unaddressed: gastrostomy is offered to the children who are least well, so any comparison with children who did not receive one is confounded by the severity that prompted the decision, and few studies adjusted for impairment severity or recorded GMFCS level at all. Outcome definitions are inconsistent to a degree that limits comparison across studies, particularly for chest infection, aspiration and minor complication. Follow-up is short in a large proportion of studies, which matters most for the outcomes that develop slowly, including linear growth, adiposity and buried bumper syndrome. Several cohorts report mixed populations in which the neurologically impaired subgroup is not separable, and where this is the case the finding is flagged in the relevant table and should not be read as neurology-specific. Two domain-specific constraints deserve separate mention. In the reflux domain, most studies report a single cohort-wide incidence without stratifying by pre-operative reflux status, which, given that the effect runs in opposite directions in the two groups, makes those incidences difficult to interpret and impossible to compare across studies with different baseline reflux prevalence. In the quality-of-life domain, almost every result is proxy-reported by a caregiver or clinician rather than by the child, most non-validated instruments ask families to recall the pre-gastrostomy state at the same sitting as they give their post-gastrostomy opinion, and the counts of studies are not counts of independent cohorts, since two reports describe the same Oxford Feeding Study cohort and one study's record and survey subgroups may overlap by its own authors' statement. In the mortality domain, all-cause mortality is reported far more often than procedure-attributable mortality, attribution is made by the original authors rather than by any independent adjudication, and only three studies had a non-gastrostomy comparator, of which one found reduced short-term mortality and one found increased mortality, so the absence of procedure-attributable death across this literature is reassuring but not equivalent to a demonstrated absence of excess risk. Older series describe surgical and perioperative practice that has since changed, and cohorts recruited over periods of up to two decades are additionally vulnerable to secular change in surgical technique and perioperative care within a single study. Functional severity was rarely classified with a standardised instrument such as the GMFCS, and several studies grouped heterogeneous neurological diagnoses into one composite category without disaggregating by aetiology, so residual confounding by severity and by diagnosis cannot be excluded. Reporting bias is likely and could not be assessed formally: complication data in particular are more likely to be reported when notable, and several studies recorded no complications in a manner that does not distinguish absence of events from absence of reporting.

The review process has its own limitations. The search was restricted to five sources and grey literature was not searched systematically beyond conference abstracts retrieved by those searches, so eligible unpublished or non-indexed studies may have been missed. Non-English publications were excluded at full-text screening, and 15 otherwise potentially eligible reports were lost on that basis, which risks language bias in a field with substantial European, Latin American and East Asian output. Screening was duplicated throughout by two independent reviewer teams, but extraction was distributed across five reviewers rather than duplicated, so single-extractor error is possible. Two instances found during central checking illustrate the risk: one included study reports individual complication rates that sum to more than its own stated total, and one Covidence record was extracted twice with directly contradictory mortality data that could not be reconciled, so that study is not counted in either direction in the mortality synthesis. Cohort overlap was identified from institution, period, sample size and authorship rather than by contacting authors, so residual double-counting cannot be excluded, and one included study explicitly reports two subgroups whose overlap the original authors state cannot be quantified. Study authors were not contacted for missing or ambiguous data. Risk of bias was assessed for 102 of the 120 included studies, with 18 being conference abstracts and therefore not eligible for appraisal.

The synthesis method also constrains interpretation. Direction-of-effect vote counting was chosen because outcome-definition heterogeneity made pooling inappropriate, but a count of studies does not weight for sample size, precision, or quality. Therefore, a domain in which forty small, favourable series outvote three large, null datasets should not be read as forty-to-three evidence. To address this limitation, future research could use quality-weighted or Bayesian evidence synthesis methods. Future systematic reviews could also focus their main conclusions on high-quality propensity-matched or administrative cohorts, especially for outcomes like respiratory infections. This would mirror the Cochrane approach of separating higher- and lower-quality non-randomised studies. Until then, readers should treat every directional conclusion in this review as qualitative rather than quantitative.

### 4.3 Conclusions

Gastrostomy placement in children with neurological impairment and feeding difficulty is associated with a consistent and clinically meaningful improvement in weight-based nutritional status, and the improvement is greater when the procedure is performed earlier. Serious surgical harm is uncommon when gastrostomy is performed alone. Because of the added surgical risks, concurrent antireflux surgery or jejunal extension should be used cautiously. Following AAP guidance, these additions should be reserved for cases of severe, symptomatic GERD or persistent aspiration.

Gastrostomy should not, however, be presented to families as a means of preventing aspiration or respiratory infection. The largest and most rigorously controlled datasets show no reduction in pneumonia-related hospitalisation, and in some analyses an increase, and this discordance with the smaller before-and-after cohorts is systematic enough to warrant caution in how the procedure is discussed at the point of consent.

The evidence supports a caregiver benefit. The best-evidenced quality-of-life finding in this review is that caregiver health-related quality of life improves and caregiver worry about the child's nutrition falls substantially, measured with a validated instrument against population norms. The corresponding claim about the child cannot be made: it has been tested once with a validated instrument and a baseline, and the total score did not change.

Two further points should be communicated to families: *de novo* reflux is hypothesized to occur in a subset of previously reflux-free children, while reflux indices may improve in some children with pre-existing disease. As such, the clinical conversation should differ according to the child's pre-operative reflux status; most new reflux is managed medically, and fewer than one child in ten goes on to need antireflux surgery, with a seizure disorder the strongest predictor of those who will. And while mortality in this population is high, it reflects the underlying disease rather than the operation: deaths attributable to the procedure are rare enough to be counted individually across the whole literature, thirty-day mortality is 0% to 3% outside the most fragile diagnostic groups, though the two propensity-matched comparisons available disagree: one found short-term mortality halved, the other found it more than doubled, so this review cannot determine whether gastrostomy raises, lowers, or has no effect on survival.

The most important conclusion of this review is negative: there is no rigorously captured data on how gastrostomy impacts a child's cognition, motor function, or communication. To address this evidence gap, future studies need a different approach. Prospective cohorts should use standardized, validated pediatric developmental tools (like the WeeFIM or CP-QOL) and report outcomes stratified by GMFCS levels to adjust for baseline severity. These studies also need clearly defined developmental endpoints and follow-up periods long enough to capture real changes in cognitive and physical development.

Ultimately, these findings highlight the importance of shared decision-making. As emphasized by AAP guidelines on nonoral feeding, clinicians should work with families to weigh the clear benefits of gastrostomy (like nutritional recovery and improved caregiver quality of life) against the remaining clinical uncertainties (like the lack of respiratory protection and unknown neurodevelopmental impacts). By using clear data on surgical safety and the importance of early placement, clinicians can help families make more informed and realistic decisions.

## Data Availability

The original contributions presented in the study are included in the article/Supplementary Material, further inquiries can be directed to the corresponding author/s.
